# Genome-wide estimation of firing efficiencies of origins of DNA replication from time-course copy number variation data

**DOI:** 10.1186/1471-2105-11-247

**Published:** 2010-05-13

**Authors:** Huaien Luo, Juntao Li, Majid Eshaghi, Jianhua Liu, R Krishna Murthy Karuturi

**Affiliations:** 1 Computational and Systems Biology, Genome Institute of Singapore, Biopolis, S138672, Republic of Singapore; 2 Biological Investigations, Genome Institute of Singapore, Biopolis, S138672, Republic of Singapore; 3 Department of Biochemistry, Yong Loo Lin School of Medicine, National University of Singapore, S117597, Republic of Singapore

## Abstract

**Background:**

DNA replication is a fundamental biological process during S phase of cell division. It is initiated from several hundreds of origins along whole chromosome with different firing efficiencies (or frequency of usage). Direct measurement of origin firing efficiency by techniques such as DNA combing are time-consuming and lack the ability to measure all origins. Recent genome-wide study of DNA replication approximated origin firing efficiency by indirectly measuring other quantities related to replication. However, these approximation methods do not reflect properties of origin firing and may lead to inappropriate estimations.

**Results:**

In this paper, we develop a probabilistic model - Spanned Firing Time Model (SFTM) to characterize DNA replication process. The proposed model reflects current understandings about DNA replication. Origins in an individual cell may initiate replication randomly within a time window, but the population average exhibits a temporal program with some origins replicated early and the others late. By estimating DNA origin firing time and fork moving velocity from genome-wide time-course S-phase copy number variation data, we could estimate firing efficiency of all origins. The estimated firing efficiency is correlated well with the previous studies in fission and budding yeasts.

**Conclusions:**

The new probabilistic model enables sensitive identification of origins as well as genome-wide estimation of origin firing efficiency. We have successfully estimated firing efficiencies of all origins in S.cerevisiae, S.pombe and human chromosomes 21 and 22.

## Background

DNA replication is a well-organized process confined to the S phase of cell division cycle. It is initiated at a number of loci called replication origins. During G1 phase, pre-replication complex (pre-RC) is formed at replication origins with the binding of origin-recognition complex (ORC) and initiation factors [1]. In the S phase, DNA replication can be activated from these sites with assistance of protein kinases CDK and DDK. DNA replication origins of different eukaryotes may have different properties. In budding yeast, ORC binds to the 11-bp conserved Autonomously Replicating Sequences (ARS) to initiate the DNA synthesis [2]. In other eukaryotes, consensus sequence is not found and the mechanisms of regulating the function of origins may be determined by other components embedded in the complex genome. An example is fission yeast, which lacks conserved consensus sequence for ORC binding and replication origins are found at A+T rich islands [3]. Current understanding of DNA replication process suggests that DNA replication in each cell is stochastic [4] and there may be much more dormant origins than actually used [5]. During a specified cell cycle, origins may undergo two kinds of replications: they either initiate replication (active replication), or are replicated by the replication forks initiated from neighboring origins (passive replication). The *firing efficiency *of a specific origin is determined as the percentage of cell cycles that it functions actively to initiate replication, i.e., frequency of active replication. Different origins may have different firing efficiencies. Some origins fire in nearly every cell cycle, and some may seldom fire. Measuring origin firing efficiency is important to understand the mechanisms underlying DNA replication. Several methods have been developed to measure and estimate firing efficiencies of DNA replication origins. In [6], Shirahige et al measured ARS' activity from budding yeast in extrachromosomal plasmids through the analysis of mitotic stability. Their results show that these sequences can initiate replication with very high efficiency (>90%) when they are removed from chromosome and incorporated in plasmid. However, when these sequences remained intact in the original chromosomal context, some of them may not fire at all. The firing efficiencies of these potential origins range from very high to very low. In [7], replication origins on chromosome VI of *S.cerevisiae *were studied using 2-D gel electrophoresis. The firing efficiencies of these origins are estimated as the difference of the fraction of replication forks between leaving and entering the origins by studying replication patterns of the DNA fragments immediately flanking the origin. Their estimation of firing efficiencies of 9 replication origins on chromosome VI ranges from less than 10% to more than 85%, with average efficiency of around 42.4%. Yamashita et al [8] validated the above results by using the same technique (2-D gel electrophoresis), but different estimation methods. They estimate firing efficiency by measuring the ratio of the density of bubble arc (which represents active initiation) and simple-Y arc (which represents passive replication). The average firing efficiency they estimated ranges from 37.1% to 48.2% for different strains of yeast. Besides the estimation of origin firing efficiencies of budding yeast, origin firing efficiencies of fission yeast were also determined. Patel et al investigated firing efficiencies of 14 fission yeast origins by using single molecule technique called DNA combing [9]. This technique visualizes stretches of DNA molecules on a glass surface by fluorescence microscopy. The firing efficiencies of origins are determined as the percentage of DNA fragments that contained a bubble within the region of the origin. These techniques work well for small number of origins, but are limited by their incapacity to measure all origins efficiently. Recent genome-wide study of DNA replication approximate origin firing efficiency by indirectly measuring DNA enrichment in hydroxyurea (HU) experiment [10]. Due to the effect of HU, replication forks were stalled at the locations near the firing origins. Hence, signal ratios in the HU experiment may reflect how efficiently the origins are used. When origin usage is efficient, signal ratio should be high; while if the origin is seldom used, the signal ratio would be low. The average efficiency of the firing origins in *S.pombe *mitotic S-phase was estimated to be around 29%. This method provides an approximation to the estimation of firing efficiency, however, as the effect of HU prevents interactions of origins, this method may not reflect properties of firing origins in vivo and may lead to inappropriate estimations.

**Figure 1:**
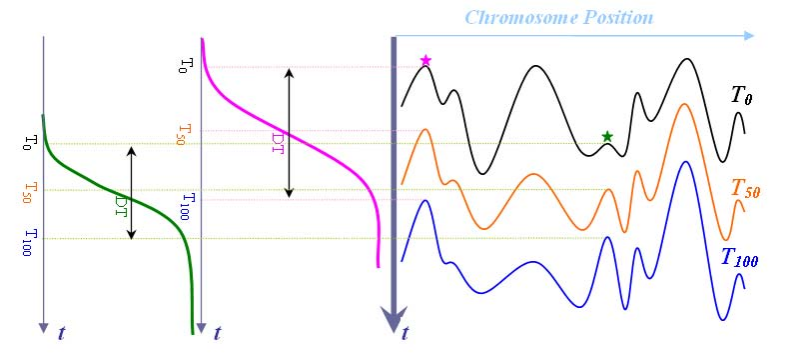
**Illustration of DNA replication profile**. *Left*: DNA content change at an early origin (magenta) and a late origin (green). DNA starts to replicate in a population of cells at time *T*_0 _and gradually increases until it doubles in all cells at time *T*_100 _. The average replication time *T*_50 _approximates the time at which half cells had the origin replicated. The replication span *DT *reflects how fast a given locus can be replicated. Different origins may start to replicate or complete replication at different times with different average replication times or different replication spans. *Right*: Replication profiles *T*_0 _, *T*_50 _and *T*_100 _along the genome. The local maxima (peaks) define the location of origins (an early and a late origin are marked with star), while the local minima (valleys) represents the locations where the replication forks from two flanking origins converge.

As previous methods either lack the ability to measure all origins or are prone to inaccurately approximate origin firing efficiencies, we are seeking a genome-wide analysis method to characterize origins' activities. In this paper, we present a probabilistic model, Spanned Firing Time Model (SFTM), to estimate locations and firing efficiencies of all origins along the whole genome. Different from previous methods, this new method is based on the genome-wide analysis and modeling of DNA replication process. Previous researches demonstrate that DNA replication at a specific locus shows a variability of replication timing in a synchronized cell culture. This variation of replication time was observed in quantitative hybridization experiments [11,12] as well as microarray studies [10,13] which show the copy number change at each locus along the progress in S phase. In [13], replication at a given locus on the genome is modeled to start at *T*_0 _(replication initiation time) and ends at *T*_100 _(replication completion time). The averagereplicationtime or half completion time is *T*_50 _= (*T*_0 _+*T*_100 _)/2 and the replication span is denoted as *DT *= *T*_100 _*- T*_0 _as shown in Figure 1 (left). The replication timing profile which reflects the temporal program of replication can be obtained by joining individual replication time (*T*_0 _or *T*_50 _or *T*_100 _) at different loci alongthe genome (Figure 1, right). In the proposed SFTM, this temporal program of replication as well as the temporal properties of firing origins are characterized and modeled. By fitting SFTM to the experimental time-course S-phase copy number variation data of DNA replication, we could effectively characterize all firing origins. The estimated firing efficiencies are in good consistency with previous measurements using DNA combing and 2D GEL analysis. This research provides a new analytical model to characterize the properties of DNA replication process and a new method to predict origins as well as their firing efficiencies.

## Methods

### Probabilistic Model of DNA replication process

Eukaryotic chromosomes contain multiple replication origins, the activity of which is different in different cells. For the efficient origins, they are utilized in nearly every cell cycle; however, the inefficient origins fire only in a fraction of cell cycles. Therefore, the DNA at a given locus may be replicated by different origins in different cell cycles. If the increase of DNA content at a given locus is viewed as a stochastic event in an ensemble of cell cycles, the probability of DNA replication at a given locus is a function of the probability of initiation of replication by the adjacent origins. For microarray experiments, the measured DNA content change at a given locus *k *at time *t *can be interpreted as the proportion of cells in the cell culture that have already replicated at this locus by time *t*, i.e., probability of replication of locus *k *by time t and represented as a cumulative distribution *P*{*Y*_*k *_≤ *t*}. Suppose the DNA at locus *k *can be replicated by the fork emanating from one of the *m *origins and denote the event of DNA replication at locus *k *replicated by origin *i *before time *t *as {*X*_*ik *_≤ *t*} (*m *= 1, 2,..., *m*), the probability of the DNA replication *P*({*Y*_*k *_≤ *t*}) is determined by the following equation:(1)

which means that the probability of locus *k *replicates before time *t *is the probability of at least one of the *m *replication forks emanating from origin *i *(*i *= 1, 2,..., *m*) and passing locus *k *before time *t*. The probability of each individual origin *i *replicating locus *k *be denoted as:(2)

where constant *f*_*i *_is the potential firing probability of the origin *i *and *P*_*ik *_(*t*) is a function of time *t *and reflects the probability of replication forks emanating from origin *i *(*i *= 1, 2,..., *m*) and passing locus *k *before time *t*.

### Spanned Firing Time Model

Based on the above mathematical description of DNA replication process and temporal flexibility of origin firing observed in the literature, we propose a Spanned Firing Time Model (SFTM) with the following properties:

1. A given origin *O*_*i *_can stochastically fire within a window (*T*_*si *_, *T*_*ei *_) with uniform probability, i.e., *P*_*ii *_(*t*) ~ *U *(*T*_*si *_, *T*_*ei *_) where *T*_*si *_is firing starting time and *T*_*ei *_is firing ending time. *U *(*a, b*) is a function of *t' *and defined as:(3)

2. Each origin can potentially fire with full efficiency 100%.

3. A potential origin will propagate replication if it fires before any replication fork arrives at it. If a replication fork reaches before the origin fires, then initiation will be suppressed and the site of the origin will simply undergo passive replication, which causes the observed origin firing efficiency less than 1.

This model reflects our current understanding of DNA replication processes. For example, in *S.cerevisiae*, the well-defined autonomously replicating sequence (ARS) elements can fire as frequently as 90% outside chromosome in plasmid [6,8,14,15]; however, at their native locations within chromosome, certain ARS elements may not fire or show little origin activity. Studies also show that silent replication origins can be activated if the passive replication by the adjacent origins is prevented [16,17] or if replication forks are slowed down [18,19]. This phenomenon is modeled in the proposed SFTM as that each origin has the full potential to fire (with potential firing probability *f*_*i *_= 1 for each origin) and the passive replication by the replication forks from neighboring origins leads to the incapacity of some origins to fire and hence observed firing efficiency less than 1.

Based on the properties of SFTM, the probability of replication fork emanating from origin *i *and passing locus *k *before time *t *is:(4)

where *T*_*si *_is firing starting time of origin *i*, *T*_*ei *_is firing ending time of origin *i*, *D*_*ik *_is the distance between origin *i *and locus *k*, *v *is replication fork moving velocity and  represents the time for a replication fork to travel from origin *i *to locus *k*.

### Estimation of Firing Efficiency

A basic observation of SFTM is that for locus *k*, the replication initiation and completion time *T*_0 _and *T*_100 _[13] must satisfy the following equations:(5)(6)

This means that for the cells observed in a synchronous cell culture, the DNA at locus *k *starts to replicate at the time when the earliest replication fork emanating from all possible origins arrives, and it finishes replication (for all cells) before or at the time when the first of all possible latest replication forks passes by. Equations (5) and (6) apply to origins as well. The DNA at an origin can be actively replicated by itself, or passively replicated by the adjacent origins. Thereby, the firing time of an origin relative to the replication forks' arriving timing determines its pattern of replication and firing efficiency as well.

**Figure 2:**
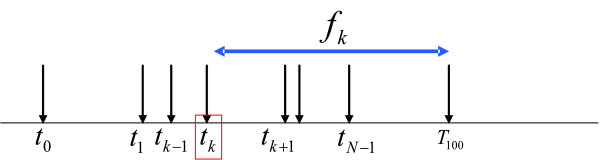
**Illustration of the firing time and its relevance to firing efficiency**. Replication forks/windows from *N *origins can arrive before replication completion time *T*_100 _of origin *k*. Origin *k *starts to fire at time *t*_*k *_. The firing efficiency is then determined from the relative amount of active replication by origin *k *and passive replications by other origins.

Suppose that there are *N *replication forks arriving at a given origin *k *before *T*_100 _, that is, this origin *k *can be potentially replicated by a total of *N *origins (including itself). By ordering of the first arriving time of these forks as shown in Figure 2, where *t*_0 _= *T*_0 _(replication initiation time which is determined by the first of the earliest arriving fork), *t*_*N *_= *T*_100 _(replication completion time which is determined by the first of the possible latest arriving fork) and , we could see that origin *k *can initiate active replication only during the time interval (*t*_*k *_, *T*_100 _) (*t*_*k *_is actually the firing starting time *T*_*s *_of origin *k*) and under the condition that there is no replication forks passing by before origin *k *fires. The probability of the initiation of active replication by origin *k *(or it has the chance to fire) in (*t*_*k *_, *T*_100 _) is then the firing efficiency, which could be calculated according to:(7)

where Δ*T*_*j *_= *T*_*ej *_*- T*_*sj *_is the firing interval of origin *j*. This formula distinguishes between active replication and passive replication. It reflects that origin firing efficiency is a result of interactions between neighboring origins.

### An illustrated example

**Figure 3:**
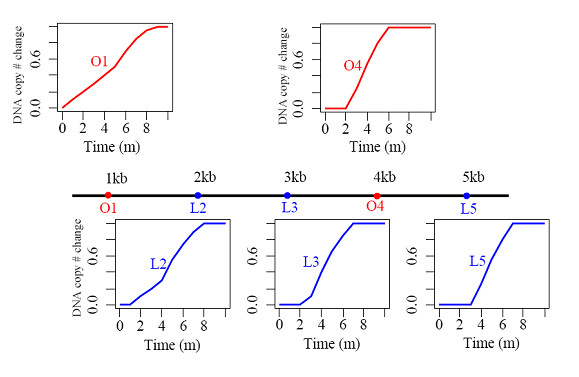
**Illustration of DNA replication process based on Spanned Firing Time Model**. Suppose two origins (red) at locations 1 kbps and 4 kbps fire within the timing window (0,10) and (2,6) (minutes) respectively. The replication fork travels at a velocity of 1 kbps/min. The graphs in the figure are the simulated DNA replication pattern averaged over the cell culture at several positions along the simulated chromosome.

Figure 3 illustrates DNA replication process based on SFTM. In this figure, DNA replication process at five positions along a hypothetical 5 kbps chromosome is simulated. Two origins (red) *O1 *and *O4*, located at positions 1 kbps and 4 kbps, fire within the timing windows of (0,10) and (2,6) (minutes) respectively. The replication fork travels at a velocity of 1 kbps/min to replicate all other positions. For example, the replication forks emanating from origins *O1 *and *O4 *will arrive at the locus *L2 *(at 2 kbps) within the windows of (1,11) and (4,8) respectively. This causes the DNA at *L2 *to start to replicate at time of 1 minute (*T*_0 _= 1) and complete replication at time of 8 minute (*T*_100 _= 8) as shown in Figure 3. In the time interval (1,4), the DNA at *L2 *may be replicated solely by the replication fork from origin *O1 *and in the timing interval (4,8), the two replication forks (from origins *O1 *and *O4*) together contribute to the completion of DNA replication. The DNA replication patterns at these positions shown in Figure 3 display variable durations of replication (*DT*) and it is determined by the firing windows of the related origins, the distance between them and the fork moving rate as shown mathematically in Eqs.(5) and (6).

If *O1 *can only fire very late after 9.5 min within the timing window (9.5, 11) instead of (0,10) in the above example, it will never actively initiate any DNA replication since the replication fork originating from *O4 *will arrive at this position (1 kbps) within the timing window of (5,9). In this case, firing efficiency of *O4 *is 100%, while *O1 *is a dormant(silent) origin with firing efficiency 0%. If replication fork moving rate is reduced to 0.5 kbps/min, the replication fork from *O4 *will arrive at position of *O1 *within the timing window (8,12) (min). Therefore, delayed arrival of replication fork allows for more probability for origin *O1 *to fire, thus increasing the firing efficiency of origin *O1 *to 43.75% according to Eq. (7). If the replication fork moving velocity is further reduced to 0.3 kbps/min, the replication fork from origin *O4 *will arrive at position 1 kbps within the timing window (12,16) (min). As a result, the efficiency of original dormant/inefficient origin *O1 *will increase greatly to 100%. Thus firing efficiency is determined by the firing time window of the interplaying origins, fork replication velocity and relative distance between origins. By incorporating these parameters into SFTM, we could estimate the efficiencies of all origins.

### Algorithm

Procedure to estimate origins as well as their firing efficiency based on SFTM can be implemented in 4 steps as follows.

1. *Data normalization*: Time-course microarray data is first normalized to change from 0 (at the beginning of S phase) to 1 (at the end of S phase).

2. *Estimate fork velocity*: The *T*_50 _replication timing profile is first obtained as described in [13]. The fork moving velocity is then determined using the method in [10]. It is calculated as the ratio of the total distance between the peak and flanking valleys in *T*_50 _replication profile to the total time difference between *T*_50_s at the peak and at the valleys.

3. *Fit SFTM model*: Spanned Firing Time Model needs to estimate the number of origins *m *as well as the location and firing window of each origin. That is, from the time-course microarray experimental data sets, we need to estimate the origin list Θ = {(*L*_*i*, _*T*_*si *_, *T*_*ei *_)|*i *= 1,..., *m*}, where *m *is the number of origins, *L*_*i *_is location of the i*th *origin, *T*_*si *_is firing starting time of the i*th *origin and *T*_*ei *_is firing ending time of the i*th *origin. As the precise number of origins is unknown, it is better to investigate the results obtained from all possible number of origins. Therefore, we start from a range of possible number of origins. This range is roughly estimated from previous studies, for example, a range of 5% to 15% of all available loci for data set *Pom-Heichinger*. For each possible number of origin *m *in this range, we search for the parameters that minimize the sum of squared error (SSE) between the measured DNA content change from microarray experiment and those estimated from our model at all time points and loci. The SSE minimizer is implemented using simulated annealing search [20]. For each search, firing efficiency of each origin is calculated and those origins whose firing efficiency is 0 are discarded.

4. *Calculate regional efficiency*: A regional firing efficiency within a sliding window along the chromosome is calculated for each search. Regional firing efficiency instead of point efficiency is preferred due to the noise and limited resolution of microarray data, variation of the length of DNA replication origin and the inaccurate estimation of the number of origins and so on. This regional firing efficiency is calculated as the probability of active replication of all origins within a window (for example, 3 kbps). The regional firing efficiency estimates are less variable between different starting points used by the algorithm (Additional file 1: Figure S1). Therefore, the calculated regional firing efficiency is averaged over all the searches. The peaks of the averaged regional firing efficiency curve with height greater than 0.05 are then identified and locations where these peaks occur are origins. The firing starting time *T*_*s *_and ending time *T*_*e *_of the origin represented as the peaks are calculated as the median of *T*_*s *_and *T*_*e *_of the found origins at this region identified in all searches.

A brief description of the implementation procedure to obtain the solution of the model parameters is shown in Appendix.

## Results

### Simulation Study

**Figure 4:**
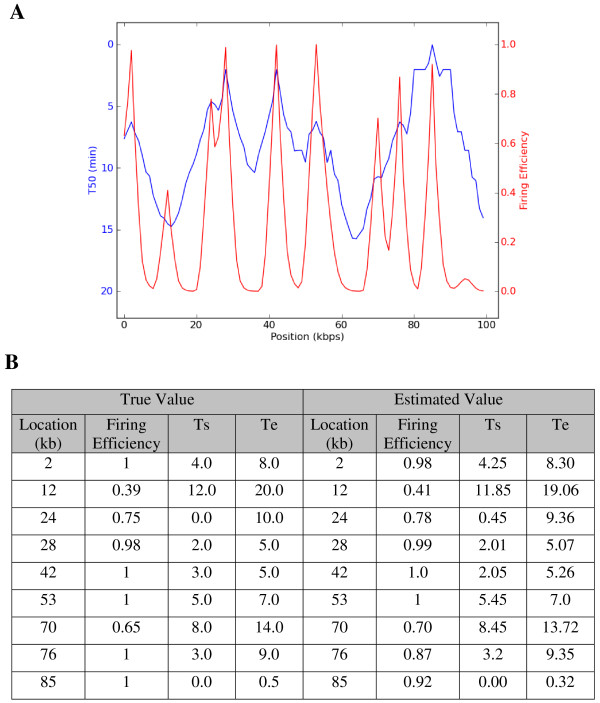
**A simulation study of the proposed model**. (A) Traditional methods estimate the locations of origins from the peaks of *T*_50 _timing profile (shown in blue); while the proposed method estimates locations of origins from the regional firing efficiency curve (shown in red). (B) A comparison between the true value and estimated value of locations of origins, firing efficiency, firing staring time (*T*_*s *_) and ending time (*T*_*e *_).

To test the performance of the proposed algorithm, we have simulated a DNA replication data for a hypothetical chromosome of length 100 kbps with nine origins firing in different timing windows as shown in Figure 4(B). DNA content change at different times at 1 kbps, 2 kbps,..., 99 kbps, 100 kbps are generated. Gaussian noise **N**(0, 0.1) is then added to the generated time-course data. The estimated *T*_50 _timing profileof the generated data is shown as the blue line in Figure 4(A). Traditional methods estimated the locations of origins from *T*_50 _timing profile. However, traditional methods (peak finding) have difficulty in obtaining the correct estimation of some origins such as origins at 12 kbps and 70 kbps. Different from the traditional method, the proposed method estimates the locations of origins from the regional firing efficiency curve as shown in Figure 4(A) (red line). The hardly detected origins (at 12 kbps and 70 kbps) exhibit a clear peak on the regional firing efficiency curve. The small peak at site of 94 kb is a false peak, whose height is less than 0.05, and thus it is excluded from the predicted origins. The results show that the proposed method is more powerful at detecting the locations of origins. Figure 4(B) compares the locations and temporal parameters (*T*_*s *_, *T*_*e *_) of firing origins estimated from the proposed model with the true values used to generate the simulated data. The precise prediction of locations of origins and very low error of the estimation of *T*_*s *_, *T*_*e *_and firing efficiency demonstrate the effectiveness of the algorithm.

### Estimation of Locations of DNA Replication Origins

The validity of the proposed SFTM model is further tested on the available time-course microarray data of DNA replication on both yeasts - *S.pombe *[10,13] and *S. cerevisiae *[21,22]. The brief description and abbreviation of the data sets used is shown in Table 1. The full list of predicted origins is available in Additional file 2. We first compare the locations of origins predicted by SFTM with the previous studies. In previous studies, peaks of the average replication timing profile (normally *T*_50 _timing profile) would reflect location of origins of replication. Whereas, the proposed SFTM estimates locations of origins based on a different scheme: the origin should have a detectable firing efficiency (i.e., if it has the chance to initiate replication, it is an origin). Table 2 displays a comparison of the estimated locations of origins by SFTM and previous studies. It is clearly seen that the proposed SFTM method could effectively find the origins of replication. The obtained results are in good consistency with previous studies using different methods. The average overlapping rate (within 8 kbps window size) between the results from SFTM method and previous studies is 78.8%. The origins identified by SFTM or previous method (peaks identification in average replication timing profile) are also compared to the origins identified by non-replication methods in *S.cerevisiae *[23] and AT richness method in *S.pombe *[3]. The number of overlapping origins and origins identified uniquely by one method is displayed in Additional file 1: Figure S2 and S3. Compared to the previous method, SFTM performs better as the origins detected are more overlapped with those identified by non-replication method or AT richness method. Besides that, the proposed SFTM method is more sensitive by detecting more origins which are not identified by non-replication method or AT richness method. Those origins identified only by SFTM show relatively lower firing efficiencies (Additional file 1: Figure S2(C) and S3(C)). This suggests that SFTM can detect not only strong replication origins but also the weak ones. Table 3 compares the estimated locations of origins by SFTM method applied to different time-course microarray data. The estimations are in good match with each other and this validates the efficacy of the proposed SFTM method in detecting locations of replication origins.

**Table 1:** Time-course Microarray Data Used.

Organism	Abbr.	Reference
***S. cerevisiae***	Cer-Raghuraman	Raghuraman et al (2001) [21]
	
	Cer-Alvino	Alvino et al (2007) [22]

***S. pombe***	Pom-Heichinger	Heichinger et al (2006) [10]
	
	Pom-Eshaghi-Repeat1	Eshaghi et al (2007) [13]
	Pom-Eshaghi-Repeat2	

**Table 2:** Comparison of estimated locations of origins by SFTM and previous studies.

Organism	Method1 # of ORI	In common # of ORI	Method2 # of ORI	Average Distance (kbp)
	Cer-Raghuraman-SFTM 343	256	Cer-Raghuraman 332	3.318
	
***S. cerevisiae***	Cer-Alvino-SFTM 345	261	Cer-Alvino 275	1.877

	Pom-Heichinger-SFTM 589	375	Pom-Heichinger-HU 401	3.727
	
***S. pombe***	Pom-Heichinger-SFTM 589	269	Pom-Feng-cds1 321	3.444
	
	Pom-Eshaghi-SFTM 619	299	Pom-Heichinger-HU 401	3.838
	
	Pom-Eshaghi-SFTM 619	233	Pom-Feng-cds1 321	3.178

This table shows the extent of the match between locations of potential origins estimated by SFTM and previous studies. Each item in column 2 and 4 contains two parts: the representation of the method/paper used to detect the origins and the number of origins detected. Column 2 is the results from proposed SFTM method applied to the time-course microarray data as shown in Table 1. Column 4 is the results obtained from previous studies by Raghuraman et al ("Cer-Raghuraman") [21], Alvino et al ("Cer-Alvino") [22], Heichinger et al ("Pom-Heichinger-HU") [10] and Feng et al ("Pom-Feng-cds1") [28]. Column 3 displays the number of origins in common within 8 kbp distance estimated by the methods in column 2 and 4. The average distance of the origins in common are displayed in column 5.

**Table 3:** Comparison of estimated locations of origins by SFTM using different data sets.

Organism	Dataset1 # of ORI	In common # of ORI	Dataset2 # of ORI	Average Distance (kbp)
***S. cerevisiae***	Cer-Raghuraman 343	263	Cer-Alvino 345	3.373

	Pom-Eshaghi-Rep1 619	431	Pom-Heichinger 589	3.447
	
***S. pombe***	Pom-Eshaghi-Rep2 650	444	Pom-Heichinger 589	3.399
	
	Pom-Eshaghi-Rep1 619	535	Pom-Eshaghi-Rep2 650	2.614

This table shows the extent of the match between locations of potential origins estimated by SFTM applied to different time-course data as shown in Table 1. The meaning of the items in each column is the same as that shown in Table 2.

### Estimation of Firing Efficiency of Origins

#### S.cerevisiae

**Figure 5:**
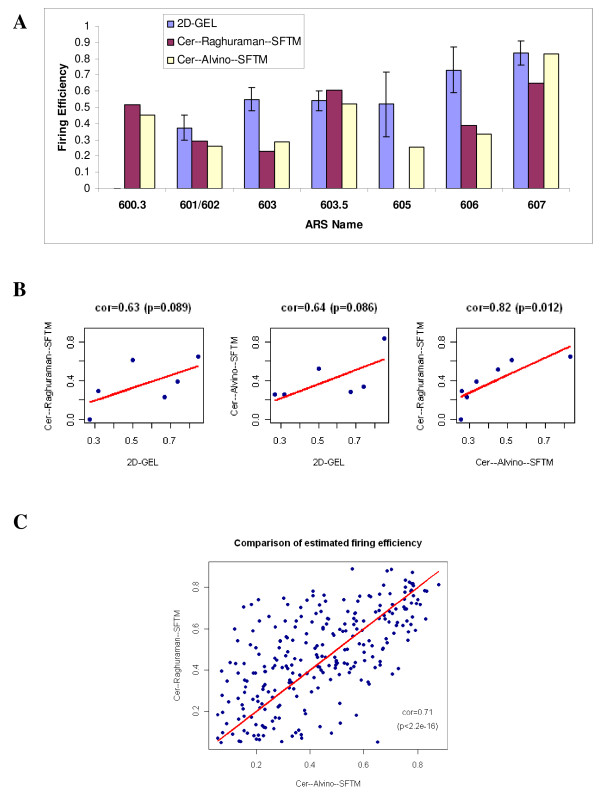
**Comparison of estimated firing efficiencies of origins in S.cerevisiae**. (A) Estimated firing efficiencies of origins on Chromosome VI of *S.cerevisiae*. The height of the vertical bar shows our estimation by applying the proposed SFTM to two microarray timecourse data sets (*Cer-Raghuraman *and *Cer-Alvino*) and Friedman's estimation by using 2-D gel electrophoresis [7]. The error bar of 2-D gel estimation reflects the variation in firing efficiencies among different strains. (B) Pairwise scatter plots of origin firing efficiencies estimated from three sources in (A). The correlation coefficients are shown at the top of the respective figures. The good consistency between these results suggests that SFTM is a valid method to estimate firing efficiency. (C) Scatter plot of the estimated firing efficiencies of the matched origins from two *S.cerevisiae *data sets *Cer-Raghuraman *and *Cer-Alvino *by applying SFTM.

We first compared the firing efficiencies estimated by SFTM applied to the microarray time-course data about DNA replication (*Cer-Raghuraman *and *Cer-Alvino*) with previous quantitative measurements of firing efficiency by using 2-D gel electrophoresis on Chromosome VI of *S.cerevisiae *[7]. Figure 5(A) and 5(B) show a comparison between these estimations. As shown in Figure 5(B), our two estimations correlate well with the previous 2-D gel electrophoresis studies. The pearson correlation coefficients between these three estimations are respectively 0.63 (*p *= 0.089) for 2D-GEL vs Cer-Raghuraman-SFTM; 0.64 (*p *= 0.086) for 2D-GEL vs Cer-Alvino-SFTM and 0.82 (*p *= 0.012) for Cer-Alvino-SFTM vs Cer-Raghuraman-SFTM. These fairly good correlations between our estimation and previous studies indicate the capacity of the proposed SFTM method to estimate firing efficiencies of origins. Next, a comparison is carried out on the estimated firing efficiencies from all 16 chromosomes of *S.cerevisiae *from data sets *Cer-Raghuraman *and *Cer-Alvino*. Figure 5(C) demonstrates the comparison between results obtained from these two data sets. Each point represents one of 263 co-localized origins predicted from these two data sets (see Table 3). From this figure, we could see that the estimations from these two independent microarray data sets are correlated well with each other and the pearson correlation coefficient is 0.71 (*p *< 2.2*e-*16).

#### S.pombe

**Figure 6:**
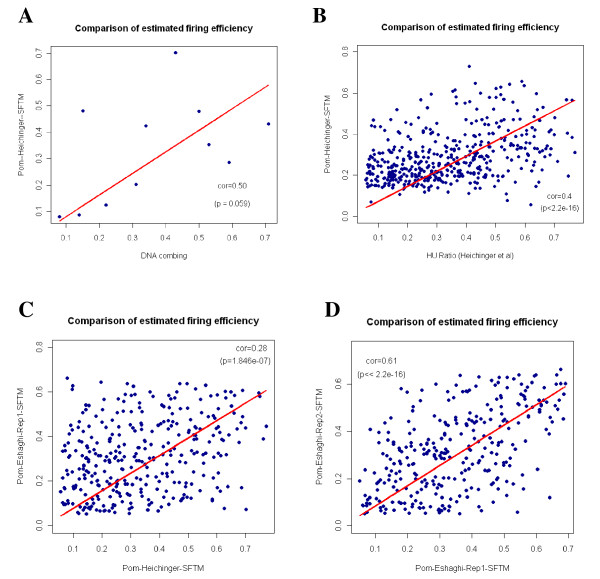
**Comparison of estimated firing efficiencies of origins in S.pombe**. (A) Comparison of estimated *S.pombe *origin efficiencies from single DNA molecular technique (Patel et al, 2006) and the proposed SFTM model applied to the *Pom-Heichinger *microarray data. The efficiencies of 11 origins estimated by both methods are plotted against each other. (B) Comparison of estimated origin efficiencies by measuring the signal ratio in the HU experiment (Heichinger et al, 2006) and the proposed SFTM model applied to the *Pom-Heichinger *time-course microarray data. (C) Comparison of estimated origin efficiencies by applying the proposed SFTM model to two microarray datasets: *Pom-Heichinger *and *Pom-Eshaghi*. (D) Comparison of estimated origin efficiencies by applying the proposed SFTM model to the two repeats of DNA replication dataset *Pom-Eshaghi*. The red line in these figures shows a linear fitting (without intercept) of the two estimations illustrated in each figure. The good consistency between these results indicates that SFTM is a reliable and valid method to estimate firing efficiency at genomic scale.

In [9], Patel et al estimated the firing efficiencies of selected origins on chromosome III of fission yeast. In this part, we first compare our estimation by applying SFTM to the *Pom-Heichinger *data set with those estimated by Patel et al. Figure 6(A) shows a comparison of efficiencies of 11 origins estimated by both methods. The correlation coefficient between these two estimations is 0.5 (*p *= 0.059). This result validates the efficacy of the proposed method in estimating firing efficiencies of origins.

Next, we compared our estimation of the origin firing efficiencies with those estimated by measuring the signal ratios in the HU experiment from Heichinger et al [10]. These two estimations are well correlated with each other and the correlation coefficient is around 0.4 (*p *< 2.2*e *- 16) as shown in Figure 6(B). Figure 6(C) compares the estimated firing efficiencies of matched origins by applying the proposed SFTM model to the two microarray datasets: *Pom-Heichinger *and *Pom-Eshaghi*. The two estimations are correlated with each other but not strongly correlated (correlation coefficient 0.28; *p *= 1.85*e *- 07). This may be due to the different synchronization methods used in these two experiments. In *Pom-Heichinger*, the cell culture is synchronized using temperature-sensitive cdc25-22 mutant at the entry of G2 phase; while in *Pom-Eshaghi*, synchronization using hydroxyurea (HU) at the beginning of S phase is obtained. In addition, the origin interaction is not taken into account in Heichinger et al's analysis [10]. The origin efficiency in Heichinger et al's analysis is estimated from DNA enrichment ratio in the HU experiment. As HU will inhibit the fork migration away from origins, the origin interaction is actually not taken into account in their analysis. However, in our model, the origin interaction is the key to explain and estimate origins' firing efficiency. Figure 6(D) demonstrates comparison results obtained from the two repeats in *Pom-Eshaghi*. The correlation between these two repeats is 0.61(*p *< 2.2*e *- 16).

The above results obtained from *S.cerevisiae *and *S.pombe *microarray experimental data are in good consistency with previous studies which estimate origins firing efficiencies by using DNA combing technique or 2-D gel electrophoresis. Besides that, results obtained by applying the proposed SFTM to the microarray data sets from different labs also demonstrate a good correlation. From these results, we conclude that the proposed SFTM is a reliable and valid method to estimate origin firing efficiency (or origin usage frequency).

### Distributions of Origin Firing Efficiency

**Figure 7:**
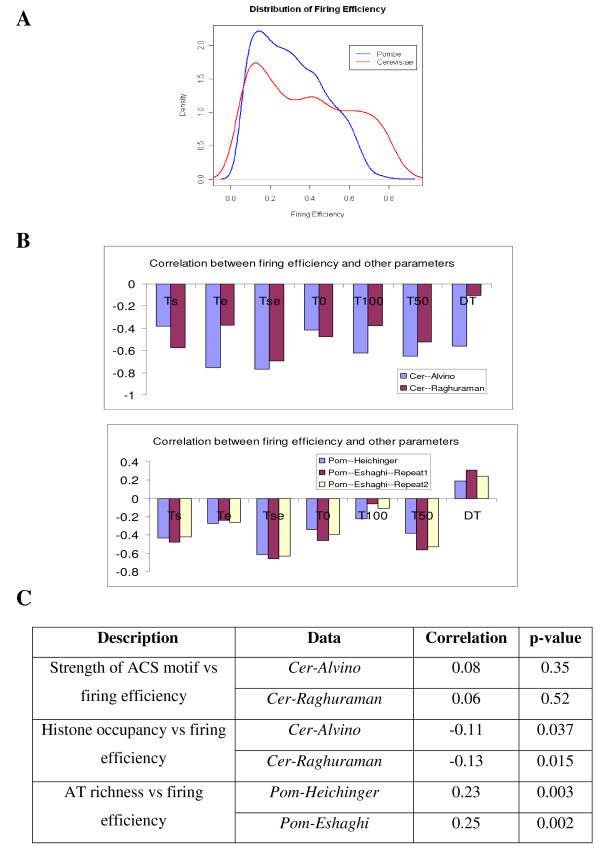
**Characterizing the temporal parameters of origins firing/replication**. (A) Distributions of origin firing efficiency in *S.cerevisiae *and *S.pombe*. (B) Correlation between firing efficiency and other parameters: *T*_*s *_(firing starting time), *T*_*e *_(firing ending time), *T*_*se *_(average firing time), *T*_0 _(replication initiation time), *T*_100 _(replication completion time), *T*_50 _(average replication time) and *DT *(replication span). (up) Correlation obtained from two DNA replication microarray data sets of *S.cerevisiae*: *Cer-Raghuraman *and *Cer-Alvino*; (bottom) Correlation obtained from microarray data sets of *S.pombe*: *Pom-Heichinger *and *Pom-Eshaghi-Repeat1/2*. (C) Correlation between origin firing efficiency and the factors that may regulate efficiency: strength of ACS motif, Histone occupancy level and AT richness around origins.

Figure 7(A) compares the distribution of firing efficiencies in *S.cerevisiae *and *S.pombe*. It is clearly seen from this figure that *S.cerevisiae *contains larger proportions of efficient origins (efficiency > 0.6) than *S.pombe*. This conclusion is in consistency with previous studies which state that origins in *S.cerevisiae *are more efficient than those in *S.pombe*. Our estimations of origin firing efficiency demonstrate that the average firing efficiency is around 0.38 in *S.cerevisiae *and around 0.30 in *S.pombe*, which is in consistent with previous estimates (37.1%-48.2% in *S.cerevisiae *[8] and 29% in *S.pombe *[10]).

### Firing Efficiency and Replication/Firing Time

Finally, we investigated the properties of parameters related to DNA replication process in SFTM. Figure 7(B) shows the corresponding correlation between origin firing efficiency and origin firing starting/ending times, origin replication initiation/completion times and so on. As illustrated in Figure 7(B), *T*_*s *_and *T*_*e *_are negatively correlated with firing efficiency, which means that if an origin starts to fire early, it is efficient and if it finishes firing early, it is efficient. This is because that an origin may have more chance to actively initiate the replication if it can start/end firing early. However, if the firing window (*T*_*s *_, *T*_*e *_) of an origin is relatively late compared to other origins, the late firing time of this origin may render the replication fork emanating from neighboring origins more time to travel to this location and replicate it. Thus, this origin would be inefficient since it is less likely for this origin to initiate the replication actively. Another observation from Figure 7(B) is that average replication time (*T*_50 _) is highly negatively correlated with firing efficiency and hence it is reasonable to approximate firing efficiency by only calculating the average replication time (*T*_50 _). Previous studies [24,25] traditionally classify origin to be strong/weak (or efficient/inefficient) based on whether it is early/late replicated. The negative correlation between firing efficiency and average replication time validates this classification and approximation. It is also seen from Figure 7(B) that the replication span (*DT*) is negatively correlated with firing efficiency in *S.cerevisiae *and positively correlated with firing efficiency in *S.pombe*. The replication span is used to describe replication efficiency of a locus (i.e., how fast a locus can complete the replication and defined as in ) [13]. The negative correlation in *S.cerevisiae *means that *S.cerevisiae *has replication efficient and firing efficient origins. To be specific, if an origin can initiate the active replication more frequently in cell cycle (i.e., more firing efficient), it may take shorter time for this origin to finish the replication (i.e., more replication efficient). Whereas, in *S.pombe*, it may take longer time for the firing efficient origins to complete the replication (i.e., replication inefficient) because of the positive correlation between firing efficiency and replication span. This means that *S.pombe *has either replication efficient or firing efficient origins but not both. These results demonstrate that the relationship between firing efficiency and replication span (or replication efficiency) depends on the context and the organism.

The above observation from SFTM can also explain the discrepancy about "origin efficiency" reported in previous studies. In [10], Heichinger et al observed a negative correlation between origin efficiency and replication time; while Eshaghi et al observed a positive correlation between origin efficiency and replication time. This discrepancy comes from different definitions of origin efficiency. In Heichinger et al's paper, origin efficiency is defined as the frequency of origin usage in the cell population or cell cycles. Therefore, an efficient origin would fire in most cell cycles, i.e. fire efficiently. The negative correlation observed between firing efficiency and average replication time (*T*_50 _)as shown in Figure 7(B) agrees with Heichinger's results that origin efficiency is negatively correlated with replication time and the early origins tend to be more efficient than late ones. Whereas, Eshaghi et al approximated origin efficiency by how fast an origin replicates, i.e. replication rate or replication efficiency (). Higher replication efficiency means shorter replication span *DT*. Based on SFTM, a positive correlation between *T*_50 _and  is observed.

Thus, the same conclusion is reached in our and Eshaghi et al's research that the late origins have higher replication rate (shorter *DT*) in order to complete the DNA replication in time. By distinguishing the two 'origin efficiency' in previous research as 'firing efficiency' (*f*_*i *_) and 'replication efficiency' (), we successfully solved the discrepancies incured in [10] and [13].

Next, we explored the factors which may regulate firing efficiency (Figure 7(c)). In *S.cerevisiae*, ARS consensus sequence (ACS) is the motif of the binding site for replication initiator protein. We first calculated the correlation between the strength of ACS motif [23] and the firing efficiency of origins. As shown in Figure 7(c), no significant correlation is found between ACS strength and firing efficiency. However, the consistent positive value may suggest the weak positive influence of ACS strength on firing efficiency. Next, we examined the correlation between histone occupancy and firing efficiency. The histone occupancy data is obtained from [26]. The negative and significant correlation (-0.11 for "Cer-Alvino" and -0.13 for "Cer-Raghuraman") demonstrates that origins located in regions with lower histone occupancy have higher firing efficiency compared to origins with higher histone occupancy. This may be due to the easy accessibility to replication proteins at the regions with lower histone occupancy. At last, we studied the relation between origin firing efficiency and AT richness in *S.pombe*. As demonstrated in Figure 7(c), a significant and positive correlation is observed (0.23 for "Pom-Heichinger" and 0.25 for "Pom-Eshaghi"). This positive correlation demonstrates that the regions with higher AT content are generally firing efficient compared to regions with lower AT content.

### Origin Efficiency Estimation on Human Chromosome 21 and 22

**Figure 8:**
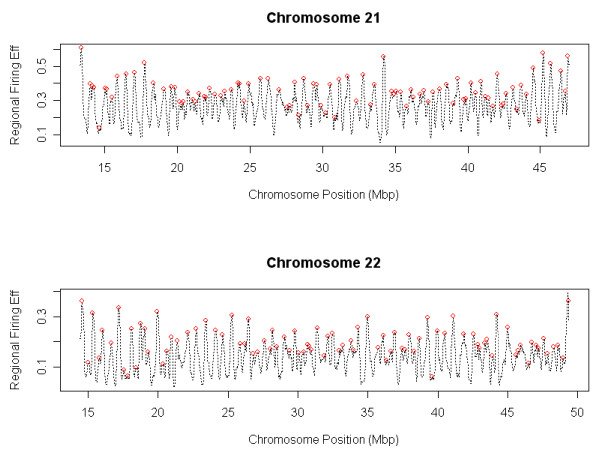
**Estimated locations and firing efficiencies of origins on chromosome 21 and 22 of human**. Dashed line represents the estimated regional firing efficiency curve. Origins are located at the peaks where the height represents the firing efficiency of each origin (red circles).

The proposed SFTM model is also applied to the human DNA replication data sets [27] to determine locations and firing efficiencies of the origins. Figure 8 shows the results averaged across three available repeats [27]. The regional firing efficiency is calculated based on a region window of 50 kbp. The peaks of regional firing efficiency curve provide the information about locations and firing efficiencies of origins. The average firing efficiencies are respectively 34.8% and 31.4% for chromosome 21 and 22. An observation from Figure 8 is that firing efficiency shows a domain effect. In some domains, such as the region from 15 to 20 Mbps on chromosome 21, the distances between origins are large and firing efficiencies for them are also high. In other domains, such as the region from 20 to 25 Mbps on chromosome 21, the distances between origins are small and many relatively inefficient origins exist. If average firing efficiency at each domain is calculated, the variation of average firing efficiency is small which demonstrates that each domain is at the same level of firing efficiency in order to complete the DNA replication in time.

## Discussion and Conclusion

In this paper, we developed a new probabilistic model - Spanned Firing Time Model (SFTM) for DNA replication. The proposed SFTM model reflects constrained stochastic properties in DNA replication. A key feature in SFTM is that each origin has two characteristic times: firing starting time *T*_*s *_and firing ending time *T*_*e *_, which determine the span of the firing window as well as the relatively early/late firing. The random firing of origins within the firing window will make different cells undergo different replication program, and hence "stochasticity" in DNA replication. The difference of the firing span length and firing starting time may cause the population average displaying a temporal program with some regions replicating early and some regions replicating late.

Based on the proposed SFTM model, we could estimate origins' firing efficiencies at genome-wide scale from time-course S-phase DNA copy number variation data. Our results obtained from both *S.cerevisiae *and *S.pombe *demonstrate that the predicted origins and their efficiencies are in good consistency with previous studies as well as among themselves. These results reveal that the proposed model is appropriate to characterize the properties of DNA replication process and estimate the origin usage in the cell populations. The proposed SFTM model is also applied to human DNA replication data on chromosomes 21 and 22 as the spanned firing of origins should reflect a general phenomenon of DNA replication process and is not confined to the yeasts.

The proposed SFTM model can estimate the locations as well as the firing efficiencies of origins at genomic scale. Compared to the conventional genome-wide methods which detect locations of origins from *T*_50 _temporal profile, SFTM is more sensitive to detect weak origins as shown in Additional file 1: Figure S2 and S3. A point needs to be noted is that our estimation of firing efficiencies is based on the genome-wide time-course data. For the analysis of an individual origin, the experimental methods will be more accurate in estimating the firing efficiency. Therefore, the proposed method is not meant to replace direct firing efficiency measurement methods (which are cumbersome for genome-wide estimation) for accuracy, but instead provides an efficient alternative way to estimate the firing efficiency of origins at genomic scale, though at reduced accuracy. As current microarray time-course S-phase DNA copy number variation data suffers from limited temporal and spatial resolution, the accuracy of the proposed method in estimating origins' locations and efficiencies are affected from these limitations. We can imagine that a sufficient sampling rate on densely probed time-course data will significantly improve the accuracy of our estimates. In addition, as an improvement of the proposed model, the underlying assumptions will be examined in our future work. For example, the assumption of uniform distribution of origin firing timing within a time window may be improved by a possible empirical distribution. Moreover, a good estimation of the replication fork moving velocity is also important for the accuracy of our estimations. The current method estimated the fork moving velocity from the slope of the *T*_50 _curve. This is a first-order approximation to the fork velocity, as it does not consider the contribution of different fork moving directions to the shape of *T*_50 _curve. This can be improved in our future work by investigating the dynamics of replication fork progression. The stochastic properties of DNA replication process is summarized and reflected by the parameters (*T*_*s *_, *T*_*e *_, etc) in our proposed model. In our future work, the relation between the molecular factors (cis- and trans-acting) and these summarized temporal parameters will be investigated. We hope that our results will be useful for the further analysis of the stochastic yet robust program of DNA replication process.

## Authors' contributions

RKMK and JL proposed the project; HL and RKMK developed the model, implemented the algorithm and wrote the paper. All the authors evaluated the results and approved the manuscript.

## Appendix

### Implementation of SFTM algorithm

**Input**: Microarray Experimental Data obtained at several time points

**Output**: ORI List Θ = {(*L*_*i *_, *T*_*si *_, *T*_*ei *_)*|i *= 1,..., *m*}

Parameters:

*m*: number of origins

*L*_*i *_: location of the i*th *origin

*T*_*si *_: firing starting time of the i*th *origin

*T*_*ei *_: firing ending time of the i*th *origin

Algorithm

1. Set a range of possible number of origins.

2. For each *m *in this range, start from different initial point, search for the best parameter triplets (*L*_*i *_, *T*_*si *_, *T*_*ei *_) using optimization procedure which could minimize the SSE between observed and estimated DNA content at all time-points and loci.

3. For each search from different starting point, calculate the firing efficiency of each origin according to Eq. (7) and discard those whose firing efficiency is 0. After that, obtain the regional firing efficiency curve for each search.

4. Repeat 2-3 for different number of origins.

5. Obtain the average regional firing efficiency curve as the average over all the searches.

6. Find the peaks of the average regional firing efficiency curve (the peaks with amplitude less than 0.05 is discarded), the number of the peaks is then the total number of origins *m *and the locations of the peaks are the parameter *L*_*i *_. The amplitude of the peak is then the firing efficiency estimated for this origin.

7. *T*_*si *_and *T*_*ei *_are calculated as the median of the firing starting and ending time of the origins identified in this region for all searches.

## Supplementary Material

Additional file 1 **Supplementary figures**. This file contains supplementary figures: Figure S1, S2 and S3. Click here for file

Additional file 2 **Predicated origins of *S.cerevisiae*, *S.pombe *and Human chromosomes 21 and 22**. This file contains the tables of predicted origins of *S.cerevisiae*, *S.pombe *and Human chromosomes 21 and 22. Each table contains the following parameters related to origins: locations, firing efficiency (*F*_*i *_), firing starting time (*T*_*s *_), firing ending time (*T*_*e *_), replication initiation time (*T*_0 _), replication completion time (*T*_100 _), average replication time (*T*_50 _) and replication timing span (*DT*). Click here for file
