# Supplementary material for: Genome-wide estimation of firing efficiencies of origins of DNA replication from time-course copy number variation data
Source: BMC Bioinformatics. 2010 May 13;11:247. doi: 10.1186/1471-2105-11-247 (PMC2885374; doi:10.1186/1471-2105-11-247)
Supplement: Additional file 1 — Supplementary figures. This file contains supplementary figures: Figure S1, S2 and S3. [file 1471-2105-11-247-S1.DOC]

Supplementary Figures


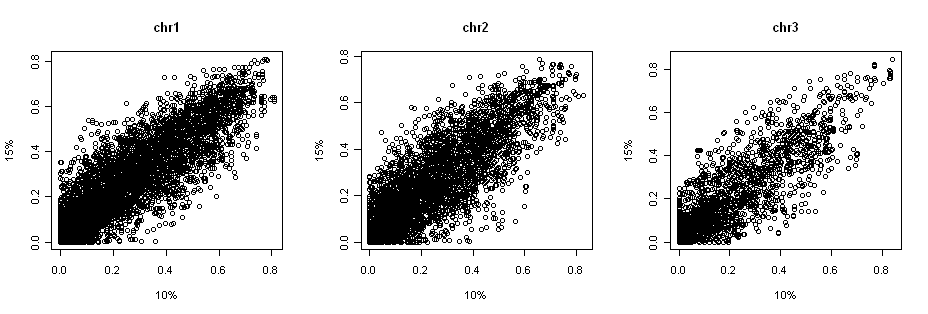


**Figure S1:** The regional firing efficiency is less variable among different searches with different starting points. This figure shows the comparison of results from two searches (10% and 15% of total loci as the potential number of origins) by applying the proposed algorithm to the data set *Pom-Heichinger*. Each point in this figure represents two estimations of regional firing efficiency at a locus using 10% and 15% of total loci as the potential number of origins. All the loci on chromosome 1, 2 and 3 of S. pombe are plotted. The correlations are 0.87, 0.83 and 0.88 for chr1, chr2 and chr3 respectively. It is seen from this figure that different starting parameters will arrive at similar regional firing efficiency. The average of these searches will provide us a better result.

186

42

159

Alvino_SFTM

Nieduszynski

185

43

90

Alvino_previous

Nieduszynski

66

181

Raghuraman_SFTM

Nieduszynski

161

67

171

Nieduszynski

Raghuraman_previous

162

**B**

**A**


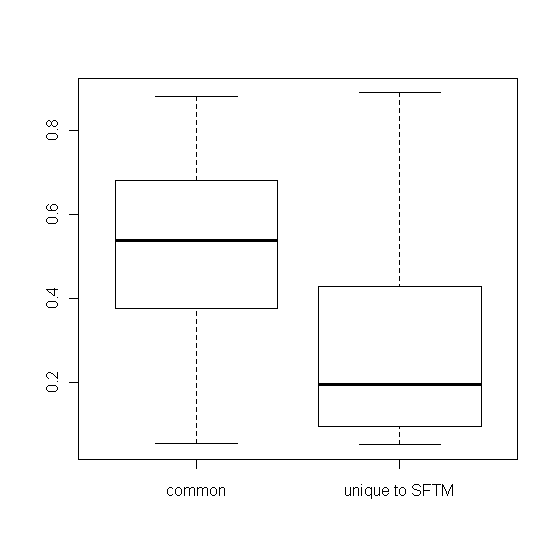

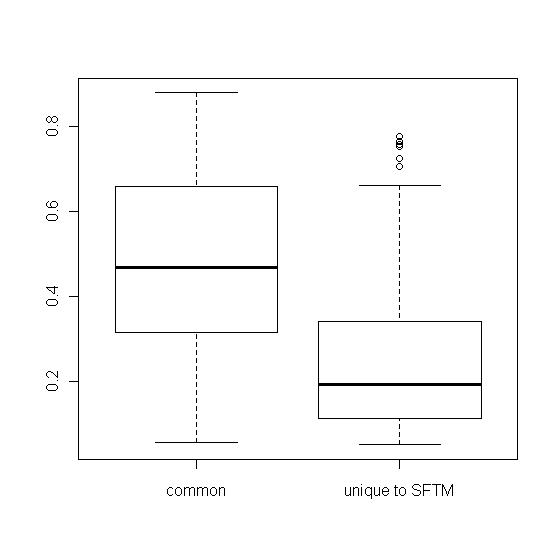


Firing Efficiency

Firing Efficiency

**C**

Raghuraman_SFTM

Alvino_SFTM

**Figure S2:** A comparison between the origins identified using SFTM and previous non-replication methods by Nieduszynski C.A. [1].

**(A**) Venn diagram of overlap between origins identified by Nieduszynski C.A. [1] and origins identified by our proposed SFTM applied to microarray data sets “Cer-Alvino” (Alvino_SFTM) and “Cer-Raghuraman” (Raghuraman_SFTM).

**(B)** Venn diagram of overlap between origins identified by Nieduszynski C.A.[1] and origins identified by Alvino [2] (Alvino_previous) and Raghuraman [3] (Raghuraman_previous).

**(C)** Boxplots of estimated firing efficiencies of overlapping origins and origins identified only by SFTM as defined in (A). The overlapping origins are generally strong origins with higher firing efficiency.

256

128

333

Heichinger_SFTM

Segurado

228

156

173

Heichinger_previous

Segurado

150

385

Eshaghi_SFTM

Segurado

181

203

335

Segurado

Eshaghi_previous

234

**B**

**A**


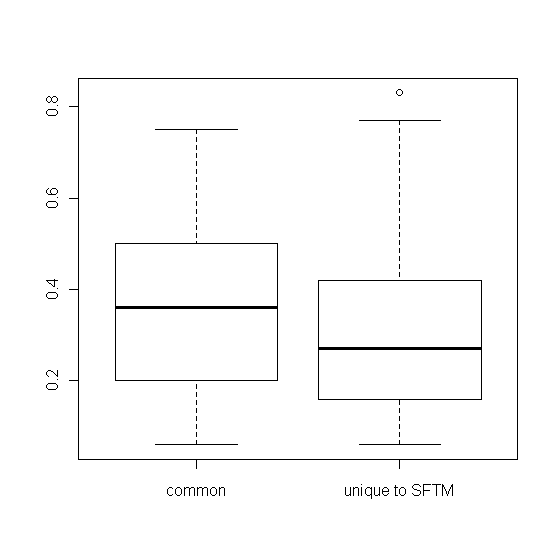

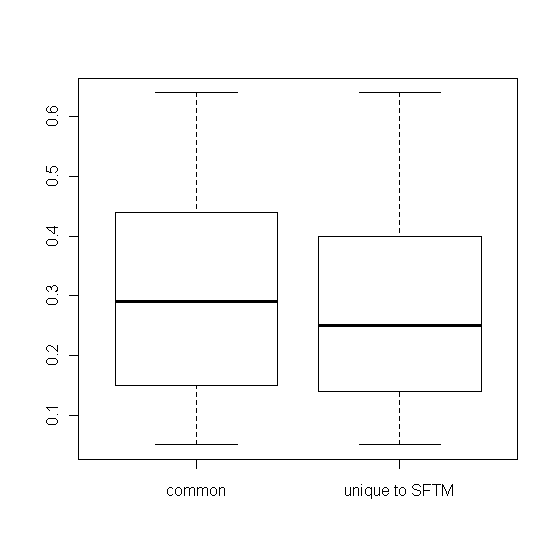


Firing Efficiency

Firing Efficiency

**C**

Heichinger_SFTM

Eshaghi_SFTM

**Figure S3:** A comparison between the origins identified using SFTM and AT richness computed by Segurado [4].

**(A**) Venn diagram of overlap between origins identified by Segurado [4] and origins identified by our proposed SFTM applied to microarray data sets “Pom-Heichinger” (Heichinger_SFTM) and “Pom-Eshaghi” (Eshaghi_SFTM).

**(B)** Venn diagram of overlap between origins identified by Segurado [4] and origins identified by Heichinger [5] (Heichinger_previous) and Eshaghi [6] (Eshaghi_previous).

**(C)** Boxplots of estimated firing efficiencies of overlapping origins and origins identified only by SFTM as defined in (A). The overlapping origins are generally stronger origins with higher firing efficiency.

**Reference:**

# [1] Nieduszynski CA, Knox Y, Donaldson AD: Genome-wide identification of replication origins in yeast by comparative genomics. *Genes Dev* 2006, 20: 1874-1879.

# [2] Alvino G, Collingwood D, Murphy J, Delrow J, Brewer B, Raghuraman M: Replication in Hydroxyurea: It’s a matter of time. *Molecular and Cellular Biology* 2007, 27:6396–6406.

# [3] Raghuraman M, Winzeler E, Collingwood D, Hunt S, Wodicka L, Conway A, Lockhart D, Davis R, Brewer B, Fangman W: Replication dynamics of the yeast genome. *Science* 2001, 294:115–121.

# [4] Segurado M, de Luis A, Antequera F: Genome-wide distribution of DNA replication origins at A+T-rich islands in Schizosaccharomyces pombe. *EMBO Rep.* 2003, 4:1048–1053.

# [5] Heichinger C, Penkett C, Bahler J, Nurse P: Genome-wide characterization of fission yeast DNA replication origins. *Molecular Biology of the Cell* 2006, 25:5171–5179.

# [6] Eshaghi M, Karuturi R, Li J, Chu Z, Liu E, Liu J: Global profiling of DNA replication timing and efficiency reveals that efficient replication/firing occurs late during S-phase in S.pombe. *PLoS ONE* 2007, 2:e722.
