# Supplementary material for: Genome-wide estimation of firing efficiencies of origins of DNA replication from time-course copy number variation data
Source: BMC Bioinformatics. 2010 May 13;11:247. doi: 10.1186/1471-2105-11-247 (PMC2885374; doi:10.1186/1471-2105-11-247)
Supplement: Additional file 2 — Predicated origins of S.cerevisiae, S.pombe and Human chromosomes 21 and 22. This file contains the tables of predicted origins of S.cerevisiae, S.pombe and Human chromosomes 21 and 22. Each table contains the following parameters related to origins: locations, firing efficiency (Fi ), firing starting time (Ts ), firing ending time (Te ), replication initiation time (T0 ), replication completion time (T100 ), average replication time (T50 ) and replication timing span (DT). [file 1471-2105-11-247-S2.PDF]

:er_Alvino

Index	Chr	Location (kb)	Fi	Ts	Te	T0	T100	T50	DT
1	1	12	0.56	7.38	57.99	7.38	49.02	28.20	41.64
2	1	42	0.12	7.79	49.24	7.82	49.22	28.52	41.40
3	1	71	0.31	5.34	75.00	5.46	47.78	26.62	42.32
4	1	169	0.62	2.56	51.35	2.56	26.62	14.59	24.06
5	2	32	0.33	5.64	68.40	5.72	48.25	26.99	42.53
6	2	56	0.16	37.70	59.32	3.92	41.65	22.79	37.73
7	2	65	0.41	2.54	53.43	2.52	38.00	20.26	35.48
8	2	114	0.19	49.28	75.00	3.08	67.83	35.46	64.75
9	2	143	0.10	4.56	72.84	4.50	47.22	25.86	42.73
10	2	168	0.31	3.33	75.00	3.24	45.13	24.19	41.89
11	2	187	0.07	4.39	75.00	4.39	43.37	23.88	38.98
12	2	196	0.13	4.62	75.00	4.62	34.28	19.45	29.66
13	2	235	0.67	3.82	55.86	-3.05	27.60	12.27	30.65
14	2	313	0.10	3.49	69.63	3.49	48.33	25.91	44.83
15	2	323	0.39	1.90	58.89	1.90	45.18	23.54	43.27
16	2	355	0.11	3.43	75.00	3.43	58.45	30.94	55.02
17	2	382	0.28	3.38	60.71	3.31	43.84	23.58	40.53
18	2	408	0.30	6.57	54.60	6.57	30.78	18.68	24.20
19	2	422	0.16	5.79	74.51	5.79	39.37	22.58	33.58
20	2	441	0.15	4.93	75.00	4.99	56.08	30.53	51.09
21	2	492	0.47	5.39	53.36	5.89	37.70	21.79	31.81
22	2	515	0.06	37.86	57.57	8.05	48.47	28.26	40.42
23	2	550	0.34	4.61	75.00	4.61	64.84	34.72	60.23
24	2	589	0.07	7.85	75.00	7.62	44.59	26.11	36.97
25	2	613	0.15	32.74	75.00	9.11	33.57	21.34	24.46
26	2	624	0.75	6.83	44.09	6.83	28.24	17.53	21.41
27	2	706	0.21	7.16	73.82	7.16	47.18	27.17	40.02
28	2	713	0.15	40.69	66.61	7.96	45.82	26.89	37.86
29	2	720	0.12	8.11	48.52	8.12	46.52	27.32	38.41
30	2	742	0.42	6.82	60.17	6.80	45.96	26.38	39.16
31	2	775	0.25	6.78	68.92	6.78	45.44	26.11	38.66
32	2	784	0.37	7.48	49.16	7.53	46.98	27.25	39.45
33	3	35	0.68	1.62	44.26	0.14	25.72	12.93	25.58
34	3	73	0.56	-2.96	47.66	-2.96	28.88	12.96	31.84
35	3	117	0.19	2.26	66.37	-0.65	37.20	18.28	37.85
36	3	126	0.10	5.20	75.00	3.23	35.95	19.59	32.72
37	3	164	0.44	-0.90	59.93	-0.90	44.27	21.69	45.17
38	3	202	0.09	37.73	75.00	3.66	38.59	21.13	34.93
39	3	222	0.56	4.48	52.43	4.21	28.04	16.13	23.83
40	3	278	0.60	3.11	54.23	3.11	48.53	25.82	45.41
41	4	18	0.22	35.39	53.85	8.47	53.43	30.95	44.96
42	4	41	0.63	6.87	55.45	6.87	50.74	28.81	43.87
43	4	89	0.32	7.04	75.00	7.10	45.16	26.13	38.06
44	4	116	0.17	37.73	51.49	8.13	43.28	25.70	35.15
45	4	124	0.31	6.02	60.43	6.48	40.10	23.29	33.62
46	4	169	0.19	6.72	75.00	6.72	65.80	36.26	59.08
47	4	182	0.06	7.75	75.00	7.75	63.69	35.72	55.94
48	4	214	0.23	7.60	57.03	7.56	44.93	26.24	37.37
49	4	221	0.23	21.36	62.38	7.96	44.89	26.42	36.93
50	4	230	0.19	8.43	48.34	8.37	46.62	27.50	38.25
51	4	252	0.30	7.34	75.00	7.34	45.13	26.23	37.79
52	4	312	0.11	8.31	75.00	6.30	43.38	24.84	37.08
53	4	329	0.75	5.96	57.81	5.94	29.65	17.79	23.71
54	4	405	0.24	6.67	75.00	6.29	34.92	20.60	28.63
55	4	436	0.76	4.57	34.69	4.50	29.60	17.05	25.10
56	4	461	0.08	7.93	69.88	7.93	31.32	19.62	23.39
57	4	479	0.51	7.51	42.07	7.51	30.53	19.02	23.02
58	4	514	0.29	1.68	58.86	3.36	38.91	21.14	35.55
59	4	558	0.72	5.54	47.44	5.54	25.37	15.45	19.83
60	4	633	0.49	3.96	73.37	3.96	49.22	26.59	45.26
61	4	673	0.20	5.84	75.00	5.84	63.22	34.53	57.37

62	4	704	0.29	5.29	64.12	5.29	46.29	25.79	41.00
63	4	715	0.11	7.74	75.00	7.74	48.47	28.11	40.73
64	4	742	0.15	43.21	74.51	6.29	48.01	27.15	41.72
65	4	754	0.32	5.43	60.57	5.24	45.47	25.36	40.22
66	4	789	0.23	4.31	75.00	4.19	60.39	32.29	56.19
67	4	811	0.15	5.70	75.00	5.70	52.16	28.93	46.46
68	4	833	0.07	5.16	75.00	5.07	63.12	34.10	58.05
69	4	871	0.25	4.65	75.00	4.65	43.53	24.09	38.87
70	4	912	0.60	-0.62	45.57	3.07	21.04	12.05	17.97
71	4	983	0.18	4.46	75.00	4.00	69.21	36.61	65.22
72	4	1020	0.44	5.51	50.98	5.42	31.77	18.59	26.35
73	4	1059	0.49	4.19	64.07	4.16	44.70	24.43	40.53
74	4	1111	0.12	6.63	75.00	6.55	46.52	26.54	39.96
75	4	1164	0.62	1.71	44.85	6.63	19.27	12.95	12.64
76	4	1241	0.31	7.10	75.00	7.10	48.63	27.87	41.52
77	4	1275	0.18	8.07	64.29	7.97	47.04	27.51	39.07
78	4	1301	0.46	6.41	56.88	6.57	40.30	23.43	33.73
79	4	1314	0.06	42.24	71.66	8.82	47.14	27.98	38.32
80	4	1354	0.20	7.21	75.00	7.21	46.71	26.96	39.50
81	4	1377	0.60	5.72	62.54	5.44	46.16	25.80	40.72
82	4	1403	0.06	39.15	74.75	8.46	46.61	27.53	38.15
83	4	1448	0.10	6.67	75.00	6.67	43.84	25.26	37.17
84	4	1459	0.66	5.32	49.92	5.32	43.61	24.47	38.29
85	4	1502	0.57	8.50	59.23	8.50	47.42	27.96	38.92
86	5	16	0.20	6.17	75.00	6.17	54.04	30.10	47.87
87	5	44	0.06	5.77	74.66	6.22	43.43	24.82	37.21
88	5	58	0.59	2.83	44.62	2.83	34.30	18.57	31.47
89	5	95	0.76	0.37	40.12	-2.19	26.35	12.08	28.54
90	5	146	0.54	3.39	42.53	3.41	30.35	16.88	26.94
91	5	173	0.74	2.83	35.83	2.81	29.68	16.24	26.87
92	5	216	0.26	5.13	57.90	5.07	45.07	25.07	40.01
93	5	232	0.33	4.90	62.40	3.93	52.51	28.22	48.58
94	5	252	0.13	6.52	74.61	6.52	47.92	27.22	41.40
95	5	282	0.43	5.34	48.48	7.47	34.88	21.18	27.42
96	5	317	0.73	4.05	54.59	4.03	31.49	17.76	27.45
97	5	354	0.72	6.69	38.21	6.69	31.71	19.20	25.02
98	5	406	0.78	5.02	48.01	4.98	32.51	18.75	27.53
99	5	441	0.39	4.70	62.19	5.47	40.70	23.08	35.23
100	5	501	0.53	6.50	50.53	6.50	43.05	24.78	36.55
101	5	518	0.07	8.64	75.00	8.59	47.79	28.19	39.20
102	5	547	0.60	7.43	52.84	7.43	49.65	28.54	42.23
103	5	560	0.18	35.29	57.10	9.40	47.66	28.53	38.26
104	6	25	0.43	6.98	61.58	6.98	52.34	29.66	45.36
105	6	36	0.26	6.10	52.90	6.21	52.67	29.44	46.46
106	6	66	0.29	5.24	69.23	5.24	49.42	27.33	44.18
107	6	112	0.27	4.70	51.95	4.70	38.51	21.60	33.81
108	6	119	0.34	6.04	46.16	6.02	31.56	18.79	25.54
109	6	138	0.26	3.95	59.55	3.95	39.16	21.56	35.21
110	6	165	0.34	3.22	53.41	3.22	31.52	17.37	28.30
111	6	199	0.83	-2.35	38.53	-2.35	25.93	11.79	28.28
112	7	20	0.71	4.97	51.35	4.97	44.44	24.70	39.47
113	7	62	0.52	4.60	54.16	4.60	41.64	23.12	37.04
114	7	109	0.45	5.36	57.61	5.50	44.25	24.88	38.75
115	7	120	0.12	8.08	75.00	7.69	45.39	26.54	37.70
116	7	163	0.68	3.75	47.45	5.45	35.21	20.33	29.76
117	7	206	0.63	5.28	55.27	5.28	45.53	25.40	40.25
118	7	286	0.75	7.03	48.81	7.03	30.86	18.95	23.83
119	7	320	0.11	8.20	75.00	8.20	51.20	29.70	43.00
120	7	330	0.07	9.09	57.38	9.09	51.09	30.09	42.00
121	7	350	0.48	6.53	52.10	6.63	44.19	25.41	37.57
122	7	389	0.50	4.64	44.56	5.70	36.16	20.93	30.46
123	7	422	0.56	7.13	42.15	7.13	32.60	19.87	25.47
124	7	486	0.75	5.74	42.09	5.74	29.48	17.61	23.74

125	7	509	0.23	6.88	65.62	7.27	38.00	22.64	30.73
126	7	540	0.17	6.84	75.00	6.74	47.35	27.05	40.61
127	7	562	0.22	5.28	58.11	5.28	45.00	25.14	39.72
128	7	571	0.45	4.86	48.44	4.12	42.48	23.30	38.36
129	7	582	0.24	6.04	75.00	6.97	38.61	22.79	31.64
130	7	622	0.05	6.24	75.00	6.24	60.33	33.28	54.09
131	7	630	0.17	6.20	75.00	6.20	54.02	30.11	47.83
132	7	660	0.39	5.24	43.42	5.32	32.58	18.95	27.25
133	7	718	0.58	4.73	52.11	3.45	38.66	21.05	35.21
134	7	777	0.79	3.45	36.83	3.45	30.30	16.87	26.85
135	7	833	0.77	1.49	46.68	0.53	28.27	14.40	27.74
136	7	889	0.70	-1.74	54.76	-1.74	30.07	14.16	31.80
137	7	925	0.09	6.66	75.00	6.60	46.12	26.36	39.53
138	7	936	0.06	6.82	75.00	6.82	55.71	31.27	48.89
139	7	980	0.39	5.09	50.94	4.99	45.64	25.31	40.66
140	7	998	0.24	5.38	70.22	5.38	46.51	25.95	41.13
141	7	1037	0.38	2.69	75.00	2.57	72.03	37.30	69.46
142	7	1060	0.18	7.22	64.48	7.22	53.89	30.55	46.67
143	7	1069	0.32	30.93	52.58	8.21	52.49	30.35	44.27
144	8	24	0.08	9.35	75.00	9.35	54.68	32.01	45.33
145	8	43	0.18	8.60	75.00	8.60	44.24	26.42	35.64
146	8	57	0.10	39.37	75.00	7.30	39.37	23.34	32.07
147	8	67	0.46	5.66	45.96	5.66	36.45	21.06	30.79
148	8	92	0.11	7.22	67.12	7.19	44.24	25.72	37.05
149	8	115	0.24	7.28	51.63	7.28	30.85	19.06	23.58
150	8	133	0.64	5.75	45.82	5.75	30.32	18.04	24.57
151	8	169	0.20	6.85	75.00	6.85	45.81	26.33	38.96
152	8	214	0.17	5.30	75.00	5.30	65.81	35.55	60.51
153	8	243	0.27	7.10	75.00	7.10	46.85	26.98	39.74
154	8	294	0.78	4.36	45.01	4.54	30.67	17.60	26.14
155	8	359	0.36	5.59	74.86	5.59	43.82	24.71	38.23
156	8	393	0.50	5.74	59.42	5.74	43.28	24.51	37.55
157	8	441	0.21	8.18	75.00	8.18	31.57	19.87	23.38
158	8	448	0.53	6.40	45.27	6.40	29.71	18.05	23.31
159	8	473	0.19	7.16	67.42	7.21	43.06	25.14	35.85
160	8	502	0.32	7.52	55.55	7.52	44.21	25.86	36.69
161	8	520	0.26	8.46	45.81	8.42	44.79	26.61	36.37
162	8	535	0.36	8.31	60.12	8.31	48.99	28.65	40.68
163	9	16	0.07	38.47	50.91	9.73	50.91	30.32	41.18
164	9	31	0.65	7.53	58.62	7.53	47.43	27.48	39.90
165	9	79	0.23	6.45	61.75	6.95	38.52	22.73	31.57
166	9	106	0.51	5.49	43.40	5.49	31.98	18.74	26.48
167	9	137	0.21	6.66	72.26	6.66	44.15	25.40	37.49
168	9	161	0.19	7.70	59.00	7.70	50.13	28.91	42.43
169	9	172	0.14	8.00	75.00	8.00	45.75	26.87	37.76
170	9	215	0.69	5.29	49.18	4.88	28.02	16.45	23.14
171	9	249	0.38	5.71	61.05	6.45	35.22	20.84	28.78
172	9	283	0.05	4.58	75.00	4.57	67.10	35.84	62.54
173	9	296	0.06	5.46	75.00	5.46	55.61	30.54	50.15
174	9	308	0.27	5.46	75.00	5.43	49.38	27.40	43.95
175	9	340	0.32	5.23	57.18	5.23	36.71	20.97	31.47
176	9	355	0.53	5.53	52.21	5.76	31.56	18.66	25.80
177	9	414	0.88	2.26	34.09	1.18	25.91	13.54	24.73
178	10	22	0.43	9.26	75.00	9.37	51.80	30.58	42.43
179	10	69	0.66	5.59	50.23	5.59	40.07	22.83	34.48
180	10	114	0.38	7.66	71.53	7.55	45.26	26.41	37.70
181	10	161	0.43	4.63	63.89	4.70	44.37	24.54	39.67
182	10	200	0.59	4.44	45.12	4.49	35.64	20.07	31.15
183	10	228	0.34	5.45	55.73	5.34	31.83	18.59	26.48
184	10	272	0.16	3.64	75.00	3.55	68.24	35.89	64.69
185	10	306	0.27	4.31	71.93	4.22	49.54	26.88	45.31
186	10	322	0.11	5.74	75.00	5.74	50.66	28.20	44.92
187	10	336	0.10	5.11	75.00	5.11	45.23	25.17	40.12

188	10	374	0.70	0.91	48.95	0.91	28.27	14.59	27.36
189	10	416	0.33	3.44	75.00	2.79	33.86	18.32	31.06
190	10	444	0.20	4.70	75.00	3.27	37.81	20.54	34.54
191	10	456	0.21	1.94	60.19	1.94	43.96	22.95	42.01
192	10	475	0.18	3.72	75.00	3.76	55.00	29.38	51.24
193	10	540	0.80	1.26	43.98	1.26	30.23	15.75	28.97
194	10	604	0.14	4.26	75.00	4.26	36.58	20.42	32.32
195	10	613	0.58	3.65	39.36	3.62	28.93	16.28	25.31
196	10	648	0.16	3.80	75.00	3.80	43.10	23.45	39.30
197	10	682	0.75	1.60	43.51	2.49	26.97	14.73	24.47
198	10	721	0.21	5.24	75.00	5.24	49.80	27.52	44.56
199	11	17	0.44	7.66	63.07	7.56	47.51	27.54	39.95
200	11	55	0.78	6.86	49.01	6.89	30.06	18.48	23.17
201	11	103	0.32	7.69	61.80	7.30	43.39	25.34	36.10
202	11	150	0.57	5.01	45.95	5.01	36.87	20.94	31.86
203	11	193	0.11	7.34	75.00	7.23	46.36	26.79	39.13
204	11	209	0.53	7.09	53.16	7.09	44.04	25.56	36.95
205	11	253	0.50	3.83	62.67	3.82	44.93	24.38	41.11
206	11	300	0.71	5.38	42.23	5.39	32.34	18.86	26.95
207	11	330	0.51	5.65	55.81	7.12	33.59	20.36	26.48
208	11	376	0.06	8.48	75.00	8.48	46.67	27.57	38.20
209	11	389	0.27	7.73	49.87	7.77	35.89	21.83	28.12
210	11	416	0.16	6.86	75.00	6.86	42.09	24.48	35.23
211	11	448	0.83	2.67	38.90	3.46	25.94	14.70	22.47
212	11	517	0.58	5.50	60.63	7.12	39.11	23.11	31.99
213	11	532	0.09	7.94	75.00	7.94	46.25	27.09	38.31
214	11	583	0.34	7.47	65.89	7.47	46.35	26.91	38.88
215	11	612	0.35	7.47	66.51	7.55	45.68	26.61	38.13
216	11	639	0.66	7.25	59.85	7.30	47.88	27.59	40.58
217	12	30	0.62	7.66	60.65	7.66	51.76	29.71	44.10
218	12	75	0.22	6.19	71.64	6.19	43.72	24.95	37.53
219	12	86	0.34	5.65	45.18	5.65	41.47	23.56	35.81
220	12	139	0.66	4.72	47.99	4.72	30.15	17.43	25.43
221	12	154	0.26	4.68	54.91	4.68	32.51	18.60	27.83
222	12	187	0.07	4.24	75.00	4.24	60.31	32.28	56.07
223	12	232	0.80	3.48	45.61	4.03	26.70	15.36	22.67
224	12	297	0.27	3.62	75.00	3.62	53.93	28.78	50.30
225	12	374	0.78	1.94	42.30	-0.19	23.89	11.85	24.08
226	12	418	0.37	4.49	55.88	4.49	36.86	20.68	32.36
227	12	437	0.07	4.99	67.47	4.82	45.99	25.40	41.18
228	12	451	0.37	4.68	70.11	4.66	45.63	25.14	40.96
229	12	512	0.78	7.11	56.47	7.11	29.86	18.49	22.76
230	12	590	0.15	4.05	75.00	4.05	30.09	17.07	26.05
231	12	600	0.41	2.11	33.71	2.11	28.96	15.54	26.85
232	12	607	0.41	3.66	53.98	4.81	30.40	17.61	25.59
233	12	654	0.32	6.45	59.03	6.45	43.70	25.08	37.25
234	12	687	0.26	4.99	75.00	5.09	55.77	30.43	50.68
235	12	745	0.78	4.15	44.98	4.15	28.46	16.31	24.31
236	12	797	0.30	6.70	75.00	6.91	44.02	25.46	37.12
237	12	816	0.33	6.03	74.35	6.11	46.53	26.32	40.42
238	12	859	0.12	5.35	75.00	10.82	78.14	44.48	67.32
239	12	869	0.07	6.30	75.00	6.21	72.31	39.26	66.10
240	12	877	0.05	41.21	75.00	8.76	59.61	34.18	50.85
241	12	890	0.23	8.40	75.00	8.40	51.56	29.98	43.16
242	12	934	0.46	7.18	49.42	6.95	43.86	25.41	36.91
243	12	945	0.18	7.40	75.00	7.36	44.66	26.01	37.29
244	12	975	0.05	41.92	75.00	8.41	54.59	31.50	46.18
245	12	1007	0.64	5.86	49.40	5.80	44.65	25.22	38.85
246	12	1021	0.17	7.14	75.00	7.14	44.63	25.88	37.50
247	12	1055	0.53	7.15	66.23	7.15	53.14	30.15	45.99
248	13	33	0.51	5.54	54.35	5.54	48.25	26.90	42.71
249	13	57	0.09	6.60	75.00	6.60	54.64	30.62	48.04
250	13	91	0.55	3.15	50.63	3.86	39.99	21.93	36.13

251	13	138	0.67	6.28	40.61	6.28	31.55	18.91	25.28
252	13	182	0.62	3.14	44.51	3.14	31.35	17.25	28.21
253	13	227	0.15	7.61	75.00	7.61	47.46	27.53	39.85
254	13	261	0.41	5.48	45.43	5.48	32.03	18.75	26.55
255	13	286	0.73	3.66	48.83	3.93	28.24	16.08	24.31
256	13	367	0.47	5.84	75.00	7.23	37.69	22.46	30.45
257	13	413	0.11	6.91	75.00	7.18	48.30	27.74	41.12
258	13	430	0.54	5.36	48.01	5.75	40.23	22.99	34.47
259	13	468	0.30	6.11	71.13	6.11	44.16	25.13	38.05
260	13	504	0.76	4.50	35.40	4.54	30.81	17.68	26.27
261	13	537	0.62	6.99	52.40	6.99	30.42	18.71	23.43
262	13	559	0.19	6.84	66.38	8.25	37.83	23.04	29.58
263	13	614	0.54	6.00	52.86	5.91	42.03	23.97	36.12
264	13	631	0.09	38.24	75.00	8.54	38.68	23.61	30.14
265	13	649	0.71	7.03	53.76	7.00	29.56	18.28	22.55
266	13	689	0.10	7.98	75.00	8.17	53.92	31.04	45.76
267	13	762	0.45	6.63	58.02	6.63	45.86	26.24	39.23
268	13	815	0.78	3.28	40.93	5.48	23.54	14.51	18.06
269	13	898	0.69	4.38	35.04	4.38	34.06	19.22	29.68
270	14	31	0.48	7.53	75.00	7.53	61.06	34.29	53.53
271	14	67	0.07	34.50	50.60	9.36	45.42	27.39	36.06
272	14	89	0.71	6.45	56.90	6.59	41.91	24.25	35.32
273	14	111	0.06	42.06	75.00	10.31	52.89	31.60	42.58
274	14	170	0.42	7.61	69.36	7.58	47.62	27.60	40.04
275	14	197	0.15	8.79	75.00	8.76	53.47	31.11	44.71
276	14	220	0.21	6.99	75.00	6.99	67.39	37.19	60.40
277	14	279	0.49	6.09	57.81	6.09	43.43	24.76	37.33
278	14	322	0.67	5.76	57.08	5.65	30.63	18.14	24.98
279	14	352	0.16	5.69	75.00	5.69	44.60	25.15	38.91
280	14	413	0.47	4.05	71.40	3.93	48.59	26.26	44.67
281	14	450	0.23	6.32	75.00	6.32	47.49	26.90	41.17
282	14	471	0.08	24.47	67.83	7.53	53.93	30.73	46.40
283	14	497	0.26	5.24	75.00	5.16	47.32	26.24	42.16
284	14	557	0.68	1.78	43.74	3.22	27.19	15.21	23.97
285	14	611	0.57	5.03	50.14	5.00	29.51	17.25	24.51
286	14	634	0.23	5.49	75.00	5.49	40.60	23.04	35.11
287	14	692	0.48	4.34	68.97	4.28	46.20	25.24	41.92
288	14	740	0.66	6.80	52.87	6.69	48.28	27.49	41.59
289	14	763	0.13	21.65	74.21	8.24	52.40	30.32	44.16
290	15	14	0.22	35.39	75.00	7.54	52.34	29.94	44.80
291	15	38	0.56	5.45	57.80	5.45	50.78	28.12	45.33
292	15	76	0.43	6.85	51.53	8.19	36.45	22.32	28.25
293	15	115	0.66	4.44	38.75	5.34	32.58	18.96	27.23
294	15	167	0.81	4.18	40.47	4.17	30.42	17.29	26.25
295	15	227	0.75	5.14	53.15	5.14	31.23	18.18	26.09
296	15	280	0.83	3.08	37.24	3.18	25.97	14.57	22.79
297	15	312	0.14	6.84	75.00	6.84	42.69	24.76	35.86
298	15	337	0.57	6.78	42.66	6.76	30.33	18.54	23.57
299	15	353	0.32	6.27	58.75	6.27	37.50	21.89	31.23
300	15	435	0.57	7.13	50.13	7.16	32.68	19.92	25.52
301	15	459	0.21	5.56	52.99	5.81	41.92	23.87	36.11
302	15	466	0.31	4.45	51.96	4.45	39.44	21.94	35.00
303	15	487	0.28	5.31	57.35	5.62	37.16	21.39	31.54
304	15	498	0.34	5.31	66.25	5.31	34.60	19.96	29.29
305	15	566	0.57	6.16	53.91	6.10	43.78	24.94	37.69
306	15	603	0.21	6.79	71.34	6.86	43.45	25.16	36.59
307	15	617	0.35	6.64	47.67	6.64	45.42	26.03	38.78
308	15	662	0.38	7.43	48.41	7.35	44.73	26.04	37.37
309	15	680	0.16	8.13	71.02	8.12	45.96	27.04	37.84
310	15	707	0.37	8.48	65.63	8.47	52.32	30.40	43.85
311	15	732	0.08	8.94	75.00	8.83	52.49	30.66	43.66
312	15	767	0.51	6.85	56.16	6.84	44.49	25.66	37.65
313	15	783	0.29	7.70	69.48	7.51	43.56	25.54	36.05

314	15	854	0.50	6.67	53.86	6.67	36.61	21.64	29.93
315	15	875	0.45	7.09	43.34	7.09	31.33	19.21	24.24
316	15	908	0.69	7.21	43.58	7.21	31.27	19.24	24.06
317	15	973	0.21	6.76	75.00	6.76	50.48	28.62	43.72
318	15	984	0.39	4.54	55.16	4.54	48.66	26.60	44.12
319	15	1010	0.18	7.12	73.08	7.12	49.64	28.38	42.51
320	15	1054	0.79	4.58	50.86	5.26	30.35	17.81	25.09
321	16	16	0.08	37.25	49.14	10.63	49.14	29.89	38.51
322	16	39	0.43	8.76	62.44	8.76	47.94	28.35	39.17
323	16	71	0.42	6.46	57.35	6.62	43.08	24.85	36.46
324	16	93	0.42	6.46	52.62	6.98	40.48	23.73	33.49
325	16	116	0.23	7.75	66.26	7.62	44.93	26.27	37.31
326	16	162	0.37	7.16	59.12	7.15	45.20	26.17	38.05
327	16	180	0.13	7.61	54.84	7.61	46.85	27.23	39.24
328	16	191	0.19	6.62	64.18	6.58	46.10	26.34	39.52
329	16	209	0.45	5.72	50.76	5.72	44.11	24.91	38.39
330	16	260	0.09	8.34	75.00	8.34	45.35	26.84	37.01
331	16	289	0.36	5.58	52.05	7.14	32.91	20.03	25.77
332	16	318	0.13	38.36	61.97	8.05	44.09	26.07	36.04
333	16	326	0.29	7.27	71.09	7.29	44.78	26.03	37.49
334	16	381	0.35	6.72	71.41	6.72	43.05	24.88	36.32
335	16	419	0.76	5.48	38.02	5.46	30.18	17.82	24.72
336	16	458	0.44	4.10	62.50	4.10	44.94	24.52	40.84
337	16	509	0.43	6.91	58.95	6.91	38.45	22.68	31.54
338	16	556	0.66	4.14	51.70	5.56	35.80	20.68	30.25
339	16	633	0.78	5.54	49.87	5.49	29.53	17.51	24.04
340	16	689	0.41	7.24	47.28	7.24	40.18	23.71	32.94
341	16	775	0.78	5.27	51.40	5.27	31.02	18.15	25.74
342	16	815	0.20	5.15	75.00	5.15	41.60	23.37	36.45
343	16	847	0.55	1.60	52.47	1.40	45.18	23.29	43.78
344	16	880	0.21	6.91	75.00	6.91	45.40	26.15	38.49
345	16	932	0.70	5.13	58.91	5.13	46.04	25.59	40.91

Cer_Rag

Index	Chr	Location (kb)	Fi	Ts	Te	T0	T100	T50	DT
1	1	14	0.89	-0.71	56.09	0.00	40.40	20.20	40.40
2	1	73	0.56	2.05	71.45	2.08	65.32	33.70	63.25
3	1	91	0.06	26.07	90.00	17.60	46.92	32.26	29.32
4	1	105	0.24	31.77	43.82	22.13	43.92	33.03	21.80
5	1	117	0.17	26.68	54.64	10.81	45.34	28.07	34.52
6	1	138	0.23	-1.29	59.98	-0.79	50.57	24.89	51.35
7	1	145	0.28	38.03	42.67	-1.17	43.65	21.24	44.83
8	1	176	0.54	-4.37	50.97	-4.22	36.39	16.09	40.61
9	1	223	0.10	43.70	62.06	23.11	61.51	42.31	38.40
10	2	0	0.18	23.13	90.00	7.32	85.64	46.48	78.32
11	2	27	0.09	20.16	90.00	7.51	68.22	37.86	60.71
12	2	39	0.07	11.81	90.00	4.74	65.15	34.94	60.41
13	2	47	0.06	54.99	62.23	6.72	56.38	31.55	49.66
14	2	62	0.63	-11.53	85.33	-11.73	65.08	26.67	76.81
15	2	90	0.13	34.42	61.09	6.47	60.00	33.24	53.53
16	2	100	0.13	38.75	67.48	8.29	58.69	33.49	50.40
17	2	171	0.42	-5.51	87.29	-5.51	63.93	29.21	69.44
18	2	199	0.16	-2.44	83.69	-0.75	37.22	18.23	37.98
19	2	207	0.25	-4.92	67.05	-5.00	42.01	18.50	47.01
20	2	233	0.50	-4.10	46.02	-3.76	33.63	14.94	37.39
21	2	257	0.19	-8.27	90.00	-8.45	49.77	20.66	58.22
22	2	325	0.53	6.53	90.00	-3.91	59.53	27.81	63.44
23	2	385	0.22	-0.31	90.00	0.83	56.48	28.66	55.65
24	2	397	0.13	4.31	55.72	4.41	47.61	26.01	43.20
25	2	409	0.75	5.86	50.09	5.91	37.16	21.53	31.25
26	2	494	0.65	3.53	69.39	2.86	62.22	32.54	59.35

27	2	505	0.11	19.29	75.98	14.73	50.69	32.71	35.96
28	2	517	0.18	23.47	90.00	10.51	62.73	36.62	52.23
29	2	532	0.07	54.74	61.13	21.36	60.99	41.17	39.62
30	2	592	0.05	54.80	90.00	10.16	62.76	36.46	52.60
31	2	622	0.71	8.40	55.57	0.00	47.26	23.63	47.26
32	2	640	0.05	34.75	76.53	12.61	40.58	26.60	27.96
33	2	699	0.07	63.95	90.00	11.76	65.03	38.40	53.27
34	2	714	0.43	6.10	86.22	6.12	65.56	35.84	59.44
35	2	742	0.36	3.66	90.00	3.61	71.14	37.38	67.53
36	2	775	0.66	3.76	75.66	4.03	62.27	33.15	58.24
37	2	808	0.26	26.36	77.92	16.36	67.05	41.71	50.69
38	3	37	0.66	-6.34	53.29	-6.31	35.13	14.41	41.43
39	3	72	0.56	-6.62	68.72	-6.70	37.19	15.24	43.89
40	3	84	0.12	-6.48	90.00	-6.48	47.77	20.65	54.24
41	3	118	0.26	-6.03	70.12	-6.09	49.78	21.85	55.87
42	3	128	0.23	-4.58	90.00	-4.74	48.83	22.05	53.57
43	3	146	0.05	40.27	46.44	4.44	46.46	25.45	42.01
44	3	164	0.40	-8.50	90.00	-8.62	67.42	29.40	76.04
45	3	185	0.09	34.99	45.70	7.51	45.75	26.63	38.24
46	3	202	0.35	-2.77	87.51	-5.30	45.95	20.33	51.25
47	3	225	0.62	-1.86	48.85	-1.65	33.96	16.16	35.61
48	3	279	0.44	3.76	79.84	5.99	57.23	31.61	51.24
49	3	299	0.09	6.83	90.00	10.34	83.91	47.13	73.57
50	3	311	0.06	52.76	74.72	12.25	75.56	43.91	63.31
51	4	27	0.24	29.10	90.00	16.69	54.77	35.73	38.08
52	4	41	0.46	-2.27	90.00	-3.12	76.03	36.46	79.16
53	4	93	0.31	4.29	90.00	4.28	63.43	33.85	59.15
54	4	126	0.56	5.72	62.33	5.59	48.69	27.14	43.09
55	4	199	0.06	64.06	90.00	10.36	64.63	37.49	54.27
56	4	212	0.39	7.61	90.00	6.87	59.53	33.20	52.66
57	4	234	0.59	4.95	75.14	4.98	63.69	34.33	58.71
58	4	328	0.74	1.61	64.00	-8.11	47.85	19.87	55.97
59	4	345	0.15	39.48	48.81	8.33	47.36	27.84	39.03
60	4	433	0.55	-1.42	49.12	-1.44	37.40	17.98	38.84
61	4	461	0.20	-1.27	74.45	0.03	43.93	21.98	43.90
62	4	489	0.36	-0.08	57.22	-1.00	44.57	21.79	45.56
63	4	509	0.17	-1.59	80.67	-1.75	44.11	21.18	45.86
64	4	542	0.19	-5.66	70.30	-7.14	49.86	21.36	57.00
65	4	555	0.70	5.47	52.79	-0.99	29.44	14.22	30.43
66	4	575	0.08	40.65	45.56	6.98	42.32	24.65	35.33
67	4	634	0.57	3.84	84.53	-3.89	68.84	32.47	72.74
68	4	672	0.07	54.08	60.18	28.43	60.55	44.49	32.12
69	4	701	0.51	4.74	90.00	0.41	64.25	32.33	63.84
70	4	733	0.06	29.95	90.00	8.43	58.09	33.26	49.66
71	4	748	0.41	0.64	79.46	0.40	68.18	34.29	67.78
72	4	788	0.12	53.67	72.11	2.16	73.16	37.66	71.00
73	4	805	0.26	16.42	90.00	5.88	69.42	37.65	63.54
74	4	818	0.09	54.54	60.65	10.83	60.77	35.80	49.93
75	4	881	0.36	-13.89	90.00	-13.58	56.87	21.65	70.45
76	4	900	0.14	40.26	90.00	-4.75	43.52	19.38	48.27
77	4	912	0.64	2.25	50.51	-3.91	27.65	11.87	31.55
78	4	1007	0.05	39.38	68.80	7.19	47.76	27.48	40.56
79	4	1022	0.68	2.30	66.27	-1.47	47.78	23.15	49.25
80	4	1061	0.46	-3.61	90.00	-3.70	65.60	30.95	69.31
81	4	1159	0.76	1.04	46.27	1.24	22.51	11.87	21.27
82	4	1245	0.47	6.04	90.00	5.64	64.48	35.06	58.84
83	4	1260	0.05	54.61	60.45	19.48	60.49	39.98	41.01
84	4	1300	0.62	2.62	89.72	1.26	63.04	32.15	61.78
85	4	1359	0.27	5.80	90.00	5.79	68.56	37.17	62.77
86	4	1382	0.49	7.59	79.81	6.27	53.44	29.86	47.17
87	4	1400	0.07	63.08	90.00	8.16	64.40	36.28	56.23
88	4	1459	0.71	-1.41	63.43	-1.93	62.91	30.49	64.84
89	4	1507	0.18	11.66	85.03	11.55	68.18	39.87	56.63

90	4	1524	0.40	5.71	87.32	6.17	68.61	37.39	62.44
91	5	59	0.59	1.39	49.46	1.39	34.45	17.92	33.06
92	5	94	0.58	21.21	35.19	-5.14	30.85	12.86	35.98
93	5	149	0.50	-3.61	56.21	-3.61	35.14	15.76	38.75
94	5	175	0.62	2.66	45.24	2.39	33.00	17.69	30.61
95	5	227	0.42	4.23	79.15	4.41	61.99	33.20	57.59
96	5	281	0.41	4.31	75.87	4.23	49.00	26.62	44.77
97	5	316	0.63	-3.73	57.92	-3.78	39.15	17.68	42.93
98	5	355	0.62	6.76	46.02	6.28	35.42	20.85	29.14
99	5	408	0.62	6.30	45.79	6.63	36.89	21.76	30.26
100	5	424	0.21	18.24	43.67	11.73	35.06	23.39	23.33
101	5	438	0.38	6.16	72.31	6.74	36.07	21.41	29.32
102	5	501	0.59	8.45	86.08	7.74	48.36	28.05	40.62
103	5	518	0.28	7.06	90.00	7.06	52.54	29.80	45.48
104	5	556	0.52	0.00	90.00	0.00	81.43	40.71	81.43
105	6	0	0.06	58.60	77.46	17.87	75.42	46.65	57.55
106	6	18	0.52	13.77	90.00	7.48	74.85	41.16	67.37
107	6	45	0.27	3.88	90.00	3.85	72.76	38.30	68.91
108	6	63	0.23	5.75	89.75	6.16	62.84	34.50	56.68
109	6	118	0.61	-0.45	63.68	-0.45	47.97	23.76	48.42
110	6	166	0.39	-6.59	70.76	-5.86	45.59	19.87	51.45
111	6	180	0.06	44.13	50.88	-2.64	50.50	23.93	53.14
112	6	189	0.05	44.81	52.69	-7.24	49.07	20.92	56.31
113	6	202	0.65	-4.50	59.45	-4.62	34.75	15.07	39.37
114	6	270	0.20	42.22	64.96	21.48	62.96	42.22	41.48
115	7	16	0.67	-0.34	70.48	0.28	60.32	30.30	60.04
116	7	38	0.06	55.06	56.58	17.32	55.28	36.30	37.96
117	7	64	0.59	3.41	90.00	1.23	53.87	27.55	52.65
118	7	113	0.60	9.04	90.00	8.37	48.55	28.46	40.18
119	7	163	0.65	-6.90	90.00	-9.16	60.39	25.61	69.55
120	7	187	0.08	46.95	49.02	16.40	48.95	32.68	32.55
121	7	206	0.52	2.50	78.41	0.00	60.00	30.00	60.00
122	7	289	0.83	3.78	65.20	-1.86	49.40	23.77	51.26
123	7	353	0.58	7.54	81.25	7.29	53.75	30.52	46.46
124	7	395	0.43	0.72	77.30	-5.93	58.73	26.40	64.66
125	7	419	0.65	4.30	67.31	0.54	47.94	24.24	47.40
126	7	490	0.80	2.55	50.23	-0.61	34.53	16.96	35.13
127	7	500	0.08	17.96	90.00	7.47	36.57	22.02	29.11
128	7	572	0.76	3.18	59.40	-0.38	39.34	19.48	39.72
129	7	660	0.74	11.96	76.74	-7.32	45.38	19.03	52.70
130	7	717	0.73	5.87	63.61	0.23	48.43	24.33	48.20
131	7	779	0.81	8.86	46.82	4.12	29.98	17.05	25.87
132	7	833	0.83	2.86	42.09	0.00	30.91	15.46	30.91
133	7	865	0.09	24.49	54.18	-5.01	54.52	24.75	59.53
134	7	888	0.77	-5.93	50.53	-6.13	35.12	14.49	41.26
135	7	900	0.16	20.12	90.00	-4.78	47.35	21.28	52.13
136	7	954	0.11	44.98	57.17	24.19	57.68	40.94	33.49
137	7	982	0.34	14.17	80.80	3.09	60.80	31.95	57.72
138	7	988	0.43	-1.77	86.76	-1.60	69.04	33.72	70.64
139	7	1000	0.29	19.30	90.00	4.92	60.00	32.46	55.08
140	7	1010	0.15	41.85	59.42	11.39	58.73	35.06	47.34
141	7	1067	0.72	16.46	71.98	16.46	71.23	43.85	54.78
142	7	1081	0.17	22.05	90.00	17.52	73.90	45.71	56.38
143	8	2	0.13	44.24	72.92	25.80	67.67	46.74	41.86
144	8	13	0.10	44.10	68.41	24.87	64.75	44.81	39.88
145	8	60	0.44	14.31	84.64	-6.19	55.25	24.53	61.45
146	8	72	0.49	-14.27	90.00	-14.91	61.58	23.33	76.49
147	8	131	0.41	2.52	57.25	2.78	33.68	18.23	30.90
148	8	143	0.20	-1.25	63.79	0.93	42.91	21.92	41.98
149	8	166	0.14	6.91	90.00	6.91	50.88	28.90	43.98
150	8	216	0.09	52.88	60.81	26.04	60.91	43.48	34.87
151	8	242	0.42	11.35	90.00	6.62	65.97	36.30	59.35
152	8	261	0.07	50.42	60.05	18.06	52.88	35.47	34.82

153	8	284	0.12	-3.05	90.00	0.58	56.17	28.38	55.60
154	8	295	0.72	7.55	67.06	-8.76	51.39	21.31	60.15
155	8	364	0.38	7.74	90.00	4.97	56.74	30.86	51.77
156	8	388	0.45	3.36	83.21	3.39	57.66	30.53	54.28
157	8	450	0.74	8.89	72.27	-1.06	43.73	21.33	44.79
158	8	512	0.41	8.25	90.00	8.51	52.10	30.31	43.60
159	8	543	0.11	16.07	90.00	18.99	76.29	47.64	57.29
160	9	3	0.78	-1.24	79.62	-1.24	70.68	34.72	71.92
161	9	32	0.05	57.19	62.15	14.00	62.74	38.37	48.74
162	9	75	0.35	15.16	90.00	5.20	48.42	26.81	43.21
163	9	84	0.07	48.17	49.30	7.71	48.20	27.96	40.49
164	9	104	0.60	7.18	66.74	-5.14	50.53	22.69	55.67
165	9	139	0.22	7.90	90.00	7.01	63.16	35.09	56.15
166	9	180	0.06	19.94	90.00	12.10	55.59	33.84	43.50
167	9	213	0.63	-1.38	56.05	-1.38	39.98	19.30	41.36
168	9	243	0.34	3.19	77.08	3.28	47.39	25.34	44.10
169	9	256	0.07	50.29	90.00	8.56	49.96	29.26	41.40
170	9	324	0.15	9.71	90.00	9.85	52.03	30.94	42.18
171	9	339	0.25	2.66	75.56	3.15	52.72	27.93	49.57
172	9	349	0.44	-0.81	70.88	-1.86	52.93	25.53	54.79
173	9	415	0.81	-1.13	54.17	-3.21	37.87	17.33	41.07
174	10	0	0.58	3.79	90.00	3.79	90.43	47.11	86.64
175	10	68	0.69	4.94	71.61	3.07	47.39	25.23	44.32
176	10	107	0.16	7.44	90.00	7.39	53.52	30.46	46.13
177	10	113	0.22	4.34	90.00	5.11	56.54	30.83	51.42
178	10	145	0.07	48.37	53.67	16.07	52.34	34.20	36.27
179	10	162	0.46	-2.43	82.72	-2.54	54.98	26.22	57.53
180	10	201	0.35	1.89	65.11	1.90	39.32	20.61	37.42
181	10	213	0.11	3.34	65.19	4.12	38.69	21.41	34.57
182	10	223	0.46	-3.59	68.22	-2.54	54.99	26.22	57.53
183	10	273	0.11	53.32	60.00	23.19	60.00	41.59	36.81
184	10	305	0.30	16.36	90.00	-8.96	80.38	35.71	89.35
185	10	321	0.09	12.22	90.00	5.88	69.71	37.80	63.83
186	10	376	0.68	-15.28	79.92	-18.80	48.08	14.64	66.89
187	10	425	0.34	-1.63	66.15	-2.03	35.61	16.79	37.64
188	10	443	0.39	1.49	77.45	1.60	44.25	22.93	42.65
189	10	538	0.74	-3.76	52.91	-3.76	38.77	17.51	42.53
190	10	600	0.05	38.23	44.90	7.31	42.71	25.01	35.39
191	10	614	0.76	-3.04	56.12	-3.27	38.08	17.40	41.35
192	10	684	0.78	9.75	51.80	-6.21	36.56	15.17	42.77
193	10	743	0.37	29.68	60.31	18.34	50.52	34.43	32.17
194	11	3	0.28	4.35	90.00	4.17	71.23	37.70	67.06
195	11	12	0.13	5.48	90.00	5.75	67.32	36.53	61.57
196	11	55	0.76	-0.44	58.38	-0.74	32.97	16.11	33.71
197	11	98	0.49	-2.76	82.03	-3.18	48.07	22.45	51.25
198	11	136	0.10	38.69	48.85	5.70	48.82	27.26	43.12
199	11	153	0.67	-8.03	62.96	-8.15	47.27	19.56	55.41
200	11	208	0.62	1.96	58.98	2.04	45.78	23.91	43.74
201	11	252	0.43	4.73	72.85	-1.98	51.01	24.52	52.99
202	11	302	0.72	-8.22	55.20	-8.63	41.86	16.62	50.49
203	11	321	0.38	14.99	55.20	5.19	37.33	21.26	32.14
204	11	396	0.46	11.22	59.16	5.72	43.00	24.36	37.28
205	11	417	0.35	-6.03	68.63	-5.83	44.00	19.09	49.83
206	11	448	0.78	2.38	39.69	-5.03	28.81	11.89	33.84
207	11	520	0.53	0.19	69.15	0.00	48.95	24.48	48.95
208	11	593	0.15	15.59	90.00	6.47	56.41	31.44	49.94
209	11	617	0.64	-6.32	84.38	-0.07	55.51	27.72	55.58
210	11	655	0.19	37.29	60.50	23.32	60.68	42.00	37.36
211	11	661	0.19	33.01	76.97	31.90	54.07	42.98	22.17
212	12	12	0.24	4.19	90.00	4.73	70.46	37.59	65.73
213	12	26	0.34	2.37	90.00	2.57	74.99	38.78	72.43
214	12	67	0.07	55.83	69.05	5.97	55.29	30.63	49.31
215	12	83	0.54	1.80	70.25	4.33	50.78	27.55	46.46

216	12	144	0.42	12.32	53.90	-5.12	45.00	19.94	50.12
217	12	234	0.72	-0.39	45.40	-0.59	36.62	18.02	37.21
218	12	293	0.17	19.76	90.00	4.14	66.00	35.07	61.86
219	12	371	0.66	-4.69	45.97	-5.59	34.67	14.54	40.26
220	12	388	0.10	44.78	58.46	-4.98	47.86	21.44	52.84
221	12	411	0.45	-6.21	70.24	-6.30	46.03	19.86	52.34
222	12	466	0.24	3.75	90.00	3.76	66.55	35.15	62.79
223	12	513	0.82	4.04	56.36	-0.05	37.53	18.74	37.58
224	12	607	0.76	6.92	49.84	-6.67	38.87	16.10	45.54
225	12	663	0.35	7.40	65.07	7.42	47.65	27.54	40.24
226	12	679	0.12	44.52	59.66	6.87	56.81	31.84	49.94
227	12	745	0.79	11.02	47.38	-5.06	34.88	14.91	39.94
228	12	799	0.41	8.33	80.79	6.94	50.32	28.63	43.38
229	12	817	0.35	8.02	90.00	8.12	50.97	29.55	42.85
230	12	889	0.42	9.62	90.00	8.99	63.12	36.05	54.12
231	12	932	0.73	6.88	67.88	6.50	50.52	28.51	44.02
232	12	1011	0.68	2.79	81.21	-1.10	59.62	29.26	60.73
233	12	1062	0.50	5.47	90.00	5.13	69.67	37.40	64.54
234	13	32	0.24	13.14	90.00	7.06	52.10	29.58	45.05
235	13	45	0.40	0.17	90.00	-1.79	63.90	31.06	65.68
236	13	92	0.39	-4.13	85.46	-4.17	47.34	21.59	51.51
237	13	101	0.12	21.38	61.43	3.29	47.05	25.17	43.75
238	13	139	0.50	1.26	54.90	-1.51	37.72	18.10	39.23
239	13	180	0.76	-1.48	40.00	-8.96	31.61	11.33	40.57
240	13	262	0.29	-4.29	90.00	-4.34	39.53	17.60	43.88
241	13	280	0.56	13.04	39.44	2.53	34.46	18.50	31.93
242	13	289	0.26	-6.52	90.00	-6.37	34.19	13.91	40.56
243	13	371	0.65	6.52	66.53	2.60	45.33	23.96	42.73
244	13	431	0.68	1.76	66.20	0.14	49.59	24.86	49.46
245	13	473	0.06	5.10	64.52	4.91	49.24	27.08	44.32
246	13	504	0.70	-2.18	41.67	-2.25	37.85	17.80	40.10
247	13	516	0.13	18.19	80.30	4.99	35.45	20.22	30.46
248	13	528	0.13	29.44	33.32	7.46	33.27	20.36	25.81
249	13	539	0.54	3.59	44.89	3.47	36.39	19.93	32.92
250	13	550	0.08	5.70	90.00	5.76	38.28	22.02	32.52
251	13	620	0.43	-4.45	80.08	-3.88	51.04	23.58	54.92
252	13	649	0.58	4.49	52.54	4.64	34.95	19.80	30.30
253	13	766	0.39	-3.95	79.15	-4.03	52.88	24.43	56.91
254	13	816	0.78	4.48	39.22	-0.96	28.16	13.60	29.11
255	13	888	0.07	29.80	38.93	6.68	36.50	21.59	29.81
256	13	897	0.88	3.76	42.89	3.85	31.26	17.56	27.41
257	14	1	0.09	24.10	90.00	17.74	63.81	40.77	46.07
258	14	16	0.31	7.53	90.00	7.67	72.34	40.00	64.67
259	14	36	0.10	6.18	76.28	7.27	60.41	33.84	53.14
260	14	63	0.40	5.08	70.25	5.11	47.08	26.09	41.97
261	14	95	0.38	7.17	41.93	6.54	37.20	21.87	30.66
262	14	154	0.07	40.02	45.03	16.67	44.97	30.82	28.29
263	14	167	0.47	0.73	77.40	2.57	64.52	33.54	61.94
264	14	197	0.21	20.80	78.14	14.72	45.32	30.02	30.60
265	14	219	0.09	7.55	90.00	7.54	66.20	36.87	58.66
266	14	253	0.24	1.46	87.03	2.57	59.73	31.15	57.16
267	14	277	0.41	8.38	59.42	8.38	36.51	22.44	28.13
268	14	322	0.81	5.17	32.57	5.16	28.40	16.78	23.23
269	14	356	0.29	4.64	81.83	4.78	40.74	22.76	35.96
270	14	412	0.42	4.97	72.50	5.02	47.07	26.04	42.05
271	14	449	0.33	-1.64	89.71	1.11	55.09	28.10	53.98
272	14	489	0.14	-1.52	90.00	1.53	61.26	31.39	59.73
273	14	497	0.16	1.73	89.38	0.94	45.58	23.26	44.64
274	14	547	0.47	1.52	37.70	1.53	27.04	14.29	25.50
275	14	560	0.55	7.76	25.58	7.68	15.68	11.68	7.99
276	14	610	0.60	2.96	32.92	2.61	30.21	16.41	27.60
277	14	625	0.13	23.26	39.42	7.70	33.73	20.72	26.03
278	14	634	0.12	0.73	90.00	2.07	37.08	19.57	35.00

279	14	685	0.06	5.84	84.80	6.21	39.97	23.09	33.76
280	14	696	0.65	1.51	52.22	0.73	40.59	20.66	39.86
281	14	716	0.05	5.78	50.15	5.68	47.10	26.39	41.41
282	14	736	0.44	5.80	58.34	5.79	42.94	24.36	37.15
283	14	753	0.11	6.35	58.44	8.34	48.10	28.22	39.75
284	14	772	0.26	4.66	90.00	4.92	62.43	33.67	57.51
285	15	19	0.13	11.99	90.00	6.95	59.49	33.22	52.54
286	15	42	0.42	1.37	72.93	1.47	50.62	26.04	49.15
287	15	82	0.42	6.11	47.38	6.10	33.61	19.86	27.50
288	15	100	0.06	29.70	31.55	9.39	31.36	20.38	21.97
289	15	111	0.77	5.54	35.21	5.71	27.18	16.44	21.47
290	15	165	0.72	10.55	36.45	0.02	27.65	13.83	27.64
291	15	229	0.70	4.31	37.67	3.39	27.52	15.45	24.13
292	15	278	0.78	0.79	38.44	-3.10	25.20	11.05	28.30
293	15	332	0.14	29.19	33.09	5.86	33.00	19.43	27.14
294	15	342	0.73	2.10	49.43	-2.82	35.44	16.31	38.26
295	15	439	0.68	7.36	38.53	7.32	29.59	18.46	22.27
296	15	468	0.64	5.78	34.91	5.69	29.75	17.72	24.06
297	15	491	0.45	6.33	40.58	6.31	30.63	18.47	24.32
298	15	500	0.17	7.92	90.00	8.29	31.55	19.92	23.27
299	15	569	0.64	8.27	43.53	8.08	36.35	22.22	28.27
300	15	603	0.48	7.60	47.00	7.46	34.78	21.12	27.32
301	15	626	0.10	6.09	53.21	5.95	44.48	25.21	38.54
302	15	651	0.46	-3.75	66.89	-3.80	52.51	24.35	56.31
303	15	674	0.32	14.87	55.13	7.62	43.16	25.39	35.54
304	15	700	0.07	49.05	90.00	8.58	49.04	28.81	40.46
305	15	726	0.10	7.47	90.00	7.76	66.27	37.02	58.51
306	15	734	0.10	11.30	90.00	11.27	50.20	30.74	38.93
307	15	765	0.14	9.17	72.05	9.42	44.14	26.78	34.72
308	15	778	0.54	8.39	50.46	8.44	40.35	24.39	31.91
309	15	853	0.44	5.88	54.94	5.85	30.77	18.31	24.92
310	15	874	0.61	6.74	39.80	6.67	29.89	18.28	23.21
311	15	905	0.53	4.59	46.00	4.69	35.86	20.28	31.17
312	15	988	0.58	3.74	68.29	3.46	48.40	25.93	44.93
313	15	1011	0.17	5.29	90.00	5.23	46.66	25.94	41.44
314	15	1055	0.59	6.86	45.45	4.84	27.76	16.30	22.91
315	16	4	0.64	22.97	40.15	22.91	34.09	28.50	11.17
316	16	33	0.31	4.61	88.71	4.73	69.26	37.00	64.53
317	16	69	0.67	7.45	38.86	7.20	35.40	21.30	28.20
318	16	94	0.51	8.25	47.86	8.31	34.66	21.49	26.35
319	16	117	0.32	4.96	72.59	5.10	47.72	26.41	42.62
320	16	129	0.06	5.68	83.85	6.20	63.21	34.71	57.01
321	16	157	0.05	6.11	90.00	6.11	46.32	26.22	40.21
322	16	167	0.13	18.86	69.59	6.43	47.74	27.08	41.31
323	16	185	0.39	-3.49	53.05	-3.54	47.90	22.18	51.44
324	16	211	0.52	10.04	48.63	6.40	36.88	21.64	30.49
325	16	253	0.14	8.94	90.00	8.82	45.95	27.39	37.13
326	16	283	0.70	0.67	42.90	-1.54	35.52	16.99	37.06
327	16	306	0.14	27.59	35.20	12.46	35.27	23.86	22.82
328	16	324	0.54	4.76	56.15	4.91	41.70	23.30	36.79
329	16	375	0.06	9.66	59.24	9.85	37.73	23.79	27.88
330	16	385	0.49	7.26	45.55	7.34	33.00	20.17	25.66
331	16	420	0.64	5.30	25.57	5.22	22.75	13.98	17.53
332	16	456	0.47	-5.41	59.47	-5.08	43.12	19.02	48.21
333	16	513	0.71	8.09	41.15	7.82	33.44	20.63	25.62
334	16	559	0.65	4.98	40.27	5.06	35.41	20.24	30.35
335	16	571	0.26	10.07	53.04	10.00	35.18	22.59	25.18
336	16	632	0.87	5.00	34.36	4.92	27.45	16.19	22.53
337	16	689	0.78	7.57	51.41	7.56	35.74	21.65	28.18
338	16	777	0.82	8.90	33.21	0.68	24.23	12.46	23.55
339	16	820	0.51	8.00	40.57	7.72	27.71	17.71	19.99
340	16	846	0.41	-6.55	68.88	-11.66	49.05	18.69	60.72
341	16	864	0.06	20.84	90.00	10.00	49.54	29.77	39.54

342	16	872	0.07	6.96	90.00	7.29	48.52	27.90	41.24
343	16	940	0.89	-4.75	61.03	-8.27	46.24	18.99	54.50

Pom_Hei

Index	Chr	Location (kb)	Fi	Ts	Te	T0	T100	T50	DT
1	1	11	0.64	56.16	131.85	58.18	113.83	86.00	55.65
2	1	53	0.54	49.73	114.46	54.68	107.32	81.00	52.64
3	1	97	0.27	51.04	121.55	54.72	105.27	79.99	50.55
4	1	111	0.34	54.57	111.71	49.43	104.93	77.18	55.50
5	1	126	0.14	48.77	124.86	52.56	106.73	79.64	54.17
6	1	138	0.28	56.86	140.42	56.90	99.24	78.07	42.34
7	1	157	0.22	109.48	131.88	60.41	108.17	84.29	47.75
8	1	180	0.18	46.34	172.18	56.78	104.02	80.40	47.24
9	1	201	0.76	53.13	109.13	51.51	97.29	74.40	45.79
10	1	245	0.62	55.97	117.68	57.34	107.81	82.57	50.47
11	1	283	0.41	55.32	126.16	59.88	103.69	81.78	43.81
12	1	325	0.58	51.07	117.43	49.23	103.00	76.11	53.77
13	1	368	0.66	46.02	110.56	48.57	102.93	75.75	54.35
14	1	397	0.24	56.58	153.82	58.94	98.44	78.69	39.51
15	1	432	0.36	71.71	114.62	57.37	102.21	79.79	44.84
16	1	452	0.16	92.74	99.58	56.28	99.27	77.77	42.99
17	1	467	0.16	50.45	114.28	54.65	101.90	78.27	47.25
18	1	488	0.30	44.60	111.87	51.38	106.28	78.83	54.90
19	1	533	0.24	51.75	155.37	62.95	105.08	84.01	42.13
20	1	565	0.69	45.16	109.59	49.45	98.71	74.08	49.26
21	1	578	0.16	66.02	149.51	54.18	103.84	79.01	49.65
22	1	605	0.65	49.78	112.05	55.82	97.48	76.65	41.66
23	1	618	0.15	54.01	137.14	55.09	105.46	80.27	50.38
24	1	634	0.10	97.31	125.98	60.00	102.61	81.30	42.61
25	1	649	0.54	53.22	114.00	56.49	99.59	78.04	43.10
26	1	668	0.13	93.81	119.45	58.90	103.52	81.21	44.62
27	1	676	0.29	48.51	134.79	59.53	102.26	80.89	42.73
28	1	691	0.13	95.65	122.77	57.74	100.07	78.90	42.33
29	1	714	0.57	45.40	110.84	48.38	102.64	75.51	54.26
30	1	739	0.13	92.46	122.71	57.23	101.48	79.36	44.24
31	1	757	0.37	49.37	122.97	54.98	105.34	80.16	50.36
32	1	784	0.35	54.56	128.00	56.97	100.35	78.66	43.37
33	1	801	0.34	55.02	126.44	57.14	98.84	77.99	41.70
34	1	808	0.15	54.06	149.25	56.23	100.83	78.53	44.59
35	1	840	0.75	46.98	105.88	49.13	101.45	75.29	52.32
36	1	856	0.08	98.25	99.75	51.34	102.52	76.93	51.18
37	1	882	0.49	41.47	118.72	49.03	103.89	76.46	54.86
38	1	913	0.59	69.21	108.23	47.51	98.97	73.24	51.46
39	1	928	0.08	96.49	102.29	59.06	101.09	80.07	42.03
40	1	962	0.62	48.98	113.45	49.77	98.31	74.04	48.54
41	1	986	0.37	48.95	128.47	54.18	100.25	77.21	46.07
42	1	1022	0.47	49.30	123.92	50.59	104.19	77.39	53.60
43	1	1036	0.19	91.93	110.27	55.60	100.29	77.94	44.69
44	1	1043	0.35	48.63	122.83	54.43	101.46	77.94	47.02
45	1	1063	0.26	70.81	122.09	54.07	99.54	76.81	45.47
46	1	1069	0.27	45.02	124.97	55.13	96.95	76.04	41.82
47	1	1084	0.27	51.13	114.68	42.32	99.46	70.89	57.14
48	1	1096	0.56	51.62	100.10	40.92	94.62	67.77	53.70
49	1	1112	0.26	56.97	126.52	55.01	97.53	76.27	42.52
50	1	1137	0.59	52.20	108.97	54.93	94.64	74.78	39.71
51	1	1149	0.14	47.19	121.10	55.63	97.11	76.37	41.47
52	1	1161	0.47	49.51	118.94	53.27	100.23	76.75	46.95
53	1	1184	0.32	48.67	130.49	48.57	98.32	73.44	49.75
54	1	1199	0.56	50.39	109.83	39.22	101.06	70.14	61.84
55	1	1226	0.39	50.66	125.41	52.16	100.65	76.40	48.49

56	1	1258	0.52	47.33	107.90	45.33	101.19	73.26	55.86
57	1	1282	0.44	46.00	115.11	47.55	100.89	74.22	53.33
58	1	1313	0.18	48.95	130.62	55.60	99.72	77.66	44.12
59	1	1326	0.38	59.04	108.80	57.58	97.25	77.42	39.67
60	1	1340	0.32	73.21	122.89	58.38	99.08	78.73	40.70
61	1	1374	0.10	47.90	149.52	54.81	101.24	78.02	46.43
62	1	1387	0.63	48.28	108.62	50.75	94.23	72.49	43.47
63	1	1419	0.40	45.40	136.03	52.67	100.35	76.51	47.68
64	1	1447	0.61	41.17	111.47	46.81	96.57	71.69	49.76
65	1	1471	0.43	63.82	106.71	44.49	97.22	70.85	52.73
66	1	1489	0.25	40.27	125.81	49.63	96.68	73.15	47.05
67	1	1514	0.42	42.74	110.52	46.74	97.95	72.34	51.21
68	1	1534	0.10	97.97	100.17	57.61	99.92	78.77	42.30
69	1	1551	0.38	59.00	114.36	57.34	96.07	76.70	38.74
70	1	1570	0.40	59.39	106.18	51.19	95.14	73.16	43.95
71	1	1582	0.47	48.39	105.55	50.40	97.55	73.98	47.15
72	1	1617	0.28	52.73	129.35	55.79	103.36	79.58	47.57
73	1	1623	0.31	47.61	112.79	57.78	102.50	80.14	44.72
74	1	1635	0.34	87.57	112.60	57.81	97.11	77.46	39.30
75	1	1658	0.50	50.66	121.32	53.98	100.94	77.46	46.96
76	1	1670	0.23	49.99	138.21	55.63	101.76	78.70	46.13
77	1	1681	0.14	80.23	132.32	58.15	100.51	79.33	42.36
78	1	1690	0.23	48.74	137.43	54.74	100.94	77.84	46.20
79	1	1705	0.21	92.94	113.64	46.66	97.18	71.92	50.52
80	1	1716	0.32	66.72	112.18	44.43	93.87	69.15	49.45
81	1	1729	0.37	84.67	103.75	44.72	97.45	71.09	52.73
82	1	1753	0.24	74.96	120.55	53.03	97.31	75.17	44.27
83	1	1774	0.50	59.87	113.67	54.02	98.48	76.25	44.47
84	1	1807	0.47	63.44	119.70	51.80	98.30	75.05	46.49
85	1	1832	0.52	45.64	109.07	46.07	99.83	72.95	53.76
86	1	1869	0.66	57.16	105.40	52.82	95.95	74.38	43.14
87	1	1881	0.09	95.41	98.35	58.87	95.89	77.38	37.03
88	1	1903	0.33	53.10	120.54	55.35	102.32	78.83	46.97
89	1	1918	0.16	94.31	97.14	57.89	101.68	79.78	43.79
90	1	1936	0.32	63.50	117.78	56.86	97.79	77.33	40.93
91	1	1946	0.14	47.07	165.58	56.42	103.23	79.82	46.81
92	1	1982	0.75	43.25	108.23	48.30	99.84	74.07	51.54
93	1	2024	0.52	77.52	115.57	53.18	92.55	72.87	39.37
94	1	2043	0.30	44.01	127.16	53.31	104.13	78.72	50.81
95	1	2064	0.15	45.69	137.18	54.69	102.35	78.52	47.65
96	1	2077	0.26	46.72	116.93	52.56	102.63	77.59	50.07
97	1	2086	0.35	74.52	114.04	51.96	97.52	74.74	45.56
98	1	2097	0.41	58.45	122.76	54.59	99.88	77.24	45.29
99	1	2113	0.07	94.57	107.91	58.95	103.81	81.38	44.86
100	1	2121	0.06	52.81	174.05	59.90	101.03	80.47	41.14
101	1	2135	0.55	58.75	110.68	51.83	99.26	75.54	47.43
102	1	2154	0.27	52.31	140.22	56.32	97.98	77.15	41.66
103	1	2182	0.28	65.47	121.90	52.16	97.93	75.04	45.77
104	1	2192	0.45	71.52	104.15	43.80	97.26	70.53	53.46
105	1	2200	0.25	49.13	120.21	44.77	101.66	73.22	56.89
106	1	2229	0.21	60.62	130.11	51.18	98.81	74.99	47.63
107	1	2240	0.36	55.33	110.99	49.36	98.40	73.88	49.04
108	1	2251	0.17	60.06	116.67	44.13	104.09	74.11	59.95
109	1	2263	0.40	58.32	121.13	48.79	97.20	73.00	48.40
110	1	2281	0.62	47.04	109.30	47.75	94.93	71.34	47.18
111	1	2295	0.15	88.69	112.98	54.70	98.74	76.72	44.04
112	1	2311	0.65	50.21	114.44	51.34	99.60	75.47	48.25
113	1	2329	0.15	97.56	100.08	54.04	101.22	77.63	47.19
114	1	2341	0.43	42.80	115.18	43.69	102.60	73.15	58.92
115	1	2351	0.41	80.55	109.42	52.65	93.84	73.24	41.19
116	1	2372	0.45	48.82	126.35	56.18	101.06	78.62	44.88
117	1	2401	0.51	49.79	117.66	53.74	100.73	77.23	47.00
118	1	2423	0.51	56.70	119.84	51.92	97.26	74.59	45.33

119	1	2446	0.42	38.27	126.30	49.20	104.53	76.86	55.33
120	1	2458	0.13	96.02	124.84	51.99	101.83	76.91	49.84
121	1	2474	0.27	54.65	119.60	52.18	101.05	76.61	48.87
122	1	2493	0.28	46.98	113.53	51.22	99.35	75.29	48.12
123	1	2524	0.24	53.86	118.12	56.68	99.72	78.20	43.04
124	1	2534	0.31	52.95	111.88	57.19	104.57	80.88	47.38
125	1	2541	0.17	65.43	126.02	60.07	102.15	81.11	42.08
126	1	2552	0.09	49.84	155.31	61.13	103.74	82.44	42.60
127	1	2579	0.61	39.71	113.92	38.99	100.87	69.93	61.88
128	1	2605	0.59	64.68	111.70	47.74	94.01	70.87	46.27
129	1	2626	0.46	60.72	113.97	49.28	98.16	73.72	48.88
130	1	2647	0.42	47.46	127.67	51.17	106.07	78.62	54.90
131	1	2673	0.44	70.81	108.70	51.25	100.99	76.12	49.74
132	1	2680	0.26	48.57	110.19	55.10	104.07	79.58	48.97
133	1	2691	0.18	93.57	105.57	57.24	103.15	80.20	45.91
134	1	2713	0.19	46.94	122.53	52.94	106.11	79.52	53.17
135	1	2722	0.64	66.06	103.35	48.78	95.92	72.35	47.14
136	1	2747	0.31	74.35	125.44	49.19	98.10	73.64	48.90
137	1	2764	0.33	44.56	122.66	43.46	101.49	72.47	58.03
138	1	2784	0.13	98.84	108.87	52.55	101.88	77.21	49.33
139	1	2800	0.37	49.65	106.37	41.63	99.67	70.65	58.04
140	1	2819	0.33	48.47	110.20	50.34	101.60	75.97	51.26
141	1	2863	0.59	78.05	108.70	53.26	99.74	76.50	46.47
142	1	2882	0.14	80.14	152.70	55.04	103.37	79.20	48.33
143	1	2891	0.12	89.32	106.27	58.37	102.64	80.50	44.27
144	1	2913	0.70	53.07	105.29	53.33	93.94	73.63	40.60
145	1	2963	0.41	43.73	102.40	46.01	103.40	74.71	57.40
146	1	2978	0.20	39.21	124.49	51.50	97.15	74.33	45.65
147	1	3012	0.49	41.20	113.01	39.08	101.64	70.36	62.56
148	1	3025	0.11	67.48	130.16	48.17	98.18	73.17	50.01
149	1	3044	0.24	85.15	102.72	54.00	97.47	75.74	43.47
150	1	3063	0.52	63.64	104.84	43.50	96.69	70.09	53.19
151	1	3079	0.07	41.32	136.40	53.03	103.04	78.04	50.01
152	1	3100	0.22	45.77	135.68	53.52	100.39	76.96	46.87
153	1	3110	0.24	94.34	105.42	52.50	102.50	77.50	50.00
154	1	3122	0.26	69.13	137.86	52.69	101.43	77.06	48.74
155	1	3149	0.35	46.72	122.32	46.95	101.74	74.35	54.79
156	1	3173	0.19	82.11	118.56	53.60	95.48	74.54	41.88
157	1	3187	0.53	46.01	109.46	46.99	95.59	71.29	48.60
158	1	3212	0.31	76.78	125.95	49.95	98.01	73.98	48.05
159	1	3240	0.52	50.52	108.70	47.41	96.00	71.71	48.59
160	1	3252	0.06	95.77	107.00	46.88	95.93	71.40	49.05
161	1	3274	0.53	39.61	117.43	43.59	98.40	71.00	54.82
162	1	3300	0.16	81.97	120.41	46.20	102.12	74.16	55.93
163	1	3318	0.66	42.09	108.05	40.87	100.85	70.86	59.97
164	1	3339	0.21	53.99	132.41	53.96	106.67	80.32	52.71
165	1	3345	0.21	58.91	140.14	59.36	106.18	82.77	46.82
166	1	3379	0.36	57.21	132.08	56.65	103.17	79.91	46.52
167	1	3397	0.31	52.79	113.63	39.78	102.19	70.99	62.41
168	1	3409	0.44	54.92	103.78	38.05	99.20	68.62	61.15
169	1	3427	0.17	90.98	104.86	47.03	101.12	74.07	54.09
170	1	3442	0.54	51.42	108.61	46.93	93.04	69.98	46.11
171	1	3465	0.53	43.91	120.40	45.27	97.60	71.44	52.33
172	1	3499	0.33	40.15	128.85	44.07	101.63	72.85	57.55
173	1	3506	0.26	76.08	113.43	43.93	99.47	71.70	55.54
174	1	3523	0.35	55.82	128.78	44.77	100.25	72.51	55.48
175	1	3562	0.63	51.29	107.37	44.58	94.58	69.58	50.00
176	1	3580	0.08	34.66	145.82	42.85	107.46	75.15	64.62
177	1	3607	0.36	50.57	132.80	46.22	96.16	71.19	49.93
178	1	3638	0.64	44.59	114.11	33.34	106.92	70.13	73.58
179	1	3694	0.45	43.31	110.53	42.78	99.17	70.97	56.39
180	1	3711	0.10	94.65	100.37	51.94	98.55	75.25	46.61
181	1	3736	0.45	33.76	125.71	32.53	112.63	72.58	80.10

182	1	3751	0.08	42.39	142.54	44.12	112.53	78.33	68.41
183	1	3797	0.46	40.68	115.00	47.08	103.16	75.12	56.08
184	1	3814	0.21	88.04	104.03	50.49	95.90	73.20	45.41
185	1	3832	0.54	55.36	106.96	36.97	94.11	65.54	57.14
186	1	3857	0.36	33.08	122.82	46.64	95.19	70.91	48.55
187	1	3889	0.47	48.63	109.80	44.51	98.08	71.30	53.57
188	1	3910	0.14	68.57	167.69	52.89	104.43	78.66	51.54
189	1	3947	0.64	41.95	107.57	44.61	98.76	71.68	54.16
190	1	3971	0.31	83.99	105.04	50.48	99.76	75.12	49.29
191	1	3988	0.08	44.25	137.47	44.41	107.66	76.03	63.25
192	1	4007	0.40	40.83	105.58	47.25	102.22	74.74	54.97
193	1	4016	0.42	67.80	99.63	45.89	94.13	70.01	48.23
194	1	4029	0.07	46.76	146.24	49.55	94.83	72.19	45.28
195	1	4039	0.14	95.69	97.52	56.61	95.90	76.26	39.29
196	1	4054	0.28	80.70	118.05	54.89	96.11	75.50	41.21
197	1	4068	0.25	45.21	121.75	43.60	96.29	69.94	52.69
198	1	4084	0.52	37.55	115.33	41.28	98.27	69.77	56.99
199	1	4118	0.37	55.55	126.71	50.66	101.93	76.29	51.27
200	1	4129	0.25	97.74	107.06	43.45	102.24	72.84	58.79
201	1	4143	0.32	39.97	114.30	41.33	103.21	72.27	61.88
202	1	4153	0.24	67.35	122.74	46.57	102.66	74.61	56.10
203	1	4183	0.51	64.67	107.72	43.51	98.19	70.85	54.68
204	1	4191	0.25	47.60	106.91	48.98	103.74	76.36	54.76
205	1	4210	0.07	47.36	138.13	55.08	105.96	80.52	50.88
206	1	4231	0.38	51.04	118.33	53.31	98.90	76.11	45.59
207	1	4238	0.37	63.63	110.36	50.44	99.94	75.19	49.50
208	1	4257	0.41	39.81	130.98	46.93	96.93	71.93	50.00
209	1	4276	0.48	40.18	134.10	44.01	102.07	73.04	58.06
210	1	4295	0.42	44.99	129.34	50.01	100.99	75.50	50.98
211	1	4322	0.60	45.45	107.72	41.25	104.11	72.68	62.86
212	1	4337	0.14	48.83	140.11	54.00	104.39	79.19	50.40
213	1	4361	0.27	53.62	140.19	57.47	107.47	82.47	50.00
214	1	4380	0.42	51.90	127.10	55.75	100.11	77.93	44.37
215	1	4415	0.42	41.52	115.40	49.05	97.80	73.43	48.76
216	1	4442	0.43	64.87	115.92	51.78	95.59	73.68	43.81
217	1	4466	0.24	81.36	105.17	52.90	94.39	73.65	41.49
218	1	4480	0.68	48.63	103.33	47.76	94.81	71.28	47.05
219	1	4518	0.18	44.41	127.58	48.53	100.58	74.55	52.05
220	1	4532	0.49	70.74	100.87	47.10	96.58	71.84	49.48
221	1	4562	0.18	76.50	141.11	52.78	100.61	76.70	47.83
222	1	4579	0.36	44.08	117.28	55.22	103.06	79.14	47.84
223	1	4597	0.08	93.21	137.56	53.30	103.25	78.28	49.95
224	1	4616	0.61	55.26	117.12	56.09	100.01	78.05	43.92
225	1	4638	0.25	52.23	140.39	57.98	101.67	79.83	43.69
226	1	4658	0.41	61.05	111.20	55.11	100.34	77.73	45.23
227	1	4678	0.35	52.52	139.05	59.70	106.74	83.22	47.04
228	1	4709	0.11	69.44	147.75	57.50	103.67	80.59	46.17
229	1	4719	0.48	67.13	115.05	46.95	99.03	72.99	52.08
230	1	4737	0.43	58.51	111.80	40.05	103.82	71.94	63.77
231	1	4773	0.49	53.11	112.21	55.15	99.25	77.20	44.10
232	1	4801	0.43	49.81	104.83	54.82	96.96	75.89	42.14
233	1	4808	0.39	81.65	101.90	54.69	93.49	74.09	38.79
234	1	4836	0.30	40.88	125.17	51.46	105.98	78.72	54.52
235	1	4868	0.65	53.76	109.89	35.01	101.24	68.13	66.23
236	1	4890	0.21	39.17	149.56	48.29	101.07	74.68	52.77
237	1	4904	0.49	76.47	109.39	51.81	95.15	73.48	43.34
238	1	4927	0.43	45.10	125.29	47.29	102.72	75.01	55.43
239	1	4947	0.22	92.77	107.80	53.32	96.02	74.67	42.70
240	1	4966	0.45	74.91	109.64	47.14	99.45	73.30	52.31
241	1	4993	0.17	73.77	132.43	54.88	98.72	76.80	43.84
242	1	5003	0.13	91.54	102.92	57.53	99.59	78.56	42.06
243	1	5015	0.50	50.91	115.48	45.22	103.73	74.48	58.51
244	1	5042	0.48	73.93	117.96	45.87	97.82	71.84	51.94

245	1	5053	0.25	40.66	129.03	51.24	104.20	77.72	52.96
246	1	5061	0.11	43.14	174.93	52.02	109.86	80.94	57.84
247	1	5093	0.71	48.06	113.95	49.58	101.24	75.41	51.66
248	1	5135	0.67	49.05	110.25	50.96	97.20	74.08	46.24
249	1	5172	0.50	45.32	111.31	49.67	101.35	75.51	51.68
250	1	5215	0.12	90.95	105.81	59.43	102.03	80.73	42.60
251	1	5231	0.48	45.96	103.60	51.22	104.67	77.94	53.45
252	1	5271	0.30	54.90	128.46	61.85	104.12	82.98	42.27
253	1	5289	0.08	53.90	146.74	64.20	106.55	85.37	42.35
254	1	5298	0.30	72.90	133.81	59.73	100.43	80.08	40.69
255	1	5313	0.31	55.05	131.99	59.54	100.66	80.10	41.12
256	1	5342	0.42	49.19	123.88	57.46	106.44	81.95	48.99
257	1	5370	0.10	103.14	110.47	57.43	104.64	81.03	47.22
258	1	5387	0.60	44.93	112.84	47.49	103.88	75.68	56.39
259	1	5406	0.14	65.46	161.17	55.44	102.95	79.20	47.52
260	1	5434	0.61	83.11	106.17	49.88	99.24	74.56	49.36
261	1	5471	0.23	44.36	136.07	47.65	108.22	77.94	60.58
262	1	5479	0.17	80.17	123.34	46.38	116.66	81.52	70.28
263	1	5496	0.31	45.52	142.74	53.65	104.28	78.96	50.63
264	1	5516	0.18	48.52	130.52	58.75	109.22	83.99	50.47
265	1	5525	0.26	54.00	134.46	58.51	110.00	84.25	51.49
266	1	5559	0.54	53.75	118.79	60.33	106.08	83.20	45.75
267	1	5566	0.39	75.72	121.05	60.28	107.39	83.83	47.11
268	2	15	0.62	59.08	118.50	60.24	103.42	81.83	43.18
269	2	48	0.07	50.03	113.07	55.48	108.43	81.96	52.96
270	2	67	0.38	44.51	118.64	50.06	101.57	75.81	51.51
271	2	79	0.07	78.68	110.48	50.62	106.27	78.44	55.65
272	2	91	0.10	47.33	127.43	48.83	104.89	76.86	56.05
273	2	109	0.18	47.39	124.79	44.38	104.03	74.21	59.65
274	2	126	0.23	89.45	106.21	46.01	99.36	72.68	53.35
275	2	136	0.20	92.97	103.57	42.52	101.98	72.25	59.46
276	2	147	0.23	56.09	123.10	49.99	106.72	78.36	56.73
277	2	196	0.23	51.74	176.53	60.34	104.52	82.43	44.18
278	2	214	0.43	54.53	125.02	58.47	101.92	80.19	43.44
279	2	236	0.06	98.60	102.89	56.93	103.45	80.19	46.51
280	2	246	0.43	51.17	124.26	52.93	100.43	76.68	47.50
281	2	265	0.12	96.51	124.50	47.81	98.94	73.37	51.12
282	2	278	0.43	39.82	118.74	45.46	103.92	74.69	58.46
283	2	309	0.27	45.29	126.09	49.13	98.40	73.77	49.27
284	2	322	0.12	79.95	110.71	42.69	106.78	74.74	64.09
285	2	329	0.14	46.35	128.77	43.76	103.38	73.57	59.62
286	2	341	0.29	44.11	122.84	51.77	99.11	75.44	47.34
287	2	362	0.25	92.74	118.45	50.86	102.90	76.88	52.04
288	2	382	0.15	96.04	111.04	56.74	101.70	79.22	44.97
289	2	393	0.23	95.31	102.24	50.38	101.13	75.75	50.75
290	2	411	0.63	44.44	113.70	48.07	98.74	73.41	50.67
291	2	443	0.35	76.68	119.65	51.66	101.83	76.75	50.16
292	2	461	0.38	48.01	118.81	51.15	98.80	74.98	47.65
293	2	503	0.28	42.31	130.83	49.07	102.95	76.01	53.88
294	2	517	0.58	59.98	104.62	37.60	101.57	69.59	63.97
295	2	540	0.18	40.89	162.19	53.38	100.59	76.99	47.21
296	2	570	0.58	48.55	108.74	47.11	94.18	70.64	47.07
297	2	598	0.60	44.64	113.27	41.26	99.80	70.53	58.54
298	2	636	0.21	41.69	128.16	42.54	102.57	72.56	60.03
299	2	649	0.23	39.74	128.57	44.35	99.20	71.78	54.85
300	2	680	0.45	49.29	111.71	44.70	97.34	71.02	52.64
301	2	701	0.10	99.76	108.50	45.65	102.74	74.20	57.09
302	2	714	0.25	36.63	137.17	47.67	103.34	75.50	55.67
303	2	749	0.49	40.99	128.05	37.56	109.14	73.35	71.58
304	2	792	0.47	47.22	118.00	50.34	102.22	76.28	51.88
305	2	815	0.07	108.18	109.82	43.69	101.41	72.55	57.72
306	2	830	0.31	44.34	117.35	44.33	102.99	73.66	58.65
307	2	840	0.24	99.48	108.35	48.62	101.99	75.31	53.37

308	2	853	0.30	56.77	133.96	50.40	99.27	74.83	48.87
309	2	888	0.06	107.41	158.71	60.46	106.10	83.28	45.65
310	2	903	0.30	47.03	137.36	56.70	103.24	79.97	46.54
311	2	913	0.30	52.72	122.76	53.24	104.67	78.95	51.43
312	2	930	0.28	51.19	126.96	55.07	105.87	80.47	50.80
313	2	940	0.17	95.72	105.39	52.02	99.91	75.96	47.90
314	2	957	0.56	48.64	114.83	50.08	98.22	74.15	48.14
315	2	982	0.21	92.56	114.00	57.50	103.23	80.36	45.73
316	2	999	0.20	94.37	108.93	55.89	104.41	80.15	48.52
317	2	1010	0.45	50.45	120.29	50.69	105.82	78.26	55.12
318	2	1035	0.63	41.91	117.57	45.88	101.71	73.79	55.83
319	2	1063	0.54	45.68	126.41	50.88	100.06	75.47	49.19
320	2	1102	0.60	44.97	114.74	45.23	102.36	73.79	57.13
321	2	1131	0.20	88.69	100.31	52.74	95.66	74.20	42.92
322	2	1143	0.45	43.33	114.38	38.13	99.88	69.00	61.76
323	2	1168	0.24	101.84	114.52	47.30	98.87	73.08	51.57
324	2	1205	0.23	77.31	146.76	58.98	106.54	82.76	47.57
325	2	1222	0.18	48.55	126.70	57.25	103.64	80.44	46.39
326	2	1232	0.29	46.41	127.16	52.01	99.67	75.84	47.66
327	2	1255	0.34	40.61	113.43	42.50	98.33	70.42	55.82
328	2	1264	0.25	72.21	112.61	47.77	91.02	69.39	43.25
329	2	1279	0.24	46.99	115.56	40.35	101.69	71.02	61.35
330	2	1284	0.29	45.43	119.90	40.73	101.20	70.97	60.47
331	2	1302	0.24	90.82	95.31	51.48	97.88	74.68	46.40
332	2	1311	0.17	36.92	122.89	49.64	102.78	76.21	53.15
333	2	1324	0.15	42.98	113.71	49.94	102.76	76.35	52.83
334	2	1336	0.30	93.63	101.82	52.95	99.80	76.37	46.85
335	2	1346	0.30	67.94	112.14	53.06	99.44	76.25	46.38
336	2	1389	0.74	44.73	108.33	42.34	101.22	71.78	58.88
337	2	1404	0.06	90.79	125.32	56.22	102.24	79.23	46.01
338	2	1429	0.25	38.55	135.08	48.02	102.69	75.36	54.67
339	2	1444	0.29	97.17	116.96	42.77	102.15	72.46	59.38
340	2	1461	0.48	51.37	105.61	42.05	95.47	68.76	53.43
341	2	1471	0.26	91.43	108.44	48.76	101.65	75.20	52.89
342	2	1501	0.29	52.15	141.37	58.01	102.85	80.43	44.84
343	2	1534	0.33	48.12	116.41	44.95	101.53	73.24	56.57
344	2	1560	0.30	78.80	113.82	52.58	97.17	74.87	44.58
345	2	1591	0.61	41.20	113.37	45.99	105.46	75.72	59.46
346	2	1650	0.69	46.33	109.99	40.72	103.99	72.35	63.27
347	2	1659	0.11	35.02	114.76	40.33	107.03	73.68	66.70
348	2	1674	0.19	44.64	124.42	52.68	102.59	77.63	49.91
349	2	1716	0.48	41.94	105.24	43.14	99.81	71.48	56.67
350	2	1749	0.07	105.01	108.16	44.88	100.90	72.89	56.01
351	2	1758	0.27	83.17	121.56	44.76	98.03	71.40	53.27
352	2	1773	0.43	88.03	112.35	43.85	101.97	72.91	58.12
353	2	1810	0.58	40.08	119.24	45.78	103.69	74.74	57.91
354	2	1839	0.15	58.01	200.42	60.71	104.55	82.63	43.83
355	2	1861	0.15	87.20	130.03	54.41	102.30	78.36	47.90
356	2	1873	0.48	49.44	109.33	53.70	103.84	78.77	50.14
357	2	1898	0.09	92.91	119.49	54.90	106.41	80.65	51.52
358	2	1916	0.13	57.55	132.07	55.77	101.38	78.58	45.61
359	2	1930	0.27	95.65	100.33	48.92	100.33	74.62	51.41
360	2	1951	0.23	43.58	108.03	49.28	100.25	74.76	50.97
361	2	1961	0.27	49.02	109.10	43.76	101.88	72.82	58.11
362	2	1985	0.30	65.36	128.41	53.72	100.16	76.94	46.44
363	2	2012	0.42	44.89	128.92	48.31	104.59	76.45	56.27
364	2	2031	0.12	35.45	145.07	41.07	106.53	73.80	65.46
365	2	2040	0.20	37.29	142.91	48.03	101.23	74.63	53.21
366	2	2048	0.20	93.60	110.48	46.70	97.80	72.25	51.10
367	2	2059	0.39	54.48	115.40	42.95	103.17	73.06	60.22
368	2	2077	0.27	49.77	124.44	50.36	102.96	76.66	52.60
369	2	2103	0.29	87.83	113.34	56.08	101.56	78.82	45.48
370	2	2132	0.58	44.98	113.42	41.94	98.08	70.01	56.14

371	2	2156	0.41	45.53	135.40	46.78	97.90	72.34	51.12
372	2	2183	0.55	41.29	121.35	47.08	109.47	78.27	62.40
373	2	2210	0.25	63.80	128.68	50.86	99.03	74.95	48.17
374	2	2221	0.19	48.77	125.10	48.50	98.15	73.33	49.65
375	2	2236	0.35	82.31	111.38	50.44	99.10	74.77	48.66
376	2	2253	0.06	43.71	124.67	45.97	112.35	79.16	66.38
377	2	2267	0.26	52.25	129.73	55.65	101.26	78.46	45.61
378	2	2283	0.34	52.05	139.54	56.52	103.69	80.11	47.17
379	2	2319	0.75	46.90	108.80	42.60	105.50	74.05	62.90
380	2	2335	0.19	93.43	107.09	56.21	98.87	77.54	42.66
381	2	2344	0.19	46.40	142.37	55.49	104.83	80.16	49.34
382	2	2364	0.34	85.03	111.27	53.60	101.38	77.49	47.78
383	2	2376	0.45	47.76	115.88	53.09	103.51	78.30	50.42
384	2	2386	0.11	46.58	136.27	56.50	103.97	80.24	47.48
385	2	2398	0.16	89.56	126.01	60.45	104.14	82.29	43.68
386	2	2407	0.15	49.10	138.11	59.71	105.81	82.76	46.10
387	2	2419	0.20	86.60	117.16	57.26	103.42	80.34	46.16
388	2	2436	0.11	45.89	122.01	47.95	107.85	77.90	59.90
389	2	2450	0.52	46.35	109.06	44.06	104.14	74.10	60.08
390	2	2484	0.21	97.43	111.03	51.82	100.25	76.04	48.43
391	2	2498	0.12	39.96	137.05	47.14	106.53	76.83	59.39
392	2	2513	0.37	87.01	122.81	47.34	99.19	73.27	51.85
393	2	2542	0.19	44.91	123.53	51.15	103.93	77.54	52.78
394	2	2556	0.34	55.26	113.72	46.28	102.38	74.33	56.10
395	2	2567	0.14	89.27	104.17	54.54	98.36	76.45	43.82
396	2	2586	0.09	96.62	99.31	53.56	101.55	77.55	47.99
397	2	2605	0.35	82.16	113.85	51.16	101.19	76.18	50.03
398	2	2623	0.16	42.07	124.73	51.11	106.58	78.84	55.47
399	2	2634	0.27	91.18	104.36	53.38	98.77	76.08	45.39
400	2	2673	0.42	59.28	108.76	50.20	96.28	73.24	46.08
401	2	2693	0.15	43.30	115.31	52.25	104.99	78.62	52.74
402	2	2703	0.28	44.14	131.36	43.63	105.30	74.47	61.66
403	2	2740	0.68	57.07	103.21	41.99	96.84	69.41	54.84
404	2	2772	0.19	94.39	99.74	53.88	98.31	76.09	44.42
405	2	2793	0.50	43.56	107.38	43.13	98.19	70.66	55.07
406	2	2839	0.44	54.49	112.64	57.96	101.31	79.64	43.35
407	2	2899	0.45	45.47	114.30	47.23	107.42	77.32	60.19
408	2	2937	0.46	46.04	119.18	50.46	99.50	74.98	49.03
409	2	2960	0.25	86.61	110.78	55.69	100.10	77.90	44.41
410	2	2994	0.67	47.64	106.37	50.80	100.70	75.75	49.90
411	2	3015	0.06	97.25	105.55	62.53	101.26	81.89	38.72
412	2	3037	0.33	62.90	115.67	52.56	97.43	74.99	44.87
413	2	3051	0.37	61.19	115.22	49.56	101.84	75.70	52.28
414	2	3075	0.49	47.85	127.02	51.23	97.47	74.35	46.23
415	2	3088	0.07	98.29	100.91	54.00	100.78	77.39	46.77
416	2	3103	0.45	44.70	117.49	48.04	101.82	74.93	53.78
417	2	3123	0.15	96.62	105.49	56.12	103.18	79.65	47.07
418	2	3138	0.37	49.61	127.09	54.35	101.52	77.93	47.17
419	2	3150	0.15	97.29	101.24	54.46	102.16	78.31	47.70
420	2	3176	0.59	41.23	111.28	42.32	107.09	74.71	64.77
421	2	3214	0.14	91.33	109.16	53.48	100.91	77.19	47.43
422	2	3223	0.20	46.76	118.73	52.45	103.80	78.12	51.35
423	2	3232	0.44	78.56	106.98	53.53	95.33	74.43	41.80
424	2	3252	0.11	100.46	110.70	48.71	106.46	77.58	57.75
425	2	3268	0.23	93.13	99.82	57.10	97.09	77.09	39.99
426	2	3293	0.65	50.29	108.99	43.49	97.45	70.47	53.96
427	2	3335	0.38	50.07	126.82	51.93	104.70	78.31	52.78
428	2	3348	0.16	97.10	100.07	50.15	100.70	75.43	50.54
429	2	3363	0.43	47.20	112.71	46.69	99.64	73.16	52.94
430	2	3388	0.18	56.80	153.12	55.49	98.51	77.00	43.01
431	2	3424	0.47	45.72	107.29	46.15	100.05	73.10	53.90
432	2	3433	0.28	86.61	115.79	50.59	95.53	73.06	44.94
433	2	3462	0.53	47.74	125.05	52.53	96.97	74.75	44.44

434	2	3490	0.24	53.33	129.42	49.70	101.98	75.84	52.28
435	2	3513	0.55	43.62	111.22	47.88	102.07	74.97	54.19
436	2	3537	0.19	63.29	175.89	57.40	101.04	79.22	43.64
437	2	3558	0.60	41.87	122.25	53.11	102.72	77.91	49.61
438	2	3582	0.09	98.21	100.16	58.04	101.93	79.98	43.89
439	2	3591	0.44	50.20	122.68	54.92	102.72	78.82	47.79
440	2	3605	0.20	47.60	120.06	48.02	107.09	77.55	59.07
441	2	3621	0.15	97.24	102.03	48.66	101.80	75.23	53.15
442	2	3628	0.10	41.54	119.91	46.97	102.44	74.70	55.47
443	2	3637	0.40	44.54	111.89	43.92	101.56	72.74	57.64
444	2	3645	0.37	45.86	114.20	49.50	97.15	73.33	47.65
445	2	3663	0.09	38.27	177.95	49.74	101.81	75.77	52.07
446	2	3685	0.57	49.03	118.99	52.34	98.54	75.44	46.19
447	2	3723	0.61	46.10	107.55	44.98	103.28	74.13	58.30
448	2	3744	0.06	98.50	100.23	55.28	99.75	77.52	44.47
449	2	3762	0.63	41.74	112.10	47.58	99.14	73.36	51.55
450	2	3793	0.14	88.11	105.59	53.03	94.25	73.64	41.22
451	2	3803	0.53	74.33	107.00	49.68	95.60	72.64	45.92
452	2	3817	0.09	99.29	105.12	47.94	99.50	73.72	51.56
453	2	3829	0.16	99.29	109.35	45.27	103.98	74.62	58.71
454	2	3843	0.36	47.87	113.70	47.35	102.62	74.99	55.27
455	2	3872	0.23	59.52	143.05	60.56	101.61	81.09	41.05
456	2	3895	0.18	58.91	148.66	58.50	103.65	81.07	45.15
457	2	3915	0.53	48.61	117.50	47.28	105.00	76.14	57.72
458	2	3939	0.51	41.94	119.75	45.01	102.68	73.85	57.67
459	2	3950	0.07	97.54	109.58	52.84	110.52	81.68	57.68
460	2	3963	0.43	55.44	128.41	54.55	101.91	78.23	47.36
461	2	3998	0.53	50.65	112.70	53.60	101.48	77.54	47.88
462	2	4013	0.10	97.56	100.42	54.36	100.02	77.19	45.66
463	2	4023	0.43	45.44	115.09	46.30	99.74	73.02	53.44
464	2	4042	0.30	59.88	108.90	52.72	101.18	76.95	48.46
465	2	4060	0.17	52.87	119.93	58.18	100.95	79.56	42.77
466	2	4066	0.15	98.98	104.79	58.17	102.59	80.38	44.42
467	2	4089	0.36	52.58	120.54	59.35	100.97	80.16	41.62
468	2	4106	0.20	71.35	120.60	58.11	105.35	81.73	47.24
469	2	4123	0.11	51.77	139.51	53.84	107.38	80.61	53.53
470	2	4146	0.69	45.02	108.32	45.68	103.44	74.56	57.76
471	2	4185	0.17	51.62	171.51	59.65	104.36	82.01	44.71
472	2	4210	0.57	56.28	117.96	48.68	100.10	74.39	51.41
473	2	4223	0.16	88.50	110.49	55.68	99.37	77.53	43.70
474	2	4239	0.27	45.24	130.98	48.86	110.74	79.80	61.89
475	2	4262	0.34	52.92	124.61	58.56	104.07	81.31	45.51
476	2	4281	0.12	96.50	104.16	62.93	105.74	84.34	42.80
477	2	4298	0.52	52.48	113.95	58.72	102.90	80.81	44.18
478	2	4311	0.19	50.13	160.37	57.96	105.68	81.82	47.73
479	2	4337	0.44	52.44	129.55	58.70	97.50	78.10	38.80
480	2	4352	0.19	95.68	119.71	54.35	101.70	78.02	47.34
481	2	4372	0.55	43.80	117.45	48.46	102.31	75.39	53.85
482	2	4414	0.41	50.75	121.05	54.00	105.27	79.64	51.27
483	2	4422	0.08	49.33	118.33	54.87	106.47	80.67	51.60
484	2	4433	0.15	50.83	112.19	55.54	99.76	77.65	44.21
485	2	4460	0.41	46.26	105.26	50.91	101.72	76.31	50.82
486	2	4494	0.27	61.96	139.45	59.02	114.22	86.62	55.20
487	3	32	0.43	54.53	109.25	25.82	103.73	64.78	77.91
488	3	39	0.28	33.45	102.55	38.68	99.06	68.87	60.38
489	3	60	0.09	32.07	118.75	45.52	98.07	71.79	52.56
490	3	89	0.07	86.70	118.42	46.69	98.41	72.55	51.72
491	3	124	0.43	46.68	108.98	41.78	92.66	67.22	50.88
492	3	157	0.08	82.91	103.85	52.10	99.07	75.59	46.97
493	3	204	0.35	55.00	113.44	44.02	100.39	72.21	56.37
494	3	217	0.11	43.72	98.89	40.25	103.78	72.01	63.53
495	3	229	0.20	45.19	113.50	43.65	100.83	72.24	57.17
496	3	236	0.12	43.25	119.14	45.75	98.45	72.10	52.70

497	3	240	0.12	37.41	121.29	45.40	100.40	72.90	55.00
498	3	243	0.12	38.92	116.99	50.25	97.84	74.04	47.59
499	3	282	0.40	77.03	115.24	47.15	92.73	69.94	45.57
500	3	318	0.33	55.81	103.77	39.50	99.19	69.34	59.69
501	3	327	0.33	44.12	108.76	47.80	96.78	72.29	48.98
502	3	371	0.44	47.45	122.44	39.46	94.76	67.11	55.30
503	3	392	0.13	41.86	143.56	40.04	104.53	72.29	64.49
504	3	403	0.39	75.24	107.59	41.03	97.98	69.51	56.95
505	3	410	0.33	43.25	122.91	41.37	99.37	70.37	58.00
506	3	434	0.08	94.34	100.27	55.73	102.13	78.93	46.41
507	3	462	0.47	43.87	115.95	49.44	99.66	74.55	50.22
508	3	500	0.20	43.18	127.94	49.34	97.43	73.38	48.08
509	3	524	0.45	41.76	117.94	41.34	97.38	69.36	56.04
510	3	560	0.07	97.27	102.43	48.88	98.27	73.57	49.39
511	3	569	0.53	41.49	126.66	41.45	103.01	72.23	61.56
512	3	602	0.49	55.54	106.83	42.37	97.28	69.82	54.91
513	3	610	0.18	44.50	113.73	43.66	97.30	70.48	53.63
514	3	634	0.26	46.86	120.25	43.19	98.42	70.80	55.23
515	3	650	0.20	94.40	102.05	43.45	99.11	71.28	55.66
516	3	664	0.47	55.78	116.11	44.50	96.71	70.60	52.20
517	3	699	0.22	44.56	135.57	51.24	99.08	75.16	47.84
518	3	707	0.14	45.09	118.02	43.39	101.79	72.59	58.39
519	3	714	0.19	43.02	102.10	42.75	105.93	74.34	63.18
520	3	725	0.52	75.15	102.83	45.44	97.01	71.22	51.57
521	3	768	0.63	46.16	108.93	48.59	99.58	74.09	50.98
522	3	804	0.23	50.10	168.93	50.20	100.44	75.32	50.24
523	3	823	0.55	47.69	113.08	42.80	93.49	68.15	50.69
524	3	843	0.10	38.11	114.13	46.63	98.32	72.48	51.68
525	3	855	0.40	45.29	114.82	41.32	99.48	70.40	58.16
526	3	872	0.14	43.99	165.52	52.25	104.89	78.57	52.64
527	3	903	0.55	55.51	121.14	44.23	100.31	72.27	56.08
528	3	941	0.52	40.61	118.46	48.02	96.65	72.34	48.63
529	3	957	0.08	98.46	103.53	51.44	101.72	76.58	50.28
530	3	975	0.15	46.01	126.15	44.76	100.75	72.75	55.99
531	3	988	0.43	41.03	107.12	35.56	108.91	72.24	73.35
532	3	1001	0.14	91.98	129.18	52.39	99.58	75.98	47.19
533	3	1050	0.47	80.82	103.23	49.24	94.60	71.92	45.36
534	3	1058	0.61	49.43	116.70	46.47	95.04	70.76	48.56
535	3	1146	0.83	40.00	109.01	43.08	100.85	71.96	57.77
536	3	1195	0.76	40.11	105.51	41.98	100.52	71.25	58.54
537	3	1234	0.11	92.19	108.69	47.49	98.60	73.04	51.11
538	3	1247	0.08	40.39	117.65	45.12	105.09	75.10	59.96
539	3	1258	0.64	53.20	104.24	41.97	96.68	69.32	54.71
540	3	1279	0.23	49.82	131.71	49.65	99.44	74.54	49.79
541	3	1303	0.12	95.80	108.51	51.89	97.40	74.64	45.51
542	3	1325	0.59	40.53	107.78	31.62	99.11	65.37	67.49
543	3	1343	0.26	72.29	125.40	38.05	101.27	69.66	63.22
544	3	1370	0.50	44.34	112.09	39.92	100.59	70.25	60.67
545	3	1384	0.21	83.64	112.62	46.54	95.93	71.23	49.39
546	3	1410	0.50	40.81	122.70	31.74	93.06	62.40	61.31
547	3	1436	0.11	89.61	93.08	49.81	97.02	73.42	47.21
548	3	1453	0.53	47.47	107.36	40.47	98.52	69.50	58.05
549	3	1479	0.08	95.22	97.82	52.49	97.35	74.92	44.85
550	3	1513	0.30	39.35	121.34	44.89	93.98	69.43	49.08
551	3	1535	0.37	42.37	103.63	44.14	95.87	70.00	51.73
552	3	1549	0.18	93.76	100.60	48.57	96.25	72.41	47.68
553	3	1578	0.16	47.46	131.66	43.87	99.58	71.73	55.71
554	3	1596	0.28	48.59	109.60	34.17	101.85	68.01	67.67
555	3	1612	0.35	49.69	119.73	33.63	102.79	68.21	69.16
556	3	1644	0.07	91.64	125.93	57.88	97.98	77.93	40.09
557	3	1676	0.77	42.96	102.38	39.35	98.20	68.78	58.85
558	3	1700	0.09	98.53	100.09	48.01	101.54	74.78	53.53
559	3	1722	0.41	36.11	125.69	34.40	104.04	69.22	69.65

560	3	1744	0.24	87.00	100.82	43.96	95.39	69.67	51.43
561	3	1752	0.37	38.47	118.99	39.81	100.87	70.34	61.06
562	3	1765	0.09	44.34	147.14	50.54	100.62	75.58	50.08
563	3	1788	0.14	91.97	100.04	56.98	95.90	76.44	38.92
564	3	1819	0.70	58.34	101.92	44.35	93.50	68.92	49.15
565	3	1866	0.48	42.99	109.33	44.23	99.94	72.08	55.71
566	3	1917	0.24	52.70	117.32	50.19	102.53	76.36	52.34
567	3	1932	0.29	57.40	107.66	45.03	102.33	73.68	57.29
568	3	1946	0.15	40.08	112.02	51.81	96.00	73.91	44.20
569	3	1970	0.56	40.23	118.88	41.82	96.87	69.34	55.05
570	3	2025	0.15	39.88	137.78	41.76	97.58	69.67	55.82
571	3	2036	0.48	56.36	104.32	42.47	95.39	68.93	52.92
572	3	2051	0.18	89.47	105.36	47.53	98.20	72.86	50.67
573	3	2071	0.14	46.79	142.96	43.88	104.47	74.18	60.59
574	3	2105	0.29	47.19	110.42	45.11	104.52	74.81	59.41
575	3	2116	0.15	76.76	115.17	47.01	99.91	73.46	52.90
576	3	2129	0.15	81.42	114.31	50.58	97.01	73.79	46.43
577	3	2143	0.40	38.35	137.43	51.00	97.93	74.46	46.93
578	3	2164	0.07	91.68	110.11	56.15	96.02	76.08	39.87
579	3	2198	0.64	45.12	100.12	36.63	92.44	64.54	55.82
580	3	2217	0.15	93.06	95.02	45.14	95.57	70.36	50.44
581	3	2239	0.24	83.27	100.35	51.76	92.37	72.07	40.61
582	3	2277	0.46	44.92	107.50	38.71	95.50	67.11	56.79
583	3	2302	0.25	78.48	98.92	44.72	93.65	69.19	48.93
584	3	2336	0.29	39.98	116.20	40.21	99.07	69.64	58.87
585	3	2367	0.21	80.36	91.57	43.63	92.93	68.28	49.30
586	3	2376	0.26	52.13	102.82	42.99	91.55	67.27	48.56
587	3	2384	0.36	35.95	108.76	38.39	95.75	67.07	57.36
588	3	2422	0.42	42.77	104.54	38.19	97.68	67.94	59.49
589	3	2433	0.39	42.23	103.32	27.44	105.07	66.25	77.62

Eshaghi_Rep1

Index	Chr	Location (kb)	Fi	Ts	Te	T0	T100	T50	DT
1	1	18	0.06	37.40	59.97	37.98	62.08	50.03	24.10
2	1	35	0.22	47.75	56.52	42.11	61.43	51.77	19.32
3	1	65	0.15	23.31	69.63	23.19	68.29	45.74	45.10
4	1	89	0.25	9.16	66.45	23.08	60.58	41.83	37.49
5	1	106	0.13	3.27	82.41	-1.27	83.96	41.34	85.23
6	1	127	0.21	0.49	96.58	-0.17	71.42	35.63	71.60
7	1	148	0.34	8.46	74.42	7.61	58.34	32.98	50.72
8	1	166	0.16	13.65	90.33	8.44	67.21	37.82	58.77
9	1	186	0.23	8.31	83.38	9.57	63.77	36.67	54.20
10	1	205	0.39	8.87	76.14	15.00	53.64	34.32	38.64
11	1	242	0.38	21.89	58.72	22.45	55.00	38.73	32.55
12	1	257	0.18	22.68	50.86	25.98	58.68	42.33	32.70
13	1	277	0.32	24.57	55.34	27.74	57.17	42.45	29.43
14	1	300	0.05	27.20	96.17	35.51	60.46	47.98	24.95
15	1	325	0.58	19.73	53.31	16.84	47.17	32.01	30.33
16	1	340	0.05	45.11	51.12	38.00	51.02	44.51	13.02
17	1	369	0.59	13.72	58.21	18.28	60.60	39.44	42.32
18	1	386	0.09	38.27	67.43	23.13	60.54	41.83	37.41
19	1	411	0.31	35.28	59.17	33.43	56.66	45.05	23.23
20	1	427	0.07	25.61	60.04	28.09	58.09	43.09	30.00
21	1	438	0.48	19.95	57.22	24.65	58.17	41.41	33.52
22	1	475	0.21	15.03	79.95	13.71	62.53	38.12	48.82
23	1	489	0.45	14.72	58.55	14.90	45.97	30.43	31.07
24	1	517	0.32	30.55	63.54	26.13	57.14	41.64	31.02
25	1	534	0.06	23.41	89.50	25.00	63.08	44.04	38.08
26	1	550	0.19	42.46	53.60	24.19	54.78	39.48	30.59
27	1	563	0.49	11.50	59.92	14.27	55.11	34.69	40.84

28	1	611	0.45	34.49	53.51	24.45	57.37	40.91	32.93
29	1	637	0.10	45.02	47.43	37.65	50.46	44.05	12.81
30	1	651	0.57	17.62	59.78	14.50	54.73	34.61	40.23
31	1	669	0.16	26.57	76.68	28.11	59.07	43.59	30.96
32	1	698	0.06	48.11	57.71	27.15	60.14	43.64	32.99
33	1	713	0.49	9.32	61.08	6.54	61.19	33.87	54.65
34	1	755	0.42	22.92	58.84	25.73	52.11	38.92	26.38
35	1	796	0.51	22.62	57.30	18.46	61.44	39.95	42.97
36	1	820	0.27	31.71	59.79	28.37	52.03	40.20	23.66
37	1	843	0.58	13.11	60.87	13.82	58.25	36.03	44.43
38	1	878	0.64	12.00	53.83	16.75	42.50	29.63	25.74
39	1	896	0.15	10.84	98.23	16.93	57.95	37.44	41.02
40	1	916	0.49	5.25	71.35	6.85	64.37	35.61	57.53
41	1	947	0.18	33.15	62.28	22.52	57.96	40.24	35.43
42	1	968	0.38	12.72	59.09	13.59	57.39	35.49	43.80
43	1	982	0.10	16.84	77.00	22.61	57.76	40.19	35.16
44	1	1003	0.05	41.74	59.33	37.81	51.33	44.57	13.52
45	1	1030	0.47	20.54	52.37	15.82	56.35	36.09	40.54
46	1	1061	0.15	41.12	52.18	23.49	54.49	38.99	31.00
47	1	1076	0.28	6.04	76.66	19.44	55.55	37.49	36.10
48	1	1109	0.36	2.83	64.36	12.77	60.97	36.87	48.21
49	1	1119	0.22	25.10	57.74	22.82	55.00	38.91	32.18
50	1	1137	0.21	6.06	93.85	-5.99	61.80	27.90	67.79
51	1	1150	0.52	-11.30	70.87	-6.10	64.49	29.20	70.59
52	1	1168	0.14	38.00	53.87	24.25	54.11	39.18	29.87
53	1	1184	0.21	35.76	61.39	15.00	50.00	32.50	35.00
54	1	1195	0.33	2.78	58.17	10.96	58.36	34.66	47.40
55	1	1227	0.17	21.40	70.30	20.96	50.00	35.48	29.04
56	1	1247	0.20	8.73	57.56	14.97	52.49	33.73	37.52
57	1	1257	0.41	6.26	60.76	12.34	53.18	32.76	40.84
58	1	1277	0.34	16.47	62.96	14.23	53.24	33.74	39.02
59	1	1302	0.14	40.63	52.98	36.66	52.26	44.46	15.60
60	1	1319	0.30	24.84	58.08	27.25	60.99	44.12	33.74
61	1	1330	0.30	20.30	54.47	29.52	53.13	41.32	23.61
62	1	1352	0.27	26.21	58.37	26.50	57.42	41.96	30.93
63	1	1385	0.57	14.23	52.15	16.81	50.00	33.40	33.19
64	1	1425	0.40	11.84	61.70	14.36	56.52	35.44	42.17
65	1	1444	0.24	9.63	77.26	17.88	42.01	29.95	24.13
66	1	1457	0.22	-7.28	98.75	-6.34	72.06	32.86	78.40
67	1	1482	0.30	6.35	73.17	23.04	49.10	36.07	26.06
68	1	1506	0.23	3.34	79.95	16.88	58.52	37.70	41.64
69	1	1525	0.09	40.25	58.52	27.42	55.51	41.47	28.09
70	1	1535	0.24	21.88	60.42	12.14	63.36	37.75	51.22
71	1	1545	0.11	21.20	67.54	12.46	62.12	37.29	49.66
72	1	1576	0.36	19.36	56.44	14.37	61.42	37.89	47.05
73	1	1605	0.16	39.96	50.83	37.26	50.50	43.88	13.24
74	1	1620	0.31	24.76	59.95	21.52	61.00	41.26	39.48
75	1	1651	0.22	25.16	62.08	28.27	57.94	43.11	29.67
76	1	1658	0.13	34.44	59.60	28.07	57.54	42.80	29.47
77	1	1670	0.38	18.56	63.76	22.75	59.26	41.01	36.51
78	1	1698	0.18	7.13	85.99	16.54	51.13	33.83	34.59
79	1	1717	0.43	6.19	59.52	27.51	34.46	30.99	6.96
80	1	1746	0.47	5.46	63.82	10.25	60.76	35.50	50.51
81	1	1766	0.10	13.77	81.27	13.74	59.74	36.74	46.00
82	1	1776	0.42	24.66	53.89	24.56	47.12	35.84	22.56
83	1	1802	0.41	8.32	71.70	11.37	64.36	37.86	52.99
84	1	1831	0.39	4.35	69.79	15.00	60.73	37.87	45.73
85	1	1848	0.19	36.51	52.48	23.41	52.42	37.91	29.02
86	1	1866	0.51	8.09	59.27	12.46	55.00	33.73	42.54
87	1	1895	0.19	23.80	56.95	15.96	61.58	38.77	45.62
88	1	1903	0.21	28.52	51.12	29.17	51.51	40.34	22.35
89	1	1915	0.33	22.28	51.43	33.22	50.56	41.89	17.34
90	1	1931	0.32	16.88	59.82	21.08	58.11	39.60	37.03

91	1	1950	0.14	25.23	71.38	27.62	60.51	44.07	32.89
92	1	1975	0.44	8.98	57.55	15.53	36.49	26.01	20.96
93	1	1994	0.41	11.63	59.85	18.77	53.61	36.19	34.84
94	1	2019	0.41	31.15	55.38	37.23	50.44	43.83	13.21
95	1	2043	0.51	17.50	60.48	16.39	54.59	35.49	38.20
96	1	2078	0.41	20.32	56.99	22.85	58.85	40.85	36.00
97	1	2111	0.17	32.96	58.22	32.79	52.73	42.76	19.94
98	1	2136	0.23	32.50	53.98	35.43	51.37	43.40	15.94
99	1	2146	0.24	27.71	54.44	33.93	56.25	45.09	22.32
100	1	2158	0.25	28.78	62.19	28.66	59.81	44.23	31.15
101	1	2176	0.05	18.63	101.08	24.19	60.79	42.49	36.60
102	1	2193	0.35	9.56	65.48	11.16	62.33	36.75	51.16
103	1	2210	0.08	47.54	58.19	22.45	56.84	39.64	34.39
104	1	2220	0.14	15.65	71.63	12.41	62.68	37.54	50.27
105	1	2234	0.24	25.58	63.55	25.00	45.36	35.18	20.36
106	1	2243	0.22	7.13	73.90	14.10	56.47	35.28	42.37
107	1	2265	0.20	-3.45	97.90	11.81	58.02	34.92	46.21
108	1	2279	0.39	20.62	56.36	13.64	46.09	29.86	32.45
109	1	2308	0.54	27.35	57.21	18.10	59.22	38.66	41.13
110	1	2341	0.58	16.46	54.76	15.00	57.98	36.49	42.98
111	1	2365	0.38	25.58	57.30	26.88	51.87	39.37	24.99
112	1	2396	0.48	22.50	57.51	19.11	57.07	38.09	37.96
113	1	2419	0.12	32.73	72.39	16.34	59.06	37.70	42.73
114	1	2437	0.54	4.56	62.36	11.87	52.65	32.26	40.78
115	1	2486	0.40	12.03	56.22	17.89	61.15	39.52	43.26
116	1	2497	0.19	18.49	71.96	14.03	61.42	37.72	47.38
117	1	2530	0.59	19.26	56.26	23.21	50.53	36.87	27.31
118	1	2558	0.10	24.94	98.34	28.59	63.20	45.89	34.61
119	1	2565	0.11	44.95	54.46	26.01	56.10	41.05	30.09
120	1	2576	0.44	21.16	59.73	28.00	34.37	31.18	6.36
121	1	2602	0.48	7.82	67.71	7.27	68.12	37.69	60.85
122	1	2639	0.38	25.43	62.20	18.11	59.39	38.75	41.28
123	1	2678	0.57	35.16	50.06	33.56	50.98	42.27	17.42
124	1	2692	0.23	40.88	51.79	39.01	50.61	44.81	11.60
125	1	2714	0.18	28.09	56.04	14.80	55.00	34.90	40.20
126	1	2722	0.41	17.40	58.31	13.78	57.44	35.61	43.66
127	1	2751	0.44	16.74	61.41	14.12	56.21	35.16	42.09
128	1	2772	0.14	49.78	56.55	14.24	58.61	36.42	44.38
129	1	2788	0.51	3.95	66.22	9.71	56.19	32.95	46.48
130	1	2820	0.29	10.42	71.84	13.98	61.90	37.94	47.93
131	1	2838	0.17	25.69	64.65	26.65	58.59	42.62	31.94
132	1	2850	0.12	32.97	57.10	28.33	50.70	39.52	22.37
133	1	2861	0.40	18.85	58.99	13.44	59.64	36.54	46.20
134	1	2886	0.26	30.78	54.78	22.17	55.00	38.59	32.83
135	1	2912	0.44	18.91	56.90	13.32	55.00	34.16	41.68
136	1	2936	0.08	10.56	71.27	22.66	59.53	41.10	36.87
137	1	2965	0.32	9.18	61.59	16.53	57.55	37.04	41.02
138	1	2979	0.09	33.14	69.77	16.15	58.64	37.39	42.49
139	1	2991	0.06	45.09	61.07	9.95	65.22	37.58	55.28
140	1	3045	0.41	-2.15	80.61	-8.98	70.97	31.00	79.95
141	1	3080	0.34	11.84	68.29	8.26	61.78	35.02	53.52
142	1	3118	0.20	40.05	66.33	24.32	56.23	40.28	31.91
143	1	3134	0.28	15.17	52.28	24.26	52.32	38.29	28.06
144	1	3146	0.35	20.72	56.42	16.49	53.78	35.13	37.29
145	1	3170	0.12	44.31	48.25	28.20	52.69	40.44	24.49
146	1	3184	0.39	15.78	64.23	15.87	55.41	35.64	39.54
147	1	3210	0.19	16.74	61.71	19.87	60.50	40.19	40.64
148	1	3228	0.20	19.24	61.58	21.03	61.91	41.47	40.88
149	1	3263	0.36	13.05	66.44	17.12	55.00	36.06	37.88
150	1	3284	0.16	10.69	80.29	25.54	51.16	38.35	25.62
151	1	3298	0.22	8.51	76.23	19.24	63.51	41.38	44.27
152	1	3311	0.10	15.05	76.63	19.55	61.86	40.71	42.31
153	1	3330	0.10	16.02	65.46	27.83	53.59	40.71	25.76

154	1	3348	0.44	32.09	50.43	29.03	50.92	39.97	21.88
155	1	3356	0.09	38.62	48.54	32.61	50.90	41.75	18.29
156	1	3371	0.20	28.77	61.16	30.91	50.59	40.75	19.68
157	1	3390	0.57	7.26	59.43	8.61	55.00	31.81	46.39
158	1	3416	0.07	39.45	61.65	9.65	62.54	36.10	52.88
159	1	3430	0.34	3.86	66.06	-3.30	66.66	31.68	69.97
160	1	3458	0.28	23.58	54.18	23.63	52.87	38.25	29.24
161	1	3481	0.44	6.95	64.67	1.48	61.67	31.57	60.19
162	1	3510	0.33	17.12	62.87	12.20	55.93	34.07	43.73
163	1	3528	0.38	13.96	65.18	20.86	52.73	36.80	31.87
164	1	3558	0.32	30.20	51.85	27.99	45.36	36.68	17.37
165	1	3581	0.25	11.00	77.40	14.90	65.77	40.33	50.87
166	1	3597	0.06	11.65	76.16	18.63	61.30	39.97	42.66
167	1	3608	0.09	33.12	59.76	17.66	55.24	36.45	37.58
168	1	3619	0.52	10.07	56.19	-2.23	61.69	29.73	63.92
169	1	3657	0.58	13.31	51.83	17.46	51.74	34.60	34.28
170	1	3676	0.08	31.25	62.92	19.22	55.50	37.36	36.28
171	1	3685	0.53	9.55	56.43	-2.68	61.24	29.28	63.92
172	1	3697	0.19	30.13	47.58	23.06	45.52	34.29	22.46
173	1	3715	0.61	-7.57	53.30	-2.26	58.26	28.00	60.52
174	1	3726	0.11	13.01	92.43	9.99	58.69	34.34	48.70
175	1	3752	0.37	22.61	70.44	28.76	68.07	48.41	39.32
176	1	3802	0.46	2.02	75.04	-5.93	57.58	25.82	63.51
177	1	3827	0.43	9.72	62.93	20.95	64.98	42.96	44.03
178	1	3839	0.06	13.33	77.28	19.72	65.84	42.78	46.12
179	1	3847	0.13	46.99	52.65	31.68	56.35	44.01	24.67
180	1	3865	0.11	18.55	88.31	28.84	61.36	45.10	32.52
181	1	3895	0.27	18.14	61.43	19.65	53.67	36.66	34.02
182	1	3911	0.23	15.54	51.67	21.56	56.41	38.98	34.85
183	1	3938	0.39	6.34	62.67	-1.25	62.83	30.79	64.08
184	1	3949	0.18	20.91	71.39	10.30	60.76	35.53	50.46
185	1	3970	0.63	-0.99	67.17	-11.29	70.68	29.70	81.97
186	1	3989	0.10	47.09	53.33	10.09	67.08	38.59	56.99
187	1	4010	0.46	2.06	78.57	-3.74	65.15	30.70	68.89
188	1	4039	0.27	27.54	65.74	-3.88	64.16	30.14	68.04
189	1	4050	0.36	-10.14	64.59	0.05	62.11	31.08	62.06
190	1	4079	0.24	35.34	58.01	28.00	56.84	42.42	28.84
191	1	4106	0.45	8.73	58.60	22.27	48.14	35.20	25.87
192	1	4124	0.14	19.84	64.16	16.64	62.53	39.59	45.89
193	1	4128	0.17	11.68	52.04	17.98	60.82	39.40	42.85
194	1	4143	0.29	13.01	54.65	21.43	54.50	37.97	33.07
195	1	4169	0.11	12.15	83.82	18.02	59.46	38.74	41.44
196	1	4186	0.19	15.74	59.61	21.30	56.17	38.74	34.87
197	1	4198	0.35	13.68	57.95	16.95	58.31	37.63	41.36
198	1	4218	0.05	14.30	69.95	22.17	56.17	39.17	33.99
199	1	4231	0.31	20.54	66.77	19.46	58.72	39.09	39.26
200	1	4260	0.23	20.11	75.36	19.22	52.64	35.93	33.41
201	1	4288	0.13	8.16	80.61	7.75	63.68	35.71	55.93
202	1	4300	0.15	11.04	77.95	17.56	56.38	36.97	38.82
203	1	4305	0.17	41.72	56.59	21.22	56.01	38.62	34.79
204	1	4322	0.44	19.30	55.10	19.67	48.29	33.98	28.62
205	1	4368	0.43	28.27	52.47	28.03	51.21	39.62	23.18
206	1	4415	0.54	15.17	57.25	12.83	56.61	34.72	43.78
207	1	4436	0.19	41.20	48.06	25.46	50.00	37.73	24.54
208	1	4446	0.47	18.87	59.63	17.89	61.45	39.67	43.56
209	1	4478	0.37	5.70	68.72	23.15	55.91	39.53	32.76
210	1	4488	0.08	13.44	76.98	15.94	57.49	36.71	41.55
211	1	4499	0.12	45.69	53.92	23.92	52.80	38.36	28.88
212	1	4511	0.22	10.68	63.66	15.01	58.03	36.52	43.02
213	1	4518	0.13	10.30	64.96	11.30	62.00	36.65	50.70
214	1	4533	0.28	23.46	63.49	27.85	34.05	30.95	6.20
215	1	4545	0.16	2.93	87.76	5.06	69.03	37.05	63.98
216	1	4565	0.32	24.15	61.20	29.45	46.08	37.76	16.64

217	1	4579	0.26	18.64	73.59	22.25	62.52	42.38	40.27
218	1	4601	0.23	23.64	57.59	28.93	52.18	40.56	23.24
219	1	4617	0.33	30.16	56.46	31.72	52.48	42.10	20.77
220	1	4641	0.20	25.92	60.20	27.89	58.75	43.32	30.86
221	1	4657	0.08	24.10	52.33	36.92	52.00	44.46	15.08
222	1	4675	0.38	26.55	55.24	32.21	53.30	42.75	21.09
223	1	4702	0.21	31.54	62.75	31.86	53.17	42.52	21.32
224	1	4724	0.11	43.75	52.79	39.53	50.95	45.24	11.42
225	1	4738	0.53	20.94	51.67	16.92	57.34	37.13	40.41
226	1	4780	0.35	26.94	56.54	19.65	53.65	36.65	34.00
227	1	4796	0.21	19.53	53.47	27.60	57.49	42.55	29.89
228	1	4805	0.23	25.32	56.76	27.82	52.06	39.94	24.24
229	1	4811	0.21	39.62	53.10	27.93	51.88	39.91	23.96
230	1	4848	0.45	15.07	56.14	16.21	51.36	33.78	35.16
231	1	4862	0.05	5.96	62.47	13.52	63.53	38.52	50.01
232	1	4874	0.37	7.24	63.91	11.17	60.38	35.77	49.21
233	1	4895	0.07	52.76	58.27	20.56	60.00	40.28	39.43
234	1	4906	0.14	16.17	78.74	20.56	56.71	38.63	36.15
235	1	4918	0.21	1.05	84.47	15.00	66.40	40.70	51.40
236	1	4945	0.31	1.69	70.08	8.96	63.28	36.12	54.32
237	1	4959	0.14	21.90	79.50	16.31	56.63	36.47	40.33
238	1	4981	0.26	11.71	70.03	13.33	60.66	37.00	47.33
239	1	5002	0.38	11.74	61.88	10.01	58.07	34.04	48.06
240	1	5024	0.29	10.35	79.49	10.78	50.35	30.56	39.58
241	1	5044	0.12	5.69	98.10	13.60	65.48	39.54	51.88
242	1	5055	0.31	8.17	66.16	17.05	61.34	39.20	44.29
243	1	5064	0.18	19.33	63.99	8.88	62.95	35.92	54.08
244	1	5101	0.62	16.90	48.99	6.95	55.48	31.22	48.53
245	1	5121	0.23	32.95	51.49	26.62	50.00	38.31	23.38
246	1	5138	0.43	2.58	78.70	18.60	62.58	40.59	43.98
247	1	5155	0.06	44.78	57.87	26.32	60.51	43.42	34.19
248	1	5176	0.53	9.07	50.68	10.53	50.00	30.26	39.47
249	1	5190	0.19	6.28	62.84	-4.03	63.51	29.74	67.54
250	1	5205	0.15	25.37	68.29	21.45	56.35	38.90	34.90
251	1	5227	0.32	6.21	70.40	21.45	63.26	42.36	41.81
252	1	5259	0.43	17.66	53.27	19.35	50.00	34.67	30.66
253	1	5296	0.26	27.15	52.43	27.55	50.39	38.97	22.85
254	1	5321	0.30	25.14	55.18	26.22	52.89	39.55	26.66
255	1	5338	0.13	18.55	58.77	27.17	60.16	43.67	32.98
256	1	5353	0.14	20.28	63.95	23.35	60.27	41.81	36.92
257	1	5366	0.18	20.99	66.26	27.83	60.22	44.03	32.40
258	1	5379	0.16	20.96	63.69	24.23	50.28	37.25	26.05
259	1	5393	0.46	10.52	59.18	15.62	35.22	25.42	19.60
260	1	5410	0.27	-1.66	93.48	-0.70	65.92	32.61	66.63
261	1	5438	0.16	4.83	85.28	-2.78	67.23	32.23	70.02
262	1	5461	0.18	3.28	60.22	12.81	68.87	40.84	56.06
263	1	5472	0.13	6.21	65.45	11.72	63.22	37.47	51.49
264	1	5479	0.10	16.76	63.56	14.01	55.80	34.90	41.80
265	1	5509	0.11	25.36	59.34	33.42	60.75	47.09	27.34
266	1	5519	0.20	32.06	59.32	36.00	61.33	48.66	25.33
267	1	5536	0.11	31.80	61.12	37.75	61.32	49.53	23.57
268	1	5577	0.23	30.60	62.07	28.15	65.45	46.80	37.30
269	2	7	0.15	22.05	63.87	23.40	67.92	45.66	44.52
270	2	25	0.55	25.54	58.92	27.81	62.31	45.06	34.50
271	2	38	0.09	31.57	64.39	24.34	66.17	45.25	41.83
272	2	92	0.43	9.09	71.57	18.71	63.35	41.03	44.64
273	2	109	0.07	15.04	113.76	25.00	45.00	35.00	20.00
274	2	130	0.07	4.50	63.47	23.09	65.80	44.45	42.71
275	2	151	0.39	15.03	60.09	7.98	67.30	37.64	59.32
276	2	174	0.09	37.56	56.61	27.61	57.83	42.72	30.22
277	2	183	0.17	36.24	56.42	37.33	51.82	44.58	14.50
278	2	204	0.27	14.21	75.26	-2.00	68.57	33.29	70.57
279	2	226	0.27	28.00	56.71	29.36	52.33	40.85	22.97

280	2	234	0.22	22.92	56.18	27.48	52.75	40.11	25.28
281	2	242	0.24	19.08	59.74	22.18	56.01	39.09	33.83
282	2	270	0.44	7.04	71.10	-4.59	68.12	31.77	72.70
283	2	298	0.28	-7.21	101.61	-7.02	77.58	35.28	84.60
284	2	323	0.06	14.71	62.88	24.61	61.75	43.18	37.14
285	2	347	0.32	20.03	53.19	23.98	48.16	36.07	24.18
286	2	362	0.18	32.23	52.58	28.66	50.00	39.33	21.34
287	2	388	0.11	10.97	79.44	23.01	56.02	39.51	33.01
288	2	395	0.26	8.55	71.63	-0.88	64.40	31.76	65.28
289	2	409	0.08	23.12	76.48	25.37	55.28	40.33	29.90
290	2	427	0.13	42.83	49.01	28.55	52.24	40.39	23.69
291	2	437	0.05	18.95	60.49	27.77	52.91	40.34	25.14
292	2	450	0.49	20.85	50.98	19.25	55.67	37.46	36.42
293	2	472	0.16	41.69	51.97	28.67	52.48	40.58	23.82
294	2	498	0.59	9.51	56.91	-2.83	61.82	29.49	64.65
295	2	509	0.12	32.14	69.42	23.63	52.22	37.93	28.59
296	2	536	0.17	31.73	53.35	17.05	52.58	34.81	35.53
297	2	545	0.57	6.55	53.67	-6.41	59.75	26.67	66.16
298	2	559	0.12	20.21	63.88	25.00	58.66	41.83	33.66
299	2	566	0.11	20.58	70.97	24.09	60.13	42.11	36.04
300	2	580	0.22	5.34	60.92	11.97	64.58	38.27	52.60
301	2	587	0.39	2.98	65.12	-4.33	65.67	30.67	70.00
302	2	621	0.42	-1.34	57.38	9.95	58.30	34.12	48.35
303	2	636	0.29	10.09	62.45	14.27	59.71	36.99	45.45
304	2	659	0.54	-7.02	71.85	-5.96	64.94	29.49	70.90
305	2	686	0.08	30.20	71.14	16.34	61.28	38.81	44.94
306	2	702	0.63	3.60	58.04	-2.41	64.36	30.98	66.78
307	2	740	0.37	-3.88	83.80	17.26	71.52	44.39	54.26
308	2	759	0.46	6.43	63.40	7.74	59.85	33.79	52.11
309	2	767	0.09	21.93	58.29	28.99	55.63	42.31	26.64
310	2	781	0.14	24.74	61.70	27.04	60.97	44.00	33.93
311	2	789	0.20	19.79	60.94	23.40	59.58	41.49	36.18
312	2	805	0.26	-3.37	79.90	5.52	61.87	33.69	56.35
313	2	839	0.08	19.76	65.03	24.21	58.63	41.42	34.41
314	2	859	0.57	15.91	56.24	17.59	58.27	37.93	40.68
315	2	880	0.26	40.94	49.63	30.46	50.00	40.23	19.54
316	2	903	0.06	33.26	59.82	32.62	58.13	45.37	25.51
317	2	913	0.59	28.76	55.23	23.07	58.36	40.71	35.30
318	2	958	0.63	18.31	52.47	13.25	52.08	32.66	38.84
319	2	1009	0.47	21.54	53.33	22.09	51.86	36.98	29.77
320	2	1024	0.08	45.96	56.61	34.95	53.23	44.09	18.28
321	2	1056	0.34	19.18	62.85	17.81	62.71	40.26	44.90
322	2	1066	0.16	20.26	54.12	25.37	57.33	41.35	31.96
323	2	1077	0.12	41.99	51.21	32.65	50.37	41.51	17.71
324	2	1084	0.13	25.11	59.44	27.52	52.70	40.11	25.17
325	2	1091	0.11	20.02	58.71	26.68	56.34	41.51	29.66
326	2	1100	0.17	14.50	68.52	17.55	58.37	37.96	40.82
327	2	1120	0.07	46.05	55.08	18.60	59.30	38.95	40.70
328	2	1130	0.46	-2.30	76.91	-9.44	66.15	28.36	75.59
329	2	1168	0.47	14.34	58.38	17.80	61.01	39.40	43.21
330	2	1198	0.30	29.47	57.41	31.95	52.22	42.08	20.28
331	2	1229	0.64	-4.40	60.87	-6.07	63.63	28.78	69.69
332	2	1251	0.45	23.78	63.65	23.44	62.02	42.73	38.58
333	2	1263	0.33	45.15	46.19	46.70	46.01	46.35	-0.69
334	2	1276	0.09	50.26	51.09	50.43	50.71	50.57	0.28
335	2	1294	0.60	-2.43	56.64	-6.30	62.69	28.19	68.99
336	2	1325	0.36	10.12	70.70	17.50	65.41	41.45	47.91
337	2	1334	0.14	9.73	67.78	24.90	61.23	43.07	36.33
338	2	1345	0.10	38.18	57.75	36.26	52.09	44.17	15.83
339	2	1374	0.44	22.94	51.28	17.86	55.15	36.50	37.29
340	2	1401	0.33	4.97	75.00	8.26	59.52	33.89	51.26
341	2	1417	0.10	36.32	57.15	22.50	49.29	35.90	26.79
342	2	1431	0.51	-7.28	73.84	-20.06	71.48	25.71	91.53

343	2	1453	0.33	31.41	55.51	24.67	52.85	38.76	28.18
344	2	1473	0.44	13.92	60.20	19.10	51.90	35.50	32.80
345	2	1488	0.52	23.34	50.19	-2.60	63.33	30.37	65.93
346	2	1502	0.14	30.40	55.21	28.64	51.03	39.84	22.40
347	2	1513	0.45	11.97	58.46	-1.96	64.74	31.39	66.71
348	2	1523	0.10	27.19	56.75	22.36	50.13	36.25	27.77
349	2	1535	0.55	9.82	60.11	12.68	58.63	35.65	45.95
350	2	1573	0.53	-18.42	64.81	-7.88	61.00	26.56	68.88
351	2	1587	0.31	6.68	81.94	-7.07	67.34	30.14	74.40
352	2	1654	0.17	50.02	51.61	33.19	63.83	48.51	30.64
353	2	1667	0.35	24.73	62.63	21.68	62.99	42.34	41.31
354	2	1692	0.49	-10.31	75.66	-1.12	64.51	31.69	65.63
355	2	1719	0.12	43.54	56.94	13.10	64.67	38.88	51.57
356	2	1734	0.52	-1.06	58.76	-10.97	64.76	26.89	75.74
357	2	1747	0.10	38.82	55.81	21.72	52.97	37.34	31.25
358	2	1758	0.11	19.06	72.42	18.83	59.73	39.28	40.90
359	2	1766	0.24	13.51	69.15	19.43	58.49	38.96	39.06
360	2	1793	0.27	-2.09	87.56	-5.18	66.59	30.70	71.77
361	2	1804	0.08	3.49	68.61	19.59	53.74	36.67	34.15
362	2	1811	0.12	14.86	60.42	21.52	58.34	39.93	36.82
363	2	1823	0.31	24.45	52.56	28.36	51.87	40.11	23.51
364	2	1843	0.30	33.68	50.67	37.33	51.37	44.35	14.04
365	2	1865	0.35	22.84	55.63	28.41	50.00	39.21	21.59
366	2	1895	0.41	25.11	55.62	31.61	55.00	43.31	23.39
367	2	1911	0.31	20.38	58.64	22.66	57.10	39.88	34.45
368	2	1930	0.29	5.32	85.11	7.41	65.15	36.28	57.74
369	2	1955	0.29	10.80	93.23	-9.29	86.88	38.79	96.16
370	2	1976	0.49	19.43	66.17	30.08	58.67	44.38	28.59
371	2	1992	0.15	33.02	57.25	28.37	52.41	40.39	24.04
372	2	2002	0.33	4.14	81.40	-0.27	66.87	33.30	67.14
373	2	2030	0.47	21.40	65.59	16.90	61.89	39.40	44.99
374	2	2070	0.48	12.57	67.29	13.32	62.52	37.92	49.21
375	2	2098	0.19	29.25	59.73	32.14	56.70	44.42	24.55
376	2	2119	0.48	21.63	56.88	20.65	59.67	40.16	39.02
377	2	2142	0.06	50.29	54.38	30.22	67.13	48.67	36.91
378	2	2155	0.35	15.39	75.42	10.77	62.68	36.72	51.91
379	2	2173	0.35	11.69	77.42	-1.60	68.83	33.61	70.43
380	2	2198	0.39	9.32	72.05	-3.97	64.46	30.25	68.43
381	2	2232	0.28	13.92	60.99	18.57	57.89	38.23	39.32
382	2	2243	0.13	12.34	57.55	18.07	63.31	40.69	45.25
383	2	2254	0.10	13.63	54.60	17.19	60.68	38.94	43.50
384	2	2271	0.29	21.21	59.28	22.85	57.86	40.35	35.01
385	2	2278	0.17	27.49	57.62	28.44	53.85	41.15	25.41
386	2	2298	0.08	38.88	62.09	33.92	57.28	45.60	23.36
387	2	2320	0.60	8.88	57.41	12.78	56.56	34.67	43.78
388	2	2351	0.20	8.62	79.01	18.79	58.18	38.49	39.39
389	2	2365	0.29	16.76	60.02	27.13	56.67	41.90	29.53
390	2	2378	0.24	38.86	52.09	27.98	50.00	38.99	22.02
391	2	2396	0.09	43.23	49.47	28.83	51.04	39.93	22.21
392	2	2410	0.29	21.78	62.51	21.00	50.72	35.86	29.72
393	2	2449	0.32	12.60	58.49	15.11	58.24	36.67	43.13
394	2	2466	0.19	41.91	50.55	27.37	50.02	38.69	22.65
395	2	2482	0.07	15.60	102.78	16.52	60.93	38.72	44.41
396	2	2498	0.38	13.68	68.46	19.54	46.52	33.03	26.98
397	2	2524	0.40	11.84	64.67	13.41	57.63	35.52	44.22
398	2	2537	0.18	8.80	57.12	17.65	62.75	40.20	45.09
399	2	2558	0.46	14.11	55.56	15.58	52.83	34.20	37.25
400	2	2595	0.50	33.40	50.05	28.11	50.92	39.52	22.80
401	2	2621	0.19	23.01	61.09	24.30	59.79	42.04	35.49
402	2	2632	0.25	25.99	57.95	23.47	60.94	42.21	37.46
403	2	2647	0.10	35.56	57.18	37.23	52.21	44.72	14.98
404	2	2655	0.15	19.90	56.24	36.65	57.00	46.83	20.34
405	2	2671	0.47	19.90	55.82	18.82	60.87	39.84	42.05

406	2	2700	0.56	9.18	54.07	15.00	54.35	34.67	39.35
407	2	2709	0.08	38.60	54.22	11.81	54.77	33.29	42.95
408	2	2721	0.06	40.08	57.86	24.54	55.27	39.91	30.73
409	2	2737	0.46	8.08	59.81	11.99	65.51	38.75	53.52
410	2	2758	0.17	42.60	53.96	16.80	61.60	39.20	44.80
411	2	2775	0.31	-8.82	99.66	8.21	65.74	36.98	57.53
412	2	2786	0.07	-9.82	101.53	11.15	62.84	37.00	51.69
413	2	2797	0.43	-5.73	74.04	15.00	32.38	23.69	17.37
414	2	2812	0.06	-2.58	150.52	9.90	72.75	41.33	62.85
415	2	2832	0.51	24.77	53.14	25.83	52.04	38.93	26.21
416	2	2856	0.05	21.41	75.61	29.49	60.65	45.07	31.16
417	2	2864	0.14	21.51	78.62	30.34	60.14	45.24	29.80
418	2	2899	0.57	17.78	54.14	15.76	59.67	37.72	43.92
419	2	2942	0.60	15.34	54.33	23.99	36.29	30.14	12.30
420	2	2968	0.40	17.97	67.68	5.00	60.83	32.92	55.83
421	2	2988	0.56	9.31	63.12	5.35	63.77	34.56	58.43
422	2	3005	0.14	33.21	60.55	32.81	60.14	46.48	27.33
423	2	3017	0.15	40.00	54.69	44.41	48.24	46.32	3.83
424	2	3025	0.29	33.98	56.90	32.08	61.57	46.82	29.48
425	2	3033	0.15	29.98	58.99	34.39	53.02	43.70	18.63
426	2	3054	0.60	9.27	53.82	14.05	41.31	27.68	27.25
427	2	3074	0.18	27.53	71.23	32.53	55.69	44.11	23.16
428	2	3085	0.10	23.04	74.18	32.04	52.46	42.25	20.41
429	2	3101	0.50	18.30	54.10	17.62	51.50	34.56	33.88
430	2	3116	0.09	15.95	64.85	26.70	60.87	43.79	34.16
431	2	3126	0.08	13.96	71.98	23.89	56.65	40.27	32.76
432	2	3137	0.36	13.80	62.85	16.80	61.28	39.04	44.48
433	2	3155	0.10	38.36	52.34	28.57	51.01	39.79	22.44
434	2	3167	0.19	15.90	62.12	18.22	61.67	39.94	43.45
435	2	3175	0.31	15.99	59.64	15.09	62.67	38.88	47.58
436	2	3197	0.10	14.17	65.12	25.39	58.76	42.08	33.37
437	2	3208	0.14	40.30	56.03	28.81	53.13	40.97	24.32
438	2	3217	0.30	18.37	63.84	21.60	54.57	38.08	32.97
439	2	3240	0.62	6.44	53.51	11.69	40.68	26.19	28.99
440	2	3263	0.35	28.81	59.88	27.98	58.69	43.33	30.71
441	2	3273	0.08	25.76	75.43	27.16	58.78	42.97	31.62
442	2	3290	0.60	10.82	46.27	14.89	44.10	29.50	29.20
443	2	3305	0.07	25.87	93.80	23.75	60.89	42.32	37.14
444	2	3336	0.58	10.72	51.42	15.00	39.28	27.14	24.28
445	2	3361	0.37	18.55	64.08	15.92	61.26	38.59	45.34
446	2	3376	0.28	36.55	53.61	36.75	52.34	44.54	15.59
447	2	3395	0.37	33.61	55.79	32.77	59.01	45.89	26.24
448	2	3424	0.64	14.60	52.49	14.41	47.33	30.87	32.91
449	2	3442	0.11	44.11	51.58	31.88	52.74	42.31	20.86
450	2	3467	0.61	11.28	57.46	-0.66	64.78	32.06	65.44
451	2	3518	0.32	13.16	69.21	7.56	62.43	35.00	54.87
452	2	3536	0.20	40.92	50.38	38.05	50.14	44.09	12.09
453	2	3554	0.59	18.87	53.87	21.86	54.69	38.27	32.83
454	2	3594	0.32	35.62	54.81	26.41	50.58	38.49	24.17
455	2	3610	0.16	17.06	54.19	20.11	59.13	39.62	39.02
456	2	3621	0.25	9.92	65.99	12.76	61.00	36.88	48.24
457	2	3629	0.17	4.79	75.43	17.23	60.48	38.86	43.25
458	2	3662	0.31	1.90	97.81	-13.66	79.59	32.97	93.26
459	2	3683	0.38	22.14	63.20	24.28	52.33	38.30	28.06
460	2	3697	0.08	38.96	66.22	29.12	56.69	42.91	27.57
461	2	3724	0.42	14.58	56.57	12.15	60.14	36.15	47.99
462	2	3752	0.06	35.10	52.74	24.50	50.85	37.68	26.35
463	2	3765	0.60	10.31	55.37	12.43	47.88	30.16	35.45
464	2	3789	0.08	3.35	132.36	21.95	57.94	39.94	35.99
465	2	3802	0.33	2.97	69.53	8.70	66.35	37.52	57.66
466	2	3832	0.45	7.85	66.13	13.81	61.69	37.75	47.88
467	2	3845	0.12	32.89	59.44	13.94	57.80	35.87	43.86
468	2	3868	0.33	14.53	67.14	-3.97	66.16	31.10	70.13

469	2	3899	0.49	22.26	57.16	25.66	53.96	39.81	28.30
470	2	3919	0.44	0.73	68.08	1.56	48.12	24.84	46.57
471	2	3944	0.34	17.14	53.89	22.62	53.56	38.09	30.94
472	2	3975	0.23	37.72	51.43	34.60	56.10	45.35	21.51
473	2	3989	0.08	31.10	51.75	43.55	49.36	46.46	5.81
474	2	3999	0.40	22.06	60.33	22.23	59.30	40.77	37.07
475	2	4024	0.44	5.80	50.88	15.00	42.44	28.72	27.44
476	2	4048	0.53	14.11	50.12	-2.28	64.29	31.01	66.56
477	2	4074	0.45	18.91	60.17	33.17	50.15	41.66	16.98
478	2	4086	0.07	27.22	66.00	28.21	58.82	43.52	30.61
479	2	4105	0.64	27.43	48.61	27.76	50.00	38.88	22.24
480	2	4146	0.61	6.46	52.48	16.73	35.76	26.24	19.03
481	2	4159	0.14	-6.66	99.27	-12.10	78.78	33.34	90.88
482	2	4176	0.05	13.52	103.54	10.88	69.36	40.12	58.48
483	2	4187	0.12	38.59	54.58	27.99	53.38	40.68	25.39
484	2	4201	0.53	-4.90	62.83	-6.11	63.37	28.63	69.48
485	2	4223	0.08	6.44	97.51	-0.16	68.41	34.12	68.57
486	2	4236	0.51	9.63	58.06	15.51	50.75	33.13	35.23
487	2	4265	0.43	27.93	53.53	32.00	51.75	41.88	19.75
488	2	4278	0.05	14.74	55.30	28.18	59.13	43.65	30.95
489	2	4293	0.44	17.33	57.36	14.92	57.89	36.41	42.97
490	2	4310	0.32	19.63	62.75	27.54	53.15	40.34	25.62
491	2	4337	0.45	9.91	61.02	23.23	59.15	41.19	35.91
492	2	4369	0.51	-1.89	60.93	5.00	65.71	35.36	60.71
493	2	4415	0.32	15.40	56.20	17.69	61.90	39.79	44.22
494	2	4463	0.54	19.45	56.78	13.10	63.48	38.29	50.38
495	2	4504	0.10	46.82	57.45	48.34	57.41	52.88	9.07
496	3	31	0.28	19.89	45.19	27.87	34.28	31.07	6.41
497	3	47	0.12	16.28	55.45	18.47	46.25	32.36	27.78
498	3	56	0.26	-2.88	53.51	28.53	34.52	31.52	6.00
499	3	64	0.37	0.73	62.53	-4.59	65.85	30.63	70.44
500	3	89	0.17	10.15	75.00	3.10	58.12	30.61	55.02
501	3	102	0.58	8.03	47.27	7.34	45.07	26.21	37.73
502	3	130	0.25	23.28	39.88	21.86	35.00	28.43	13.14
503	3	137	0.62	-4.99	55.07	-8.48	59.19	25.35	67.67
504	3	166	0.30	13.44	58.96	8.07	57.52	32.79	49.45
505	3	178	0.38	1.55	52.10	6.07	60.88	33.48	54.80
506	3	190	0.26	9.39	64.90	7.30	57.49	32.40	50.19
507	3	224	0.52	7.28	68.36	3.92	67.97	35.94	64.05
508	3	248	0.09	29.32	63.11	22.98	62.66	42.82	39.69
509	3	266	0.57	-11.36	57.02	-12.26	64.44	26.09	76.70
510	3	301	0.12	7.77	88.11	5.54	62.41	33.97	56.87
511	3	323	0.35	9.01	60.04	11.81	64.37	38.09	52.56
512	3	340	0.09	41.56	54.30	27.17	51.17	39.17	24.00
513	3	356	0.34	1.79	59.46	-2.73	62.82	30.04	65.55
514	3	383	0.57	12.34	56.31	13.05	62.36	37.70	49.31
515	3	412	0.53	13.30	52.55	-1.85	55.65	26.90	57.51
516	3	440	0.37	24.90	52.34	26.16	49.08	37.62	22.92
517	3	450	0.14	14.30	49.33	22.12	58.47	40.29	36.35
518	3	459	0.29	14.33	64.39	-6.37	68.98	31.31	75.35
519	3	492	0.59	6.47	55.43	-4.46	61.28	28.41	65.73
520	3	522	0.12	19.30	73.24	25.63	46.36	36.00	20.72
521	3	536	0.27	0.41	55.02	-3.76	53.75	24.99	57.51
522	3	552	0.53	3.21	49.96	-5.89	46.68	20.39	52.57
523	3	586	0.46	0.24	61.81	-6.56	64.86	29.15	71.43
524	3	603	0.19	9.10	59.64	23.44	53.10	38.27	29.66
525	3	616	0.38	20.48	54.51	9.95	67.30	38.63	57.35
526	3	657	0.64	5.55	53.11	-10.05	60.48	25.21	70.53
527	3	675	0.18	20.81	51.53	21.85	50.00	35.92	28.15
528	3	683	0.17	28.67	52.19	27.82	50.75	39.28	22.93
529	3	697	0.20	3.31	59.49	-1.69	63.86	31.09	65.55
530	3	705	0.42	2.18	59.20	4.27	61.92	33.09	57.65
531	3	717	0.07	24.48	57.86	26.60	51.19	38.90	24.60

532	3	746	0.50	6.40	59.89	-5.91	64.95	29.52	70.87
533	3	775	0.17	42.60	53.88	27.63	54.61	41.12	26.98
534	3	786	0.33	16.89	56.64	10.84	62.16	36.50	51.31
535	3	804	0.39	10.95	61.13	11.86	57.12	34.49	45.26
536	3	834	0.58	12.48	55.85	12.87	60.01	36.44	47.14
537	3	850	0.07	13.52	95.26	26.52	62.21	44.36	35.69
538	3	867	0.54	-0.62	59.15	-6.39	63.29	28.45	69.67
539	3	884	0.29	20.79	58.07	-1.74	58.71	28.48	60.45
540	3	897	0.25	17.06	55.89	27.57	57.89	42.73	30.32
541	3	906	0.28	20.21	58.28	27.50	51.46	39.48	23.97
542	3	922	0.50	11.58	58.94	11.47	57.68	34.57	46.21
543	3	956	0.66	7.46	60.31	-4.88	64.88	30.00	69.76
544	3	978	0.08	26.00	70.13	26.59	60.10	43.34	33.51
545	3	986	0.10	31.04	64.38	21.56	54.22	37.89	32.66
546	3	1008	0.44	10.35	55.28	12.11	56.70	34.40	44.59
547	3	1028	0.27	25.72	53.86	25.32	48.11	36.72	22.80
548	3	1053	0.53	-4.26	67.70	-4.54	63.19	29.32	67.73
549	3	1157	0.30	-4.87	78.23	9.49	63.50	36.49	54.01
550	3	1168	0.25	26.29	59.06	4.46	57.94	31.20	53.48
551	3	1180	0.32	1.89	70.79	-5.31	58.59	26.64	63.91
552	3	1208	0.25	9.78	84.56	7.00	59.08	33.04	52.08
553	3	1232	0.34	16.47	60.69	14.02	52.22	33.12	38.20
554	3	1270	0.22	14.56	61.99	13.89	60.70	37.30	46.82
555	3	1279	0.29	18.53	55.72	22.61	56.61	39.61	34.00
556	3	1291	0.06	32.32	52.98	22.79	57.85	40.32	35.06
557	3	1305	0.24	23.79	52.37	22.68	49.83	36.25	27.15
558	3	1308	0.18	19.32	54.95	24.18	50.36	37.27	26.18
559	3	1332	0.26	19.88	78.69	19.70	62.16	40.93	42.46
560	3	1358	0.63	-10.07	55.32	-11.50	55.00	21.75	66.50
561	3	1384	0.57	12.32	55.66	-0.28	59.53	29.62	59.80
562	3	1401	0.26	27.79	59.53	27.81	59.77	43.79	31.96
563	3	1420	0.10	28.35	61.46	26.15	52.26	39.21	26.11
564	3	1430	0.61	4.80	51.48	-8.73	62.87	27.07	71.60
565	3	1465	0.30	17.13	57.49	12.42	56.25	34.33	43.83
566	3	1472	0.19	8.75	52.60	19.13	56.15	37.64	37.02
567	3	1481	0.12	4.85	53.31	21.00	55.39	38.20	34.39
568	3	1484	0.11	5.45	53.80	-2.06	67.07	32.50	69.13
569	3	1492	0.20	9.69	63.73	23.05	60.12	41.59	37.07
570	3	1498	0.12	13.79	63.49	26.65	55.52	41.09	28.87
571	3	1518	0.08	17.21	74.37	23.28	55.84	39.56	32.56
572	3	1542	0.53	9.10	56.04	8.43	57.07	32.75	48.64
573	3	1565	0.20	18.27	42.73	19.22	59.64	39.43	40.42
574	3	1583	0.40	15.28	47.29	17.97	52.67	35.32	34.70
575	3	1597	0.20	23.05	62.30	27.67	53.36	40.51	25.69
576	3	1617	0.19	14.20	73.81	15.49	62.59	39.04	47.10
577	3	1628	0.59	-1.62	59.23	-1.18	55.71	27.26	56.88
578	3	1647	0.36	19.91	54.47	0.63	58.37	29.50	57.74
579	3	1658	0.14	25.21	56.34	20.92	50.83	35.88	29.91
580	3	1673	0.45	5.26	62.61	-0.14	65.66	32.76	65.79
581	3	1703	0.45	-6.89	61.16	-0.37	55.40	27.51	55.76
582	3	1733	0.31	10.67	57.37	-5.50	57.78	26.14	63.28
583	3	1749	0.30	9.17	53.11	20.88	56.01	38.44	35.13
584	3	1758	0.06	13.11	71.82	19.23	60.09	39.66	40.86
585	3	1771	0.34	9.09	59.05	18.00	54.22	36.11	36.22
586	3	1803	0.55	11.57	57.69	18.43	53.14	35.79	34.72
587	3	1820	0.07	9.30	97.05	9.89	68.48	39.19	58.59
588	3	1846	0.35	20.17	48.33	26.77	43.19	34.98	16.42
589	3	1886	0.26	1.28	64.64	10.70	55.00	32.85	44.30
590	3	1915	0.12	26.89	57.47	19.84	60.03	39.93	40.20
591	3	1922	0.07	15.74	68.90	16.92	56.07	36.49	39.15
592	3	1940	0.24	5.25	52.46	3.22	55.00	29.11	51.78
593	3	1948	0.31	5.63	54.88	17.14	54.43	35.78	37.28
594	3	1959	0.11	39.13	53.07	13.75	55.06	34.40	41.31

595	3	1989	0.41	5.47	61.19	6.84	56.68	31.76	49.84
596	3	2018	0.34	9.95	62.39	19.02	55.71	37.36	36.69
597	3	2029	0.05	3.92	67.57	13.47	66.93	40.20	53.45
598	3	2050	0.27	15.84	73.69	24.07	57.26	40.67	33.19
599	3	2073	0.07	32.19	60.39	24.33	55.24	39.79	30.92
600	3	2085	0.57	3.09	60.77	-5.53	66.56	30.52	72.10
601	3	2103	0.08	50.03	51.62	23.39	56.52	39.96	33.12
602	3	2125	0.54	1.64	66.11	0.00	60.47	30.23	60.47
603	3	2137	0.14	24.15	76.82	23.49	43.70	33.60	20.21
604	3	2173	0.46	10.23	61.64	12.07	58.36	35.22	46.30
605	3	2182	0.08	8.39	76.52	9.23	60.29	34.76	51.06
606	3	2212	0.24	10.79	64.59	15.00	55.07	35.03	40.07
607	3	2223	0.23	12.10	59.57	12.32	56.89	34.60	44.57
608	3	2243	0.16	19.22	63.49	25.81	52.43	39.12	26.62
609	3	2265	0.17	31.26	58.78	24.98	53.77	39.38	28.79
610	3	2295	0.38	13.72	61.01	12.19	53.79	32.99	41.60
611	3	2308	0.08	18.29	57.05	27.22	51.01	39.11	23.79
612	3	2318	0.13	42.79	48.21	27.08	50.00	38.54	22.92
613	3	2333	0.14	33.47	50.89	10.91	58.00	34.46	47.08
614	3	2343	0.18	15.24	55.97	17.82	52.60	35.21	34.78
615	3	2352	0.17	3.95	59.19	11.21	55.25	33.23	44.04
616	3	2373	0.13	-3.77	103.82	12.31	58.08	35.19	45.77
617	3	2395	0.09	49.65	55.80	9.48	56.50	32.99	47.01
618	3	2408	0.43	-7.84	76.66	-2.33	64.87	31.27	67.19
619	3	2428	0.45	8.39	61.43	17.40	38.17	27.78	20.77

Eshaghi_Rep2

Index	Chr	Location (kb)	Fi	Ts	Te	T0	T100	T50	DT
1	1	8	0.15	26.01	77.52	27.74	65.62	46.68	37.88
2	1	27	0.12	28.06	59.77	36.36	60.12	48.24	23.76
3	1	35	0.10	49.76	56.68	38.27	60.29	49.28	22.02
4	1	54	0.08	26.87	58.10	41.33	57.70	49.52	16.37
5	1	86	0.33	26.12	60.45	28.55	57.50	43.03	28.95
6	1	96	0.09	2.36	84.35	18.25	61.10	39.67	42.85
7	1	123	0.28	-7.37	84.75	10.39	65.56	37.98	55.17
8	1	145	0.41	10.48	71.61	-2.03	63.88	30.93	65.91
9	1	164	0.56	1.09	64.24	-10.44	58.52	24.04	68.95
10	1	181	0.08	50.32	54.43	20.00	60.23	40.11	40.23
11	1	194	0.52	4.77	55.98	9.21	55.00	32.10	45.79
12	1	213	0.19	2.26	81.24	-7.06	70.72	31.83	77.77
13	1	225	0.17	20.55	55.81	38.39	47.49	42.94	9.10
14	1	233	0.29	25.64	57.87	27.15	48.39	37.77	21.23
15	1	246	0.12	16.89	93.29	31.52	56.57	44.05	25.05
16	1	271	0.54	21.20	53.34	24.19	61.53	42.86	37.34
17	1	300	0.20	34.23	56.98	34.24	53.59	43.91	19.35
18	1	325	0.62	18.45	50.46	15.65	50.85	33.25	35.21
19	1	341	0.07	45.17	47.15	42.55	49.42	45.99	6.87
20	1	363	0.55	14.14	56.14	15.77	51.28	33.53	35.52
21	1	392	0.40	26.45	63.00	26.29	60.52	43.40	34.23
22	1	411	0.12	42.11	54.76	36.21	50.60	43.41	14.39
23	1	426	0.13	21.06	68.43	26.21	60.48	43.34	34.27
24	1	438	0.39	18.28	57.32	22.58	55.53	39.06	32.96
25	1	479	0.56	5.02	55.92	-4.99	57.83	26.42	62.82
26	1	492	0.18	13.85	62.63	15.00	53.19	34.10	38.19
27	1	503	0.20	34.79	54.93	32.89	48.35	40.62	15.46
28	1	518	0.29	29.65	56.74	28.76	52.57	40.66	23.81
29	1	532	0.54	12.49	64.93	9.85	65.65	37.75	55.80
30	1	549	0.05	44.89	52.11	27.57	50.63	39.10	23.06
31	1	565	0.61	10.47	51.15	14.90	41.70	28.30	26.80

32	1	584	0.08	43.68	53.92	34.04	51.78	42.91	17.74
33	1	610	0.35	24.41	58.52	23.99	61.56	42.77	37.57
34	1	621	0.09	42.01	57.96	28.74	57.87	43.30	29.13
35	1	650	0.39	17.58	63.83	12.12	62.71	37.41	50.59
36	1	668	0.29	25.71	57.36	23.10	58.25	40.67	35.15
37	1	689	0.25	16.22	66.49	17.71	64.00	40.86	46.29
38	1	709	0.40	18.13	58.69	22.59	38.00	30.30	15.41
39	1	752	0.64	23.92	54.05	23.86	58.76	41.31	34.90
40	1	795	0.61	21.15	53.14	15.83	58.86	37.35	43.03
41	1	816	0.09	42.05	52.18	29.82	56.77	43.29	26.95
42	1	836	0.38	-2.87	59.70	14.23	58.26	36.24	44.03
43	1	847	0.16	6.29	75.97	15.06	62.45	38.75	47.39
44	1	877	0.55	9.27	49.21	15.00	53.95	34.47	38.95
45	1	890	0.22	16.83	61.22	15.98	56.29	36.13	40.32
46	1	906	0.53	5.55	63.90	13.18	64.07	38.63	50.89
47	1	922	0.06	24.67	85.79	-8.37	77.10	34.36	85.47
48	1	931	0.52	21.99	58.01	28.06	56.17	42.12	28.11
49	1	963	0.26	15.17	55.58	25.86	44.30	35.08	18.44
50	1	972	0.21	11.58	51.02	22.82	61.35	42.09	38.52
51	1	985	0.24	17.52	58.70	22.80	50.30	36.55	27.49
52	1	1030	0.49	13.02	51.41	16.46	58.68	37.57	42.22
53	1	1044	0.15	24.10	63.39	26.66	58.26	42.46	31.59
54	1	1073	0.57	1.06	61.49	14.48	59.05	36.76	44.56
55	1	1094	0.42	1.28	58.93	11.18	36.73	23.95	25.54
56	1	1103	0.23	-1.70	57.07	8.39	46.01	27.20	37.62
57	1	1111	0.49	1.62	59.86	-2.55	62.85	30.15	65.40
58	1	1124	0.24	32.74	51.05	31.69	50.63	41.16	18.94
59	1	1147	0.34	-17.52	60.34	2.43	74.33	38.38	71.89
60	1	1182	0.14	13.12	78.70	6.10	66.62	36.36	60.52
61	1	1196	0.39	6.78	56.00	11.83	60.52	36.17	48.69
62	1	1215	0.08	42.77	49.08	0.42	63.46	31.94	63.04
63	1	1241	0.40	4.58	60.67	2.09	61.94	32.02	59.85
64	1	1256	0.08	-6.67	59.09	17.06	51.52	34.29	34.46
65	1	1265	0.20	3.05	72.60	16.21	55.00	35.61	38.79
66	1	1283	0.41	17.71	61.47	11.07	60.07	35.57	49.00
67	1	1310	0.08	38.82	52.42	35.10	50.19	42.64	15.09
68	1	1322	0.57	24.75	50.05	26.13	50.00	38.06	23.87
69	1	1353	0.18	34.32	55.24	28.16	56.29	42.22	28.13
70	1	1375	0.51	11.73	53.83	14.16	54.30	34.23	40.14
71	1	1400	0.10	10.13	88.63	17.32	61.51	39.41	44.18
72	1	1412	0.15	9.16	80.92	12.92	64.08	38.50	51.16
73	1	1428	0.27	20.41	61.56	20.00	48.42	34.21	28.43
74	1	1442	0.07	44.90	52.28	17.54	52.85	35.19	35.31
75	1	1453	0.30	-5.83	73.52	-8.96	70.39	30.72	79.34
76	1	1482	0.48	4.26	57.79	12.93	50.59	31.76	37.66
77	1	1513	0.43	5.45	57.79	3.33	56.14	29.74	52.81
78	1	1552	0.29	20.73	67.83	26.95	50.00	38.47	23.05
79	1	1571	0.19	14.99	54.86	18.24	60.05	39.15	41.81
80	1	1578	0.33	21.82	57.16	17.71	61.96	39.84	44.26
81	1	1600	0.19	38.19	50.39	37.13	55.98	46.55	18.85
82	1	1615	0.35	12.88	63.30	31.43	56.51	43.97	25.08
83	1	1625	0.16	7.71	85.82	32.14	60.98	46.56	28.84
84	1	1641	0.10	30.53	69.17	29.89	59.41	44.65	29.51
85	1	1656	0.51	16.76	55.25	-2.46	64.92	31.23	67.38
86	1	1684	0.32	1.87	72.02	-2.56	67.32	32.38	69.87
87	1	1694	0.25	18.70	56.16	22.55	47.33	34.94	24.78
88	1	1717	0.38	0.47	64.31	15.90	44.09	29.99	28.18
89	1	1737	0.41	7.87	50.58	7.29	61.03	34.16	53.75
90	1	1755	0.16	16.89	68.69	18.28	62.49	40.38	44.22
91	1	1771	0.33	15.40	64.74	17.60	66.51	42.05	48.90
92	1	1799	0.42	11.95	62.92	13.63	60.02	36.83	46.39
93	1	1813	0.08	33.15	62.50	18.93	57.65	38.29	38.72
94	1	1831	0.64	0.74	53.67	9.00	41.85	25.42	32.85

95	1	1864	0.57	7.47	57.29	3.51	53.01	28.26	49.51
96	1	1892	0.35	24.13	52.63	27.49	52.46	39.97	24.97
97	1	1903	0.29	17.35	48.49	18.52	57.55	38.03	39.02
98	1	1913	0.19	21.67	50.01	28.46	52.28	40.37	23.82
99	1	1926	0.10	23.30	67.90	25.16	58.38	41.77	33.23
100	1	1947	0.64	28.95	49.17	34.63	49.87	42.25	15.24
101	1	1977	0.65	7.30	50.31	9.31	46.33	27.82	37.01
102	1	1998	0.18	22.56	62.64	28.66	58.15	43.40	29.49
103	1	2009	0.34	23.13	61.00	37.66	59.65	48.66	21.99
104	1	2041	0.55	20.45	51.48	13.79	50.09	31.94	36.30
105	1	2070	0.28	24.49	54.46	24.78	52.39	38.58	27.61
106	1	2080	0.22	14.89	53.28	20.00	64.73	42.37	44.74
107	1	2096	0.20	13.96	67.50	22.19	60.25	41.22	38.05
108	1	2116	0.23	25.57	67.93	27.80	61.89	44.85	34.09
109	1	2135	0.37	20.26	59.77	10.40	62.86	36.63	52.45
110	1	2157	0.52	26.89	60.06	32.65	51.69	42.17	19.05
111	1	2174	0.25	19.61	64.27	23.41	47.96	35.68	24.54
112	1	2191	0.16	32.38	69.44	16.93	59.92	38.43	42.99
113	1	2211	0.42	9.25	60.92	22.71	52.93	37.82	30.23
114	1	2234	0.13	16.15	73.20	24.44	42.75	33.59	18.31
115	1	2245	0.16	22.89	70.21	16.43	55.00	35.71	38.58
116	1	2262	0.27	-7.02	84.46	16.89	51.86	34.37	34.97
117	1	2279	0.37	21.21	55.73	15.90	50.11	33.01	34.21
118	1	2288	0.13	20.19	66.46	23.44	50.46	36.95	27.02
119	1	2301	0.34	17.35	60.85	26.99	54.28	40.63	27.29
120	1	2330	0.12	34.88	58.50	32.25	55.00	43.63	22.75
121	1	2341	0.51	10.89	57.16	13.92	61.21	37.57	47.29
122	1	2368	0.53	23.29	55.05	27.86	55.95	41.91	28.09
123	1	2384	0.09	42.89	50.68	36.54	51.88	44.21	15.34
124	1	2400	0.57	19.95	57.85	22.73	52.23	37.48	29.50
125	1	2422	0.14	20.16	73.35	18.44	58.35	38.39	39.92
126	1	2442	0.59	8.89	54.02	22.21	45.00	33.61	22.79
127	1	2483	0.18	21.79	58.66	26.82	51.03	38.93	24.21
128	1	2495	0.51	17.20	50.37	15.92	59.47	37.70	43.55
129	1	2529	0.41	16.77	54.73	22.08	61.10	41.59	39.03
130	1	2547	0.19	21.01	73.25	29.39	61.56	45.48	32.16
131	1	2560	0.35	11.75	65.79	21.36	64.34	42.85	42.98
132	1	2576	0.50	19.86	50.64	15.52	47.14	31.33	31.62
133	1	2599	0.43	13.62	61.30	9.05	60.62	34.83	51.58
134	1	2616	0.36	13.41	64.14	10.71	56.85	33.78	46.13
135	1	2645	0.66	24.11	49.49	27.03	51.49	39.26	24.45
136	1	2678	0.32	21.14	65.03	27.88	58.06	42.97	30.19
137	1	2691	0.45	25.11	56.26	38.06	51.32	44.69	13.25
138	1	2704	0.13	30.17	55.60	38.42	52.27	45.35	13.85
139	1	2711	0.26	23.95	52.58	28.91	51.58	40.25	22.67
140	1	2724	0.63	4.64	52.26	1.36	50.00	25.68	48.64
141	1	2737	0.16	32.33	49.23	34.07	46.89	40.48	12.82
142	1	2754	0.45	6.13	65.41	15.44	66.35	40.90	50.90
143	1	2764	0.10	5.48	91.81	15.91	65.71	40.81	49.80
144	1	2789	0.61	5.39	50.07	13.21	45.00	29.10	31.79
145	1	2820	0.43	10.61	54.43	9.71	53.85	31.78	44.14
146	1	2835	0.29	27.39	54.15	26.70	50.92	38.81	24.22
147	1	2864	0.35	19.04	58.40	15.85	57.58	36.72	41.73
148	1	2883	0.35	25.32	54.45	28.18	50.77	39.47	22.59
149	1	2913	0.51	4.63	56.58	11.05	58.47	34.76	47.42
150	1	2936	0.13	31.59	65.25	22.72	60.06	41.39	37.33
151	1	2956	0.43	12.28	59.24	10.63	63.18	36.91	52.55
152	1	2982	0.20	-3.32	88.35	-2.23	66.91	32.34	69.15
153	1	2996	0.36	-6.88	92.44	-4.75	65.87	30.56	70.62
154	1	3011	0.57	19.95	49.91	22.27	53.09	37.68	30.83
155	1	3017	0.51	15.47	60.41	27.07	43.93	35.50	16.86
156	1	3049	0.60	-3.62	55.38	-7.27	55.00	23.87	62.27
157	1	3074	0.34	-0.13	78.28	21.87	59.64	40.75	37.77

158	1	3095	0.42	24.53	59.46	18.79	63.10	40.95	44.31
159	1	3105	0.06	41.80	55.16	32.43	50.20	41.32	17.77
160	1	3120	0.31	15.93	61.87	16.72	55.00	35.86	38.28
161	1	3127	0.24	8.71	55.18	15.96	56.40	36.18	40.45
162	1	3146	0.26	16.49	69.34	22.09	50.16	36.13	28.08
163	1	3189	0.35	16.03	55.23	22.18	55.00	38.59	32.82
164	1	3204	0.26	13.13	59.79	22.41	60.32	41.37	37.91
165	1	3232	0.13	17.30	79.13	19.15	63.21	41.18	44.06
166	1	3253	0.30	18.09	60.48	20.00	55.00	37.50	35.00
167	1	3277	0.23	5.83	68.62	12.69	60.53	36.61	47.84
168	1	3298	0.22	12.20	73.37	23.16	62.54	42.85	39.37
169	1	3311	0.17	10.40	61.75	18.59	60.16	39.37	41.56
170	1	3318	0.25	15.98	64.81	23.16	59.86	41.51	36.70
171	1	3349	0.20	24.22	58.15	30.03	50.00	40.02	19.97
172	1	3357	0.31	25.46	51.54	30.58	50.00	40.29	19.42
173	1	3369	0.33	33.30	50.61	32.73	50.47	41.60	17.74
174	1	3396	0.35	6.41	73.90	13.88	60.36	37.12	46.48
175	1	3408	0.05	40.70	53.74	27.00	53.92	40.46	26.92
176	1	3430	0.24	5.99	55.61	-0.86	61.32	30.23	62.18
177	1	3436	0.29	8.26	55.09	2.62	59.42	31.02	56.80
178	1	3446	0.13	37.10	53.53	22.45	54.89	38.67	32.45
179	1	3459	0.15	17.98	83.90	19.16	61.45	40.31	42.28
180	1	3483	0.54	7.86	58.03	-1.54	61.47	29.97	63.01
181	1	3509	0.17	14.45	68.02	13.06	56.00	34.53	42.94
182	1	3519	0.42	6.95	52.94	10.81	58.24	34.53	47.42
183	1	3545	0.21	13.56	53.36	22.61	46.21	34.41	23.60
184	1	3553	0.41	15.96	49.42	34.00	44.12	39.06	10.11
185	1	3576	0.35	6.12	72.92	-7.00	73.76	33.38	80.76
186	1	3597	0.43	8.69	61.82	-2.09	66.70	32.30	68.79
187	1	3607	0.08	37.98	53.64	21.23	55.28	38.26	34.05
188	1	3620	0.63	1.36	59.59	-18.88	69.39	25.25	88.27
189	1	3634	0.09	42.30	52.50	28.09	55.42	41.75	27.33
190	1	3651	0.13	27.08	61.60	23.21	55.12	39.17	31.91
191	1	3662	0.49	-4.83	61.02	-8.00	58.64	25.32	66.64
192	1	3691	0.13	12.39	61.75	10.00	56.58	33.29	46.58
193	1	3699	0.38	16.47	51.67	29.05	32.87	30.96	3.83
194	1	3711	0.22	8.23	65.72	8.33	59.10	33.71	50.77
195	1	3726	0.33	-21.20	93.26	0.15	58.20	29.17	58.04
196	1	3752	0.46	18.06	60.96	28.31	57.26	42.78	28.95
197	1	3803	0.64	4.63	54.17	-5.25	59.95	27.35	65.20
198	1	3820	0.27	9.95	67.97	23.28	41.55	32.42	18.27
199	1	3831	0.25	2.98	54.20	10.47	62.97	36.72	52.50
200	1	3845	0.36	12.73	58.07	22.08	55.00	38.54	32.92
201	1	3872	0.17	32.37	56.60	34.56	55.47	45.02	20.91
202	1	3889	0.59	16.08	55.83	26.69	60.04	43.36	33.35
203	1	3914	0.55	-1.10	60.36	-12.43	59.57	23.57	71.99
204	1	3926	0.15	40.69	49.12	28.01	50.00	39.00	21.99
205	1	3949	0.46	6.78	54.52	13.01	45.78	29.39	32.77
206	1	3970	0.51	-6.22	76.51	-15.19	71.37	28.09	86.57
207	1	3992	0.13	49.90	51.19	49.62	53.20	51.41	3.58
208	1	4009	0.35	6.12	85.68	-8.89	74.96	33.03	83.86
209	1	4022	0.12	16.85	72.10	7.32	60.93	34.12	53.61
210	1	4039	0.37	-20.15	68.38	-19.72	64.66	22.47	84.38
211	1	4050	0.26	-20.26	59.14	-3.72	66.71	31.50	70.42
212	1	4079	0.39	11.84	67.40	12.22	59.91	36.06	47.70
213	1	4102	0.60	-8.45	64.87	-13.29	57.75	22.23	71.04
214	1	4125	0.29	14.36	70.50	21.57	61.33	41.45	39.75
215	1	4151	0.40	4.97	62.65	8.77	64.69	36.73	55.93
216	1	4164	0.12	5.84	66.57	2.53	69.92	36.23	67.39
217	1	4192	0.12	19.50	60.67	24.45	55.58	40.01	31.13
218	1	4197	0.11	17.93	57.61	23.37	61.79	42.58	38.42
219	1	4209	0.34	16.66	57.85	27.43	57.85	42.64	30.42
220	1	4238	0.24	17.84	64.10	26.84	44.13	35.48	17.29

221	1	4261	0.44	11.80	69.34	25.85	50.53	38.19	24.69
222	1	4280	0.09	44.82	48.02	37.18	51.11	44.14	13.93
223	1	4298	0.42	11.40	59.94	16.02	57.38	36.70	41.36
224	1	4314	0.10	11.31	48.26	27.58	50.02	38.80	22.45
225	1	4324	0.44	16.32	51.17	14.39	49.07	31.73	34.68
226	1	4366	0.33	24.06	57.56	22.87	62.94	42.90	40.07
227	1	4374	0.31	18.37	54.71	23.45	57.69	40.57	34.24
228	1	4394	0.33	26.28	56.21	22.22	51.27	36.75	29.05
229	1	4415	0.56	13.78	55.41	14.87	50.00	32.44	35.12
230	1	4438	0.24	14.41	74.29	21.75	60.79	41.27	39.04
231	1	4454	0.34	11.90	72.80	17.36	62.74	40.05	45.38
232	1	4469	0.41	-0.04	69.74	-4.89	64.29	29.70	69.18
233	1	4485	0.53	13.30	55.29	14.75	45.79	30.27	31.03
234	1	4504	0.35	1.58	86.93	10.00	72.38	41.19	62.38
235	1	4528	0.55	7.74	59.98	15.37	52.37	33.87	37.00
236	1	4552	0.39	-15.23	95.72	-3.64	60.66	28.51	64.29
237	1	4562	0.27	28.56	51.00	22.52	54.11	38.31	31.59
238	1	4578	0.47	12.67	62.27	19.72	55.00	37.36	35.28
239	1	4602	0.18	30.79	57.99	31.02	53.31	42.16	22.29
240	1	4623	0.34	22.43	53.64	33.19	50.68	41.93	17.49
241	1	4640	0.35	21.27	54.17	25.46	58.42	41.94	32.96
242	1	4673	0.31	26.60	60.80	23.70	59.88	41.79	36.17
243	1	4694	0.08	22.68	61.06	43.30	50.86	47.08	7.56
244	1	4706	0.52	28.52	50.50	28.73	57.86	43.29	29.13
245	1	4721	0.20	41.99	49.89	39.72	49.72	44.72	10.00
246	1	4739	0.67	14.71	48.21	14.64	47.44	31.04	32.79
247	1	4755	0.09	38.91	52.22	33.94	50.09	42.02	16.15
248	1	4778	0.46	21.34	52.18	18.54	52.57	35.55	34.03
249	1	4791	0.18	12.65	48.54	28.05	53.35	40.70	25.30
250	1	4805	0.45	21.83	54.50	19.31	61.80	40.56	42.49
251	1	4846	0.46	12.70	53.15	23.57	50.66	37.12	27.10
252	1	4873	0.31	4.91	77.63	15.00	53.42	34.21	38.42
253	1	4890	0.14	35.77	50.11	25.64	46.73	36.18	21.09
254	1	4901	0.43	9.51	64.68	7.44	56.85	32.14	49.42
255	1	4926	0.40	4.38	63.26	10.87	44.94	27.90	34.06
256	1	4937	0.21	-1.89	58.39	12.00	52.47	32.24	40.47
257	1	4947	0.31	3.94	76.11	9.27	68.29	38.78	59.02
258	1	4975	0.60	9.03	53.74	13.48	55.27	34.37	41.80
259	1	4990	0.21	6.94	84.19	-2.01	62.67	30.33	64.69
260	1	5010	0.46	-3.34	63.60	11.35	54.56	32.95	43.21
261	1	5033	0.38	1.36	68.79	16.08	56.07	36.07	39.99
262	1	5057	0.26	6.23	72.04	16.39	58.81	37.60	42.41
263	1	5062	0.26	9.79	57.99	12.55	61.14	36.85	48.60
264	1	5071	0.28	27.66	55.16	28.30	50.01	39.15	21.70
265	1	5097	0.60	17.25	50.97	10.32	53.56	31.94	43.24
266	1	5118	0.42	19.73	57.03	23.59	50.00	36.79	26.41
267	1	5137	0.13	10.01	77.94	9.00	55.18	32.09	46.18
268	1	5145	0.21	-11.41	99.22	-16.75	89.48	36.37	106.23
269	1	5162	0.33	8.68	57.72	13.82	56.68	35.25	42.85
270	1	5174	0.24	6.41	56.62	14.13	62.52	38.33	48.39
271	1	5189	0.16	2.33	75.27	2.40	69.10	35.75	66.69
272	1	5220	0.15	30.02	63.50	28.23	58.76	43.50	30.53
273	1	5236	0.49	13.12	53.81	10.78	58.77	34.78	47.98
274	1	5266	0.32	24.11	54.50	33.52	50.23	41.87	16.71
275	1	5282	0.31	21.17	53.01	44.31	48.87	46.59	4.55
276	1	5295	0.22	25.43	50.63	30.08	51.57	40.82	21.48
277	1	5308	0.10	14.93	53.02	31.59	59.22	45.41	27.63
278	1	5320	0.30	17.62	55.96	19.46	52.27	35.87	32.82
279	1	5338	0.07	15.51	53.78	26.80	59.63	43.22	32.83
280	1	5347	0.23	16.09	53.37	20.37	60.22	40.30	39.85
281	1	5358	0.25	24.15	53.85	28.42	50.75	39.58	22.33
282	1	5370	0.12	20.84	58.94	27.16	55.49	41.32	28.33
283	1	5395	0.15	10.00	86.62	12.14	59.45	35.80	47.32

284	1	5413	0.36	2.66	65.21	-0.67	64.35	31.84	65.02
285	1	5439	0.48	5.44	59.24	12.18	46.60	29.39	34.42
286	1	5481	0.47	9.69	52.15	16.93	44.09	30.51	27.16
287	1	5509	0.11	19.16	58.59	34.76	57.34	46.05	22.58
288	1	5541	0.33	32.39	60.54	34.01	61.17	47.59	27.16
289	1	5579	0.15	27.71	59.83	27.80	65.42	46.61	37.62
290	2	7	0.11	25.18	68.95	26.09	65.89	45.99	39.80
291	2	25	0.49	25.39	58.86	26.83	60.02	43.42	33.19
292	2	91	0.32	16.31	57.90	21.18	60.64	40.91	39.47
293	2	107	0.32	16.09	63.43	25.00	62.76	43.88	37.76
294	2	144	0.06	11.09	80.83	9.40	71.05	40.22	61.65
295	2	154	0.20	5.59	69.54	16.57	59.59	38.08	43.02
296	2	165	0.42	9.48	60.01	-3.78	59.38	27.80	63.15
297	2	175	0.06	21.76	65.12	28.23	52.04	40.14	23.81
298	2	203	0.51	13.67	56.30	-3.66	67.73	32.03	71.39
299	2	234	0.23	23.28	51.98	21.36	51.54	36.45	30.18
300	2	241	0.38	16.55	45.57	21.18	50.07	35.63	28.89
301	2	256	0.09	17.50	67.52	24.04	48.26	36.15	24.22
302	2	269	0.41	1.16	72.41	-1.42	56.84	27.71	58.26
303	2	299	0.29	-5.08	85.76	-9.35	67.60	29.12	76.95
304	2	320	0.25	25.91	50.19	26.26	45.00	35.63	18.74
305	2	337	0.11	17.76	55.70	23.63	55.88	39.76	32.24
306	2	347	0.25	14.93	50.49	26.67	42.11	34.39	15.44
307	2	361	0.28	15.36	64.45	15.60	56.46	36.03	40.86
308	2	396	0.40	7.31	59.76	-0.83	65.18	32.17	66.01
309	2	408	0.24	13.01	54.11	25.00	56.71	40.86	31.71
310	2	420	0.37	20.06	57.86	22.45	60.04	41.24	37.59
311	2	450	0.22	18.85	60.77	21.68	55.00	38.34	33.32
312	2	457	0.20	17.44	50.81	21.83	60.91	41.37	39.08
313	2	464	0.22	16.84	50.95	27.67	47.00	37.34	19.34
314	2	471	0.19	24.54	55.38	30.01	51.13	40.57	21.12
315	2	493	0.29	16.36	59.92	-3.06	63.56	30.25	66.61
316	2	505	0.29	13.08	52.84	23.36	51.15	37.25	27.79
317	2	534	0.26	21.64	58.20	18.18	52.25	35.21	34.07
318	2	546	0.39	3.37	55.03	-5.85	58.08	26.11	63.93
319	2	566	0.08	0.84	50.76	25.65	57.17	41.41	31.52
320	2	583	0.58	4.77	61.46	12.86	60.61	36.73	47.75
321	2	598	0.10	49.85	51.56	18.93	67.52	43.23	48.59
322	2	625	0.66	-10.56	58.32	-12.30	50.00	18.85	62.30
323	2	639	0.13	14.94	76.86	16.69	55.14	35.91	38.45
324	2	660	0.49	-10.76	80.13	-7.86	62.54	27.34	70.39
325	2	686	0.12	24.04	61.38	14.95	55.00	34.98	40.05
326	2	701	0.48	19.07	53.26	23.98	59.03	41.51	35.05
327	2	712	0.14	19.05	54.58	22.76	55.00	38.88	32.23
328	2	737	0.43	-2.62	73.51	-4.62	63.14	29.26	67.75
329	2	758	0.28	11.39	68.46	16.18	53.77	34.97	37.59
330	2	771	0.38	15.31	56.23	22.40	54.61	38.51	32.22
331	2	787	0.11	19.30	64.82	23.33	60.07	41.70	36.74
332	2	805	0.18	-2.28	72.77	5.42	61.54	33.48	56.12
333	2	815	0.25	3.34	57.98	-0.02	71.79	35.89	71.81
334	2	841	0.33	18.90	50.98	24.49	49.12	36.80	24.63
335	2	858	0.38	17.44	59.23	19.15	63.98	41.57	44.82
336	2	874	0.10	38.42	51.04	28.24	53.17	40.71	24.93
337	2	904	0.30	36.42	49.32	31.88	50.22	41.05	18.34
338	2	916	0.31	23.77	52.01	21.42	53.72	37.57	32.30
339	2	934	0.15	18.34	71.83	27.68	58.96	43.32	31.28
340	2	957	0.42	18.51	60.66	21.31	55.67	38.49	34.36
341	2	987	0.08	33.89	68.02	32.74	59.89	46.32	27.15
342	2	1003	0.54	17.84	59.01	24.03	62.07	43.05	38.04
343	2	1030	0.51	19.61	55.37	25.81	56.56	41.19	30.74
344	2	1046	0.07	39.74	63.45	40.28	47.56	43.92	7.28
345	2	1058	0.39	20.82	56.87	16.45	55.30	35.88	38.85
346	2	1072	0.28	14.02	67.09	-1.98	67.89	32.96	69.87

347	2	1091	0.22	17.36	64.63	22.46	61.83	42.14	39.36
348	2	1109	0.29	10.01	64.49	15.00	55.00	35.00	40.00
349	2	1129	0.41	-7.38	84.29	-19.74	72.74	26.50	92.48
350	2	1143	0.16	22.05	75.86	25.00	57.31	41.16	32.31
351	2	1162	0.27	13.41	58.71	20.00	65.95	42.98	45.95
352	2	1172	0.40	15.07	57.11	17.63	55.00	36.32	37.37
353	2	1190	0.10	32.05	60.24	29.26	60.40	44.83	31.14
354	2	1204	0.43	19.72	66.43	30.33	59.54	44.93	29.22
355	2	1229	0.60	9.15	59.87	-2.13	67.17	32.52	69.31
356	2	1251	0.43	22.52	62.68	26.08	60.14	43.11	34.06
357	2	1282	0.23	29.65	64.00	14.44	60.42	37.43	45.98
358	2	1295	0.29	5.69	64.72	-9.37	72.82	31.72	82.19
359	2	1326	0.42	8.61	56.07	18.12	59.90	39.01	41.78
360	2	1330	0.33	8.78	55.98	10.22	55.00	32.61	44.78
361	2	1360	0.19	24.16	60.10	26.84	61.06	43.95	34.21
362	2	1373	0.41	17.99	47.37	27.17	46.98	37.07	19.81
363	2	1385	0.06	13.07	67.45	22.65	55.87	39.26	33.22
364	2	1405	0.59	-3.76	54.18	23.40	44.50	33.95	21.09
365	2	1431	0.66	-3.16	47.47	-25.28	65.49	20.11	90.77
366	2	1454	0.28	14.39	67.14	24.19	48.13	36.16	23.94
367	2	1473	0.40	20.83	50.13	20.55	52.01	36.28	31.46
368	2	1493	0.25	22.38	57.69	23.02	60.87	41.95	37.85
369	2	1517	0.19	12.89	77.16	27.22	50.00	38.61	22.78
370	2	1535	0.56	8.56	55.98	11.70	59.81	35.75	48.11
371	2	1550	0.10	32.02	61.97	24.25	57.09	40.67	32.84
372	2	1575	0.62	-12.71	60.59	-18.57	64.10	22.77	82.67
373	2	1587	0.32	1.88	71.49	-9.39	69.88	30.24	79.27
374	2	1597	0.29	40.16	42.99	39.72	42.38	41.05	2.66
375	2	1663	0.35	13.91	64.86	18.80	62.36	40.58	43.57
376	2	1671	0.12	3.44	58.34	8.52	70.24	39.38	61.72
377	2	1688	0.48	-11.16	67.89	-14.12	63.58	24.73	77.70
378	2	1707	0.34	29.12	56.85	11.91	55.00	33.46	43.09
379	2	1734	0.54	-5.43	56.87	-15.02	66.20	25.59	81.22
380	2	1747	0.24	29.21	50.11	26.04	43.22	34.63	17.18
381	2	1764	0.60	12.22	53.00	14.89	52.81	33.85	37.92
382	2	1793	0.50	-2.37	67.38	-6.37	67.58	30.61	73.95
383	2	1812	0.31	19.86	51.08	28.25	51.64	39.94	23.39
384	2	1823	0.32	22.92	54.60	28.49	52.26	40.37	23.77
385	2	1843	0.28	24.47	57.35	38.70	47.88	43.29	9.17
386	2	1866	0.21	18.92	57.47	-2.12	68.07	32.97	70.19
387	2	1896	0.51	18.48	54.49	27.74	59.07	43.41	31.33
388	2	1909	0.18	11.86	55.86	21.68	60.00	40.84	38.33
389	2	1926	0.32	3.80	72.44	-17.83	65.98	24.08	83.81
390	2	1955	0.38	13.87	56.85	-10.26	72.04	30.89	82.30
391	2	1975	0.67	7.07	53.39	0.38	58.15	29.27	57.77
392	2	1992	0.17	28.64	51.94	26.88	52.26	39.57	25.38
393	2	2007	0.31	-13.88	82.26	-7.81	70.12	31.16	77.93
394	2	2026	0.46	17.55	54.18	17.66	55.00	36.33	37.34
395	2	2048	0.22	5.48	76.24	-9.64	77.07	33.72	86.71
396	2	2056	0.41	4.51	59.75	23.71	46.84	35.28	23.13
397	2	2072	0.61	10.14	52.66	23.03	43.73	33.38	20.70
398	2	2093	0.38	24.14	63.02	22.58	62.64	42.61	40.06
399	2	2112	0.21	16.51	54.80	31.50	59.65	45.57	28.15
400	2	2122	0.43	19.89	59.27	20.80	57.17	38.99	36.36
401	2	2159	0.38	12.03	70.61	8.72	63.08	35.90	54.36
402	2	2175	0.26	17.38	67.10	23.23	60.79	42.01	37.55
403	2	2198	0.21	-2.16	93.38	-7.13	71.78	32.32	78.91
404	2	2210	0.18	-0.28	95.47	-3.66	63.09	29.71	66.76
405	2	2232	0.42	14.97	54.53	21.16	52.46	36.81	31.31
406	2	2244	0.21	10.89	64.06	18.14	66.22	42.18	48.08
407	2	2269	0.33	13.97	67.47	-3.94	66.15	31.11	70.09
408	2	2279	0.16	14.22	61.96	26.82	59.18	43.00	32.36
409	2	2290	0.18	20.37	62.30	31.98	56.91	44.44	24.93

410	2	2299	0.33	5.44	69.59	-1.43	70.59	34.58	72.02
411	2	2319	0.35	10.38	60.80	19.38	58.85	39.11	39.47
412	2	2352	0.23	12.46	78.29	19.83	63.09	41.46	43.26
413	2	2374	0.43	20.04	54.20	18.20	57.11	37.65	38.91
414	2	2412	0.52	19.37	53.36	21.64	48.85	35.25	27.21
415	2	2448	0.53	8.26	55.93	13.86	57.37	35.61	43.51
416	2	2484	0.33	17.86	67.12	24.28	51.81	38.04	27.53
417	2	2498	0.44	11.19	60.58	17.64	45.85	31.75	28.21
418	2	2521	0.42	10.49	61.99	13.35	57.62	35.48	44.27
419	2	2537	0.10	6.73	56.05	24.46	59.31	41.89	34.86
420	2	2554	0.54	14.71	52.64	13.92	50.00	31.96	36.08
421	2	2594	0.49	25.76	57.52	23.34	58.42	40.88	35.07
422	2	2621	0.25	34.89	52.87	32.51	58.26	45.38	25.75
423	2	2640	0.11	28.96	57.77	45.21	52.73	48.97	7.52
424	2	2646	0.15	33.53	59.30	37.11	52.96	45.03	15.85
425	2	2671	0.58	18.40	51.73	22.59	51.55	37.07	28.96
426	2	2700	0.55	3.42	57.78	9.99	61.11	35.55	51.12
427	2	2728	0.21	9.65	74.13	12.95	57.93	35.44	44.98
428	2	2739	0.58	1.86	49.94	-6.41	51.27	22.43	57.69
429	2	2760	0.31	9.76	88.61	-6.93	80.30	36.69	87.22
430	2	2786	0.30	-1.15	88.45	12.68	57.34	35.01	44.66
431	2	2801	0.44	0.26	60.17	15.00	55.00	35.00	40.00
432	2	2811	0.12	1.42	95.31	3.49	80.58	42.03	77.10
433	2	2832	0.45	24.48	54.43	25.86	50.72	38.29	24.87
434	2	2847	0.33	27.32	57.80	38.34	48.38	43.36	10.04
435	2	2856	0.13	22.74	56.32	34.74	59.04	46.89	24.30
436	2	2870	0.26	33.11	55.76	37.97	50.34	44.15	12.36
437	2	2885	0.13	21.21	57.04	28.25	61.06	44.66	32.81
438	2	2899	0.24	15.12	51.82	17.09	63.24	40.16	46.15
439	2	2908	0.35	21.05	55.28	25.57	47.87	36.72	22.29
440	2	2943	0.55	12.45	47.89	21.75	42.14	31.95	20.39
441	2	2970	0.11	20.72	88.12	32.44	66.06	49.25	33.63
442	2	2977	0.17	34.90	51.68	34.75	49.08	41.91	14.33
443	2	2989	0.69	0.07	51.61	-9.02	42.84	16.91	51.86
444	2	3007	0.23	31.75	56.06	27.72	56.37	42.04	28.65
445	2	3017	0.24	30.89	52.03	38.77	51.80	45.28	13.03
446	2	3025	0.31	25.60	52.84	28.33	58.28	43.30	29.95
447	2	3033	0.38	21.63	56.69	20.69	57.21	38.95	36.51
448	2	3054	0.66	12.51	51.53	12.72	43.35	28.03	30.63
449	2	3074	0.53	17.01	60.03	24.37	55.50	39.94	31.13
450	2	3084	0.18	22.28	69.07	24.68	59.24	41.96	34.56
451	2	3101	0.59	12.41	52.70	3.03	50.00	26.51	46.97
452	2	3115	0.22	22.01	59.78	24.08	53.49	38.78	29.41
453	2	3125	0.21	13.56	72.62	24.00	63.50	43.75	39.49
454	2	3139	0.14	15.84	75.67	19.11	65.22	42.16	46.11
455	2	3175	0.31	12.44	61.66	14.51	61.31	37.91	46.80
456	2	3185	0.19	13.81	55.29	17.75	61.15	39.45	43.40
457	2	3195	0.19	37.95	54.96	24.19	57.18	40.68	32.99
458	2	3220	0.08	18.80	88.23	24.82	61.39	43.11	36.56
459	2	3240	0.53	2.63	50.27	11.27	44.48	27.88	33.22
460	2	3250	0.16	9.24	74.81	16.75	60.23	38.49	43.48
461	2	3262	0.16	28.29	69.24	26.20	60.98	43.59	34.78
462	2	3289	0.68	18.78	55.19	16.20	61.93	39.06	45.73
463	2	3304	0.30	25.42	55.95	22.81	56.91	39.86	34.10
464	2	3326	0.32	-12.02	72.88	-2.84	70.94	34.05	73.79
465	2	3335	0.18	18.09	60.55	15.95	40.65	28.30	24.69
466	2	3342	0.14	8.56	49.43	13.14	61.74	37.44	48.60
467	2	3360	0.41	20.38	50.71	22.14	53.83	37.98	31.68
468	2	3396	0.26	30.94	60.12	32.25	60.36	46.30	28.11
469	2	3424	0.66	14.34	56.81	4.65	65.58	35.11	60.92
470	2	3452	0.20	24.28	64.26	25.00	50.38	37.69	25.38
471	2	3465	0.46	2.03	64.45	5.17	58.46	31.81	53.30
472	2	3485	0.06	21.81	95.93	27.46	55.00	41.23	27.54

473	2	3504	0.39	18.45	54.77	23.51	56.11	39.81	32.60
474	2	3518	0.17	13.41	99.56	14.48	65.60	40.04	51.11
475	2	3540	0.28	25.49	59.95	28.35	55.91	42.13	27.56
476	2	3560	0.37	15.97	54.58	12.59	59.19	35.89	46.59
477	2	3594	0.27	28.82	55.97	19.60	53.22	36.41	33.62
478	2	3607	0.36	12.94	54.28	19.27	56.76	38.02	37.49
479	2	3618	0.14	10.41	72.63	17.36	62.66	40.01	45.30
480	2	3636	0.37	13.39	60.67	10.79	63.20	36.99	52.41
481	2	3655	0.17	30.01	54.05	26.74	44.60	35.67	17.86
482	2	3664	0.46	2.50	67.33	-19.11	74.23	27.56	93.34
483	2	3686	0.38	19.03	57.18	12.10	57.66	34.88	45.56
484	2	3706	0.19	35.75	52.30	34.36	52.17	43.27	17.81
485	2	3723	0.49	9.54	59.82	14.93	59.23	37.08	44.30
486	2	3735	0.21	17.89	68.10	21.90	61.06	41.48	39.15
487	2	3765	0.69	10.56	57.76	14.09	47.93	31.01	33.84
488	2	3786	0.23	40.53	51.01	23.90	55.00	39.45	31.10
489	2	3806	0.47	5.61	60.65	15.00	45.32	30.16	30.32
490	2	3826	0.32	6.22	52.29	11.22	57.19	34.21	45.97
491	2	3834	0.30	5.70	54.52	15.10	57.13	36.11	42.03
492	2	3846	0.10	13.09	70.66	18.47	59.85	39.16	41.38
493	2	3859	0.22	6.49	56.44	-4.72	66.10	30.69	70.82
494	2	3868	0.32	12.61	61.00	-4.58	67.35	31.39	71.93
495	2	3901	0.46	18.28	57.99	16.79	58.60	37.69	41.81
496	2	3923	0.42	-4.33	63.88	12.42	50.38	31.40	37.95
497	2	3937	0.19	3.47	72.61	16.66	66.93	41.79	50.27
498	2	3951	0.44	13.55	56.35	23.35	52.69	38.02	29.33
499	2	3978	0.29	34.08	52.25	42.38	50.31	46.35	7.94
500	2	3989	0.27	24.45	49.94	41.86	49.67	45.76	7.81
501	2	3998	0.40	20.93	53.74	22.30	50.68	36.49	28.38
502	2	4029	0.60	5.95	45.86	27.90	34.28	31.09	6.38
503	2	4051	0.59	16.25	43.54	-3.16	54.45	25.65	57.61
504	2	4073	0.50	7.35	62.54	-5.38	68.79	31.71	74.17
505	2	4100	0.09	42.68	50.77	37.02	51.79	44.41	14.77
506	2	4115	0.51	25.34	55.46	24.50	59.62	42.06	35.12
507	2	4123	0.14	20.81	60.14	26.44	52.09	39.26	25.64
508	2	4146	0.48	4.05	54.16	17.40	35.12	26.26	17.72
509	2	4156	0.14	-4.36	102.04	-5.55	68.57	31.51	74.12
510	2	4177	0.36	9.21	69.22	10.26	63.30	36.78	53.04
511	2	4193	0.13	-3.23	76.83	-10.94	65.21	27.14	76.16
512	2	4201	0.48	4.27	52.67	11.81	57.59	34.70	45.78
513	2	4224	0.25	0.95	84.43	-7.07	63.88	28.41	70.95
514	2	4239	0.38	6.06	68.69	1.88	58.92	30.40	57.04
515	2	4256	0.07	24.51	65.92	28.83	50.00	39.41	21.17
516	2	4265	0.35	26.33	49.34	28.95	50.00	39.47	21.05
517	2	4297	0.36	19.66	57.08	22.70	51.59	37.14	28.89
518	2	4315	0.37	28.50	53.72	27.88	50.39	39.14	22.52
519	2	4343	0.30	21.98	57.78	23.85	48.05	35.95	24.21
520	2	4353	0.24	-2.32	75.80	-5.15	68.37	31.61	73.52
521	2	4362	0.34	13.56	60.21	10.63	46.87	28.75	36.24
522	2	4373	0.22	1.36	96.14	6.31	67.68	37.00	61.37
523	2	4404	0.09	38.89	59.64	25.00	53.68	39.34	28.68
524	2	4414	0.22	19.35	55.93	16.34	61.32	38.83	44.98
525	2	4428	0.21	16.70	55.57	23.55	60.32	41.93	36.77
526	2	4455	0.08	16.82	69.40	23.69	70.14	46.91	46.45
527	2	4471	0.16	18.41	69.49	24.77	61.79	43.28	37.02
528	2	4504	0.18	49.55	57.98	33.50	63.91	48.71	30.41
529	3	45	0.39	19.00	38.23	8.39	51.54	29.97	43.14
530	3	55	0.22	8.66	47.40	28.23	34.33	31.28	6.10
531	3	68	0.19	-1.19	77.86	-3.42	69.80	33.19	73.22
532	3	92	0.37	6.81	52.23	0.05	54.77	27.41	54.72
533	3	107	0.50	10.31	45.48	24.00	34.87	29.44	10.87
534	3	125	0.40	18.83	37.61	-15.65	55.00	19.68	70.65
535	3	142	0.67	-13.53	50.92	-7.60	50.00	21.20	57.60

536	3	166	0.14	25.93	53.59	22.74	51.57	37.16	28.82
537	3	183	0.16	19.06	56.77	10.70	56.05	33.38	45.35
538	3	189	0.20	5.99	51.50	9.16	56.30	32.73	47.14
539	3	202	0.35	-0.69	57.21	14.14	55.00	34.57	40.86
540	3	214	0.05	-6.79	92.62	-0.80	65.05	32.13	65.85
541	3	237	0.16	9.39	74.36	10.42	65.49	37.96	55.07
542	3	267	0.57	-10.89	64.12	-11.59	63.76	26.08	75.35
543	3	302	0.08	5.99	99.29	5.41	67.22	36.32	61.81
544	3	321	0.28	18.35	51.88	9.91	54.56	32.24	44.65
545	3	339	0.06	-1.78	47.43	21.19	56.15	38.67	34.96
546	3	352	0.19	4.11	60.82	-3.81	64.70	30.44	68.50
547	3	382	0.37	12.58	61.62	-4.51	61.56	28.52	66.07
548	3	412	0.61	7.73	57.25	-2.62	67.02	32.20	69.64
549	3	444	0.42	8.34	57.09	-2.71	57.42	27.35	60.13
550	3	458	0.31	3.03	69.93	-7.58	64.42	28.42	72.00
551	3	492	0.57	0.77	48.41	-8.55	55.00	23.22	63.55
552	3	514	0.26	36.73	45.11	-15.06	65.68	25.31	80.74
553	3	537	0.42	-1.41	50.20	-3.27	58.69	27.71	61.97
554	3	554	0.33	0.81	77.08	10.00	60.92	35.46	50.92
555	3	570	0.09	21.19	75.72	6.10	59.42	32.76	53.32
556	3	590	0.48	2.74	55.78	8.70	60.31	34.51	51.61
557	3	623	0.42	-2.17	66.54	-8.04	69.32	30.64	77.36
558	3	636	0.12	4.83	62.33	21.02	59.90	40.46	38.88
559	3	656	0.36	6.62	56.97	17.03	57.00	37.02	39.96
560	3	668	0.23	13.43	62.17	17.56	56.04	36.80	38.49
561	3	674	0.13	26.02	55.34	26.43	50.82	38.63	24.39
562	3	697	0.48	-3.36	64.78	-6.14	67.40	30.63	73.54
563	3	717	0.16	23.48	58.00	23.79	55.59	39.69	31.80
564	3	735	0.40	-0.83	63.55	-3.33	60.36	28.52	63.69
565	3	747	0.22	-0.24	61.90	-2.53	73.22	35.35	75.75
566	3	758	0.31	27.40	52.62	24.47	54.70	39.59	30.23
567	3	766	0.08	28.66	54.82	34.01	47.22	40.61	13.21
568	3	786	0.44	4.64	59.07	-1.78	55.00	26.61	56.79
569	3	796	0.08	-3.02	58.53	-3.97	61.31	28.67	65.29
570	3	804	0.32	10.64	65.46	19.37	59.43	39.40	40.06
571	3	818	0.15	22.22	61.44	23.78	60.22	42.00	36.44
572	3	827	0.22	20.37	62.36	33.92	50.00	41.96	16.07
573	3	850	0.30	22.95	49.43	26.38	44.89	35.64	18.52
574	3	867	0.34	2.37	60.21	1.02	63.46	32.24	62.44
575	3	884	0.20	29.19	54.46	-3.14	61.67	29.26	64.81
576	3	893	0.27	17.28	60.83	18.12	57.11	37.62	39.00
577	3	910	0.47	15.19	54.99	29.93	43.18	36.56	13.25
578	3	930	0.20	20.40	66.32	22.77	57.61	40.19	34.84
579	3	956	0.68	1.43	55.28	-10.14	65.65	27.75	75.80
580	3	1002	0.35	10.94	59.67	1.63	61.25	31.44	59.62
581	3	1007	0.32	13.15	51.07	19.10	54.90	37.00	35.80
582	3	1017	0.15	25.95	60.27	23.60	56.23	39.92	32.63
583	3	1050	0.59	-8.71	65.33	-12.52	74.45	30.96	86.97
584	3	1153	0.58	-2.22	66.35	-7.76	68.04	30.14	75.80
585	3	1165	0.07	50.45	53.78	28.99	66.68	47.84	37.69
586	3	1181	0.40	0.53	65.11	-4.54	55.46	25.46	60.00
587	3	1193	0.16	28.67	53.53	21.76	45.00	33.38	23.24
588	3	1213	0.47	5.60	59.90	-5.48	64.34	29.43	69.82
589	3	1233	0.21	16.98	65.48	18.96	56.00	37.48	37.03
590	3	1270	0.56	7.22	53.41	12.87	55.73	34.30	42.86
591	3	1302	0.35	29.16	38.98	-0.67	50.00	24.67	50.67
592	3	1310	0.47	-7.71	76.79	-6.94	62.80	27.93	69.74
593	3	1333	0.50	20.21	53.38	22.10	56.51	39.30	34.42
594	3	1345	0.12	34.19	59.42	26.65	55.00	40.82	28.35
595	3	1356	0.59	-10.81	57.18	-5.43	52.06	23.31	57.49
596	3	1388	0.54	10.91	54.77	11.26	63.07	37.16	51.81
597	3	1401	0.16	22.31	64.04	20.17	60.16	40.16	39.99
598	3	1421	0.07	35.19	61.00	27.04	57.75	42.40	30.71

599	3	1432	0.59	0.97	51.44	-4.43	62.10	28.84	66.53
600	3	1465	0.28	16.86	56.65	18.07	61.17	39.62	43.10
601	3	1473	0.28	8.07	50.83	18.19	54.53	36.36	36.34
602	3	1490	0.18	33.68	45.01	32.77	39.73	36.25	6.96
603	3	1513	0.36	-3.91	70.73	5.82	59.21	32.51	53.39
604	3	1550	0.33	14.19	51.94	25.25	51.77	38.51	26.52
605	3	1565	0.14	13.75	53.05	24.52	45.00	34.76	20.48
606	3	1583	0.14	13.09	74.11	16.78	55.87	36.32	39.10
607	3	1599	0.48	22.24	54.27	23.80	50.52	37.16	26.71
608	3	1615	0.11	37.04	54.83	21.18	55.00	38.09	33.82
609	3	1626	0.47	-3.19	71.27	-7.01	60.77	26.88	67.78
610	3	1649	0.57	-0.67	65.04	-8.45	65.59	28.57	74.03
611	3	1667	0.43	11.65	52.82	-2.67	55.00	26.16	57.67
612	3	1702	0.68	9.42	49.44	11.58	45.32	28.45	33.74
613	3	1737	0.60	7.78	56.60	10.42	50.72	30.57	40.29
614	3	1749	0.25	18.73	58.13	21.26	53.06	37.16	31.80
615	3	1757	0.07	31.62	53.80	22.27	55.00	38.64	32.73
616	3	1772	0.53	4.81	55.76	12.39	58.29	35.34	45.90
617	3	1789	0.11	45.73	48.46	31.47	51.21	41.34	19.74
618	3	1805	0.56	11.65	58.18	9.96	56.88	33.42	46.92
619	3	1848	0.48	16.62	54.50	17.31	55.00	36.15	37.69
620	3	1858	0.11	6.05	55.25	23.36	60.40	41.88	37.04
621	3	1882	0.31	-0.77	75.19	12.76	55.89	34.32	43.13
622	3	1915	0.64	15.71	52.73	19.94	50.36	35.15	30.43
623	3	1950	0.26	11.38	68.09	10.83	58.45	34.64	47.62
624	3	1985	0.32	6.48	57.67	7.47	58.10	32.78	50.63
625	3	2006	0.06	12.61	55.88	21.52	54.18	37.85	32.66
626	3	2015	0.48	5.92	58.83	-4.78	56.86	26.04	61.65
627	3	2038	0.27	21.42	62.10	23.98	56.52	40.25	32.54
628	3	2059	0.45	9.03	57.91	-0.95	71.32	35.18	72.27
629	3	2072	0.19	5.67	58.47	20.23	55.00	37.62	34.77
630	3	2083	0.20	-0.23	82.83	-7.72	63.34	27.81	71.06
631	3	2096	0.35	-1.18	79.77	-10.51	55.00	22.25	65.51
632	3	2110	0.26	20.58	56.01	29.46	42.80	36.13	13.34
633	3	2119	0.10	8.52	71.70	12.66	64.05	38.36	51.38
634	3	2128	0.36	7.34	65.21	8.76	57.57	33.16	48.81
635	3	2165	0.58	12.53	57.54	20.74	58.53	39.63	37.79
636	3	2194	0.09	28.44	65.32	22.49	53.79	38.14	31.30
637	3	2202	0.25	15.82	70.96	26.79	40.50	33.64	13.71
638	3	2217	0.26	12.13	67.43	21.33	52.35	36.84	31.02
639	3	2226	0.11	9.42	65.51	23.13	60.49	41.81	37.36
640	3	2245	0.40	11.77	58.69	21.89	51.74	36.82	29.85
641	3	2270	0.21	14.98	64.94	18.63	55.68	37.16	37.05
642	3	2284	0.17	13.39	59.36	24.86	54.23	39.54	29.37
643	3	2308	0.24	14.96	56.86	24.48	56.39	40.43	31.92
644	3	2330	0.22	14.91	59.00	18.14	53.34	35.74	35.20
645	3	2345	0.30	16.53	61.91	27.87	37.03	32.45	9.16
646	3	2370	0.38	1.04	77.27	8.21	60.32	34.26	52.11
647	3	2397	0.07	22.82	60.30	6.63	65.17	35.90	58.54
648	3	2407	0.38	-2.20	69.57	9.55	55.84	32.69	46.29
649	3	2416	0.14	-3.50	53.71	18.80	56.32	37.56	37.52
650	3	2428	0.39	16.39	53.65	19.69	45.50	32.59	25.81

Human Index	Chr	Location (Mb)	Fi	Ts	Te	T0	T100	T50	DT
1	21	13.372	0.61	5.09	10.65	1.53	8.00	4.77	6.47
2	21	14.002	0.34	5.35	8.48	1.79	8.74	5.27	6.95
3	21	14.14	0.44	6.31	7.79	1.09	7.21	4.15	6.12
4	21	14.182	0.43	5.39	11.03	0.74	4.47	2.60	3.73
5	21	14.242	0.42	-0.75	12.08	1.16	7.81	4.49	6.65

6	21	14.482	0.10	7.76	9.20	1.11	8.73	4.92	7.62
7	21	14.62	0.11	7.89	10.12	1.14	15.02	8.08	13.89
8	21	14.854	0.19	7.46	10.49	1.36	8.65	5.01	7.30
9	21	15.1	0.41	6.58	10.69	0.89	10.35	5.62	9.45
10	21	15.388	0.22	3.30	15.15	1.03	9.90	5.47	8.87
11	21	15.502	0.28	4.81	13.90	1.24	9.38	5.31	8.14
12	21	15.616	0.16	6.62	12.37	1.18	8.23	4.70	7.06
13	21	15.832	0.46	7.36	9.44	1.11	8.54	4.82	7.43
14	21	16.078	0.18	8.14	9.57	1.06	9.93	5.50	8.87
15	21	16.198	0.15	8.00	12.27	0.98	10.74	5.86	9.77
16	21	16.456	0.39	2.35	15.07	1.04	9.94	5.49	8.90
17	21	16.732	0.18	7.70	12.97	1.43	9.24	5.34	7.82
18	21	17.032	0.41	5.12	14.26	1.09	8.65	4.87	7.56
19	21	17.26	0.13	7.80	13.57	0.98	9.55	5.26	8.57
20	21	17.374	0.14	7.36	12.17	1.05	10.04	5.54	8.99
21	21	17.734	0.48	7.16	9.89	1.07	8.15	4.61	7.08
22	21	17.98	0.19	7.07	10.38	1.50	9.87	5.68	8.37
23	21	18.34	0.43	5.73	13.94	1.37	10.24	5.81	8.86
24	21	18.604	0.14	7.91	14.61	0.38	16.95	8.66	16.57
25	21	19.054	0.34	-0.28	14.82	1.26	11.16	6.21	9.90
26	21	19.552	0.36	7.15	12.33	1.30	9.41	5.36	8.11
27	21	19.81	0.34	7.08	10.80	1.44	9.71	5.57	8.27
28	21	20.14	0.35	7.15	12.41	1.19	9.77	5.48	8.58
29	21	20.434	0.21	7.81	11.30	1.20	10.02	5.61	8.82
30	21	20.56	0.23	-0.53	14.34	1.16	11.57	6.36	10.41
31	21	20.692	0.39	7.05	11.22	1.47	10.00	5.73	8.53
32	21	20.764	0.36	6.80	13.63	1.03	10.65	5.84	9.62
33	21	20.926	0.27	-0.32	13.38	1.21	9.20	5.20	7.98
34	21	21.064	0.24	8.21	12.53	1.21	10.24	5.72	9.03
35	21	21.202	0.30	7.28	11.18	1.21	8.73	4.97	7.52
36	21	21.388	0.31	7.01	12.78	0.99	10.35	5.67	9.36
37	21	21.502	0.35	7.38	13.32	1.25	10.32	5.79	9.07
38	21	21.64	0.17	8.08	10.68	2.11	13.75	7.93	11.63
39	21	21.856	0.28	7.62	9.80	1.09	9.81	5.45	8.71
40	21	21.964	0.33	4.68	13.60	1.34	10.00	5.67	8.66
41	21	22.24	0.37	7.16	9.84	1.20	8.96	5.08	7.76
42	21	22.57	0.31	6.86	14.56	1.04	9.12	5.08	8.09
43	21	22.672	0.27	5.65	15.15	1.28	11.55	6.42	10.27
44	21	22.834	0.21	8.03	11.60	1.19	10.19	5.69	9.00
45	21	22.984	0.30	7.17	10.76	1.27	11.58	6.42	10.31
46	21	23.086	0.28	7.65	10.55	1.31	8.49	4.90	7.18
47	21	23.272	0.35	0.38	12.98	1.52	10.58	6.05	9.06
48	21	23.488	0.18	6.88	14.37	1.31	9.24	5.27	7.93
49	21	23.728	0.35	7.27	9.64	1.22	9.22	5.22	8.00
50	21	24.154	0.35	6.42	12.13	1.26	8.34	4.80	7.08
51	21	24.28	0.40	5.52	12.92	1.14	10.10	5.62	8.96
52	21	24.562	0.27	5.77	11.42	1.41	9.29	5.35	7.88
53	21	24.922	0.40	7.00	10.76	1.58	11.00	6.29	9.42
54	21	25.048	0.41	7.30	11.72	1.18	9.77	5.47	8.59
55	21	25.174	0.30	7.39	15.82	1.11	9.94	5.53	8.83
56	21	25.468	0.20	7.07	10.30	1.40	8.19	4.79	6.79
57	21	25.726	0.48	-0.23	14.06	1.28	8.37	4.83	7.09
58	21	26.02	0.18	7.69	9.40	1.30	9.80	5.55	8.50
59	21	26.266	0.43	6.44	10.76	1.25	8.76	5.00	7.51
60	21	26.872	0.35	6.85	10.80	1.35	8.80	5.07	7.45
61	21	26.986	0.31	6.18	14.94	1.21	8.16	4.68	6.95
62	21	27.172	0.34	6.93	11.29	1.31	9.08	5.20	7.77
63	21	27.286	0.26	7.39	10.48	1.14	8.98	5.06	7.84
64	21	27.46	0.24	7.84	11.55	0.95	9.81	5.38	8.86
65	21	27.598	0.25	8.12	13.58	1.19	9.24	5.22	8.05
66	21	27.736	0.25	7.25	10.80	0.99	8.98	4.99	7.99
67	21	27.802	0.25	7.28	11.90	1.27	8.45	4.86	7.17
68	21	27.964	0.28	0.01	14.79	1.31	9.43	5.37	8.12

69	21	28.078	0.43	1.96	15.54	0.93	9.17	5.05	8.24
70	21	28.198	0.30	8.26	11.05	1.58	9.65	5.62	8.07
71	21	28.36	0.18	7.88	8.85	1.30	9.59	5.44	8.28
72	21	28.51	0.18	5.92	15.28	1.17	9.35	5.26	8.18
73	21	28.702	0.43	4.84	13.08	1.35	8.31	4.83	6.97
74	21	28.816	0.35	8.09	12.07	1.28	9.70	5.49	8.42
75	21	28.972	0.30	6.95	10.55	1.19	8.84	5.02	7.65
76	21	29.386	0.42	7.06	11.09	0.88	9.57	5.22	8.69
77	21	29.584	0.38	7.11	12.20	1.03	8.86	4.94	7.83
78	21	29.908	0.25	7.58	10.21	1.28	9.73	5.50	8.45
79	21	30.244	0.21	6.43	15.38	1.23	10.03	5.63	8.80
80	21	30.412	0.30	6.20	12.56	1.32	10.63	5.98	9.31
81	21	30.514	0.41	7.06	13.29	1.27	10.23	5.75	8.96
82	21	30.832	0.17	7.52	10.34	1.33	9.22	5.28	7.89
83	21	30.97	0.20	0.08	18.73	1.34	9.07	5.21	7.73
84	21	31.15	0.43	6.65	12.80	0.63	9.88	5.25	9.25
85	21	31.732	0.47	5.46	12.18	1.18	8.06	4.62	6.88
86	21	32.14	0.16	7.30	13.00	1.22	8.83	5.02	7.61
87	21	32.362	0.28	2.12	13.68	1.08	8.14	4.61	7.07
88	21	32.452	0.28	6.43	11.30	1.28	9.69	5.48	8.41
89	21	32.746	0.42	5.77	9.93	1.03	8.54	4.78	7.51
90	21	32.788	0.39	2.23	11.88	1.03	8.48	4.75	7.44
91	21	33.07	0.14	6.98	15.13	1.22	10.11	5.67	8.89
92	21	33.46	0.39	5.25	10.36	1.17	7.49	4.33	6.32
93	21	33.556	0.43	2.30	11.64	1.12	7.53	4.32	6.41
94	21	34.204	0.54	4.94	11.89	0.95	8.82	4.89	7.87
95	21	34.738	0.37	4.98	12.46	1.16	9.13	5.15	7.97
96	21	34.84	0.30	-0.35	12.00	1.12	7.68	4.40	6.55
97	21	34.888	0.31	5.79	8.41	1.31	8.64	4.98	7.33
98	21	35.056	0.38	4.49	12.00	0.67	9.44	5.05	8.76
99	21	35.2	0.24	6.91	10.43	1.18	7.65	4.42	6.48
100	21	35.374	0.35	-0.54	13.17	1.14	10.72	5.93	9.59
101	21	35.68	0.25	7.15	8.84	1.07	9.37	5.22	8.30
102	21	36.076	0.39	6.21	8.76	0.92	8.44	4.68	7.52
103	21	36.376	0.21	-0.81	15.07	0.81	9.56	5.19	8.75
104	21	36.574	0.26	7.12	8.79	1.00	8.00	4.50	7.00
105	21	36.712	0.34	-0.60	12.38	1.13	8.65	4.89	7.52
106	21	36.832	0.33	4.23	11.54	1.39	7.91	4.65	6.53
107	21	36.988	0.35	7.09	9.06	1.15	7.95	4.55	6.79
108	21	37.276	0.30	6.60	11.96	1.15	13.26	7.21	12.11
109	21	37.624	0.32	7.21	8.59	0.99	6.19	3.59	5.21
110	21	37.906	0.32	-0.53	12.97	1.10	8.15	4.63	7.04
111	21	37.942	0.32	6.32	11.25	1.11	8.97	5.04	7.86
112	21	37.984	0.31	7.06	10.73	1.44	8.76	5.10	7.32
113	21	38.104	0.30	-0.20	14.90	1.40	8.60	5.00	7.20
114	21	38.524	0.50	5.67	9.69	1.31	8.89	5.10	7.58
115	21	38.986	0.32	6.96	9.64	0.94	7.85	4.40	6.91
116	21	39.154	0.39	-0.53	10.77	0.70	8.00	4.35	7.30
117	21	39.28	0.42	1.03	11.19	0.81	8.55	4.68	7.73
118	21	39.748	0.34	3.17	13.40	1.04	8.33	4.68	7.29
119	21	39.844	0.30	4.78	11.53	1.10	8.00	4.55	6.91
120	21	40.084	0.23	3.02	12.83	1.28	8.00	4.64	6.72
121	21	40.204	0.43	6.74	7.58	1.29	9.03	5.16	7.74
122	21	40.306	0.30	5.54	9.68	1.31	8.00	4.66	6.70
123	21	40.522	0.37	6.45	8.88	1.56	8.63	5.10	7.07
124	21	40.912	0.36	5.86	9.27	1.07	8.00	4.53	6.93
125	21	41.212	0.40	5.93	9.25	1.30	8.00	4.65	6.70
126	21	41.404	0.26	5.81	8.82	1.21	8.86	5.03	7.65
127	21	41.776	0.32	5.08	10.54	1.17	7.65	4.41	6.48
128	21	42.034	0.46	5.43	8.72	0.88	7.57	4.23	6.69
129	21	42.328	0.30	5.72	8.29	1.08	8.49	4.78	7.41
130	21	42.448	0.27	5.83	10.98	1.17	8.49	4.83	7.32
131	21	42.658	0.31	5.88	10.07	1.10	8.00	4.55	6.90

132	21	42.79	0.22	5.86	8.70	1.16	10.59	5.88	9.43
133	21	43.006	0.33	5.07	9.83	1.05	8.18	4.62	7.13
134	21	43.066	0.32	4.84	9.78	1.25	7.03	4.14	5.77
135	21	43.108	0.32	4.65	11.47	1.00	7.33	4.16	6.33
136	21	43.282	0.31	-0.04	12.05	0.75	7.77	4.26	7.02
137	21	43.402	0.33	5.71	7.77	0.75	7.97	4.36	7.22
138	21	43.612	0.34	5.47	8.23	0.90	7.15	4.03	6.25
139	21	43.69	0.39	4.93	8.29	0.71	9.11	4.91	8.39
140	21	43.978	0.37	-1.11	13.11	0.91	7.20	4.05	6.29
141	21	44.02	0.38	5.51	8.37	0.76	6.66	3.71	5.90
142	21	44.206	0.16	5.92	6.61	1.07	8.58	4.82	7.51
143	21	44.506	0.54	5.16	10.30	0.93	7.16	4.04	6.23
144	21	44.62	0.45	-0.45	12.44	1.31	8.21	4.76	6.91
145	21	44.764	0.20	6.10	8.92	1.36	8.10	4.73	6.75
146	21	44.944	0.20	5.22	8.94	1.28	7.67	4.48	6.39
147	21	45.196	0.60	4.34	11.26	1.12	8.00	4.56	6.88
148	21	45.73	0.51	-0.20	11.53	1.22	8.30	4.76	7.08
149	21	46.138	0.29	4.67	17.99	1.20	8.75	4.97	7.55
150	21	46.414	0.50	5.20	8.08	0.91	7.87	4.39	6.96
151	21	46.726	0.58	4.50	10.72	0.90	9.36	5.13	8.46
152	22	14.556	0.67	0.83	10.45	2.30	10.36	6.33	8.07
153	22	14.778	0.12	7.13	8.84	1.61	8.39	5.00	6.77
154	22	15	0.19	5.93	7.80	2.50	8.00	5.25	5.50
155	22	15.336	0.52	4.51	12.06	1.36	8.00	4.68	6.64
156	22	15.804	0.23	5.20	11.17	1.24	8.00	4.62	6.76
157	22	16.044	0.40	7.23	9.29	1.30	9.21	5.26	7.92
158	22	16.524	0.24	-0.58	15.74	1.21	9.19	5.20	7.98
159	22	16.656	0.28	7.23	10.25	1.08	8.72	4.90	7.65
160	22	17.178	0.56	4.61	10.05	1.25	8.00	4.62	6.75
161	22	17.46	0.10	4.65	13.94	0.98	8.41	4.69	7.44
162	22	17.556	0.16	7.39	9.00	0.84	8.42	4.63	7.57
163	22	17.67	0.11	7.71	9.87	1.59	12.28	6.94	10.69
164	22	17.814	0.10	7.44	11.54	1.55	9.61	5.58	8.06
165	22	18.09	0.41	7.08	9.06	1.00	7.32	4.16	6.32
166	22	18.378	0.20	-0.48	13.49	0.93	8.97	4.95	8.04
167	22	18.426	0.19	-0.86	13.32	1.05	8.25	4.65	7.20
168	22	18.768	0.46	5.28	8.96	1.08	8.93	5.01	7.85
169	22	18.936	0.37	5.43	7.33	1.32	8.48	4.90	7.16
170	22	19.044	0.45	5.13	8.15	1.11	9.20	5.15	8.09
171	22	19.176	0.22	6.08	9.47	1.20	8.26	4.73	7.06
172	22	19.302	0.23	2.65	14.68	0.99	8.55	4.77	7.56
173	22	19.638	0.14	6.16	11.79	0.75	8.00	4.38	7.25
174	22	19.932	0.52	4.58	9.39	1.17	8.00	4.58	6.83
175	22	20.37	0.19	7.52	9.83	1.09	8.87	4.98	7.79
176	22	20.586	0.26	7.41	9.13	1.10	10.29	5.70	9.19
177	22	20.676	0.24	7.28	9.83	1.33	8.93	5.13	7.59
178	22	20.94	0.40	2.79	13.59	1.04	6.98	4.01	5.94
179	22	21.372	0.35	6.94	11.56	1.09	8.00	4.55	6.91
180	22	21.516	0.23	5.94	14.82	1.30	9.57	5.43	8.27
181	22	21.612	0.23	7.62	9.60	1.22	9.61	5.41	8.39
182	22	21.636	0.23	7.67	8.91	2.37	11.39	6.88	9.02
183	22	21.792	0.23	7.19	8.95	1.14	8.04	4.59	6.90
184	22	22.104	0.43	5.24	10.96	1.10	9.04	5.07	7.94
185	22	22.38	0.12	-0.55	14.64	1.13	8.97	5.05	7.84
186	22	22.722	0.46	6.64	11.38	0.91	8.26	4.59	7.35
187	22	22.854	0.26	7.03	11.14	1.12	9.54	5.33	8.43
188	22	23.13	0.05	7.61	10.16	1.11	9.45	5.28	8.35
189	22	23.442	0.53	-0.52	13.07	0.97	9.40	5.18	8.44
190	22	23.592	0.23	7.47	9.00	1.20	8.18	4.69	6.98
191	22	24.126	0.40	5.91	12.20	1.08	7.72	4.40	6.64
192	22	24.15	0.40	7.20	9.40	1.13	9.15	5.14	8.02
193	22	24.606	0.40	4.51	15.05	1.50	10.29	5.89	8.80
194	22	24.846	0.11	7.71	11.17	1.25	11.33	6.29	10.09

195	22	25.266	0.51	6.83	11.37	1.12	7.64	4.38	6.52
196	22	25.638	0.18	7.48	9.44	1.14	8.69	4.91	7.55
197	22	25.752	0.30	7.21	15.40	1.20	9.30	5.25	8.10
198	22	25.86	0.31	6.92	13.52	1.20	9.71	5.46	8.50
199	22	26.22	0.31	6.68	11.23	1.02	7.61	4.31	6.59
200	22	26.454	0.51	7.05	10.61	1.08	9.78	5.43	8.70
201	22	26.802	0.25	7.27	10.76	1.18	9.86	5.52	8.68
202	22	27.102	0.33	7.66	10.24	1.43	11.00	6.21	9.57
203	22	27.468	0.26	7.33	10.90	0.92	9.16	5.04	8.24
204	22	27.576	0.33	3.90	14.25	0.89	11.13	6.01	10.24
205	22	27.996	0.32	4.73	11.79	1.11	10.26	5.69	9.15
206	22	28.152	0.46	6.75	12.46	1.28	9.11	5.19	7.83
207	22	28.494	0.31	7.44	9.17	0.86	9.31	5.08	8.45
208	22	28.674	0.13	8.34	12.34	1.18	10.40	5.79	9.22
209	22	28.806	0.15	8.00	10.93	0.70	12.74	6.72	12.05
210	22	29.046	0.37	6.95	12.76	0.89	10.57	5.73	9.68
211	22	29.184	0.30	6.27	12.52	1.03	9.62	5.33	8.58
212	22	29.304	0.29	5.99	11.65	1.11	8.44	4.77	7.33
213	22	29.436	0.28	7.55	10.39	1.60	9.97	5.78	8.36
214	22	29.538	0.27	5.86	13.04	1.15	8.99	5.07	7.85
215	22	29.76	0.43	7.37	8.76	1.12	8.22	4.67	7.10
216	22	30.084	0.25	7.50	8.99	1.04	7.23	4.14	6.19
217	22	30.306	0.27	7.24	9.45	1.18	10.74	5.96	9.56
218	22	30.408	0.27	6.43	14.38	1.13	8.52	4.83	7.39
219	22	30.546	0.21	1.66	13.29	1.03	9.88	5.45	8.85
220	22	30.672	0.31	7.20	12.64	1.13	9.31	5.22	8.18
221	22	30.804	0.32	6.00	15.16	1.27	9.38	5.33	8.10
222	22	30.966	0.32	6.31	14.31	1.32	10.31	5.82	8.99
223	22	31.374	0.43	6.53	11.40	1.22	9.67	5.44	8.45
224	22	31.668	0.21	7.20	11.77	1.48	10.76	6.12	9.27
225	22	31.752	0.21	7.23	10.54	1.20	8.39	4.79	7.19
226	22	31.872	0.24	7.69	13.21	1.60	17.86	9.73	16.27
227	22	32.154	0.39	6.95	10.01	1.29	9.03	5.16	7.74
228	22	32.298	0.20	7.44	10.00	1.42	9.76	5.59	8.34
229	22	32.466	0.38	6.17	11.71	1.35	8.68	5.02	7.33
230	22	32.718	0.16	7.26	10.98	1.80	9.05	5.42	7.25
231	22	32.832	0.21	7.07	12.10	1.41	9.78	5.59	8.37
232	22	32.958	0.27	7.64	10.05	1.52	10.38	5.95	8.86
233	22	33.084	0.27	-0.50	15.83	1.36	13.01	7.19	11.65
234	22	33.192	0.31	7.22	10.83	1.42	10.68	6.05	9.26
235	22	33.666	0.34	6.39	12.37	1.36	9.34	5.35	7.98
236	22	33.786	0.36	6.70	10.44	1.23	8.00	4.61	6.77
237	22	33.996	0.28	4.43	11.68	1.10	10.01	5.55	8.91
238	22	34.134	0.27	-0.25	12.21	1.16	10.54	5.85	9.38
239	22	34.29	0.43	7.19	9.78	0.84	9.04	4.94	8.20
240	22	34.962	0.49	6.86	11.59	1.04	7.85	4.44	6.81
241	22	35.226	0.22	7.43	12.19	1.04	10.39	5.72	9.35
242	22	35.574	0.21	3.70	14.63	1.22	9.43	5.32	8.21
243	22	35.76	0.31	7.14	9.75	1.11	9.48	5.30	8.37
244	22	35.802	0.34	3.34	12.22	1.19	8.77	4.98	7.58
245	22	35.976	0.34	7.42	9.37	1.13	8.29	4.71	7.17
246	22	36.09	0.40	7.05	10.65	1.06	9.96	5.51	8.91
247	22	36.312	0.21	7.37	9.98	1.03	8.60	4.82	7.57
248	22	36.384	0.18	7.30	10.15	0.83	7.96	4.40	7.13
249	22	36.42	0.18	7.22	11.85	0.98	8.81	4.89	7.83
250	22	36.636	0.27	7.36	8.85	1.00	9.07	5.03	8.07
251	22	36.87	0.38	5.51	11.71	0.89	6.54	3.71	5.65
252	22	36.924	0.39	6.85	9.90	1.33	8.14	4.73	6.81
253	22	37.386	0.29	6.99	11.78	1.03	9.64	5.34	8.60
254	22	37.416	0.30	6.93	11.46	1.07	9.67	5.37	8.60
255	22	37.668	0.28	5.08	12.24	1.27	7.98	4.63	6.70
256	22	37.95	0.42	6.61	11.34	0.82	7.89	4.36	7.07
257	22	38.298	0.26	7.33	9.20	1.10	8.71	4.90	7.61

258	22	38.622	0.37	7.41	9.72	1.14	9.26	5.20	8.12
259	22	39.006	0.19	7.28	11.33	1.11	8.69	4.90	7.58
260	22	39.258	0.55	7.44	9.74	0.84	9.66	5.25	8.82
261	22	39.504	0.06	7.82	9.66	1.28	9.34	5.31	8.06
262	22	39.6	0.06	7.89	10.31	0.99	10.17	5.58	9.18
263	22	39.906	0.41	6.93	10.20	0.92	8.00	4.46	7.08
264	22	39.966	0.43	6.68	12.44	0.78	9.21	4.99	8.42
265	22	40.11	0.25	6.85	9.49	0.72	11.31	6.01	10.59
266	22	40.248	0.18	6.90	11.15	0.89	12.28	6.59	11.39
267	22	40.476	0.38	6.86	10.98	0.99	8.27	4.63	7.28
268	22	40.782	0.18	7.48	8.52	1.08	8.02	4.55	6.94
269	22	41.094	0.52	3.01	12.55	1.01	7.17	4.09	6.16
270	22	41.43	0.13	7.94	11.65	0.76	10.42	5.59	9.66
271	22	41.616	0.29	4.04	13.01	0.98	9.12	5.05	8.13
272	22	41.724	0.40	7.07	10.44	1.03	7.61	4.32	6.58
273	22	41.868	0.40	6.36	12.17	1.11	8.33	4.72	7.22
274	22	41.982	0.33	5.83	12.27	1.13	10.10	5.61	8.97
275	22	42.276	0.08	7.83	10.52	1.14	14.76	7.95	13.62
276	22	42.564	0.43	6.57	10.63	0.98	8.47	4.73	7.48
277	22	42.84	0.33	4.16	14.07	0.96	8.82	4.89	7.87
278	22	42.984	0.33	7.47	9.13	0.98	10.24	5.61	9.26
279	22	43.35	0.37	2.74	12.74	1.13	8.00	4.56	6.87
280	22	43.512	0.34	6.86	9.51	1.61	9.83	5.72	8.23
281	22	43.914	0.24	7.27	9.04	0.90	8.87	4.88	7.97
282	22	44.184	0.51	7.14	9.75	0.85	8.65	4.75	7.81
283	22	44.508	0.06	7.76	9.57	0.89	9.74	5.31	8.85
284	22	44.772	0.26	5.67	16.32	0.98	9.10	5.04	8.12
285	22	44.988	0.44	6.62	11.01	1.21	8.53	4.87	7.32
286	22	45.108	0.30	4.07	11.87	1.00	8.65	4.82	7.65
287	22	45.204	0.25	6.49	11.62	0.92	8.09	4.50	7.17
288	22	45.222	0.25	6.75	11.95	0.79	8.00	4.39	7.21
289	22	45.606	0.26	7.19	9.93	1.33	10.64	5.98	9.31
290	22	45.75	0.27	6.94	10.91	1.40	9.81	5.60	8.40
291	22	45.93	0.35	5.21	12.35	1.32	8.69	5.00	7.37
292	22	45.978	0.31	6.41	12.04	1.21	8.99	5.10	7.78
293	22	46.092	0.33	7.53	10.23	1.92	11.80	6.86	9.88
294	22	46.152	0.29	6.78	15.04	1.39	8.65	5.02	7.26
295	22	46.494	0.19	7.28	10.17	1.39	9.52	5.46	8.14
296	22	46.74	0.31	6.64	11.17	1.18	8.00	4.59	6.82
297	22	46.932	0.29	5.18	13.29	1.28	8.95	5.11	7.66
298	22	47.052	0.31	1.61	13.33	1.39	8.83	5.11	7.44
299	22	47.202	0.30	6.31	10.74	1.31	9.12	5.21	7.81
300	22	47.508	0.36	6.35	8.88	1.59	8.89	5.24	7.30
301	22	47.592	0.36	5.43	11.78	1.34	8.41	4.88	7.07
302	22	47.748	0.21	6.41	12.84	1.19	8.77	4.98	7.58
303	22	47.862	0.25	6.70	10.18	1.37	9.15	5.26	7.79
304	22	48.042	0.18	7.74	9.18	1.41	11.40	6.40	9.99
305	22	48.192	0.31	7.09	9.94	1.48	8.58	5.03	7.10
306	22	48.282	0.28	6.71	10.64	0.96	9.36	5.16	8.40
307	22	48.414	0.24	5.67	10.07	1.08	9.24	5.16	8.16
308	22	48.576	0.33	-0.14	11.75	0.12	12.60	6.36	12.48
309	22	48.684	0.26	4.95	11.28	1.14	8.23	4.68	7.08
310	22	48.828	0.17	0.19	14.91	1.15	8.27	4.71	7.12
311	22	48.948	0.23	7.48	8.31	1.01	8.86	4.94	7.85
312	22	49.302	0.70	2.59	9.56	1.09	8.81	4.95	7.73
